# Comparison of 2 Doses vs 1 Dose in the First Season Children Are Vaccinated Against Influenza: A Systematic Review and Meta-Analysis

**DOI:** 10.1001/jamanetworkopen.2025.35250

**Published:** 2025-10-03

**Authors:** Jessie J. Goldsmith, Sarah Tavlian, Christy Vu, Annette K. Regan, Katherine B. Gibney, Patricia Therese Campbell, Sheena G. Sullivan

**Affiliations:** 1 Department of Infectious Diseases, University of Melbourne, at the Peter Doherty Institute for Infection and Immunity, Melbourne, Australia; 2 WHO Collaborating Centre for Reference and Research on Influenza, Royal Melbourne Hospital, at the Peter Doherty Institute for Infection and Immunity, Melbourne, Australia; 3 Department of Research & Evaluation, Kaiser Permanente Southern California, Pasadena, California; 4 Department of Epidemiology, Fielding School of Public Health, University of California, Los Angeles; 5 Practical Causal Inference Lab, University of California, Los Angeles; 6 School of Nursing and Health Professions, University of San Francisco, San Francisco, California; 7 Victorian Infectious Diseases Service, Royal Melbourne Hospital, at the Peter Doherty Institute for Infection and Immunity, Melbourne, Australia; 8 School of Clinical Sciences, Monash University, Clayton, Australia

## Abstract

**Importance:**

Based on the findings of immunogenicity studies, the World Health Organization has recommended influenza vaccine–naive children younger than 9 years receive 2 doses of influenza vaccine in the first year of vaccination.

**Objective:**

To estimate the increase in protection associated with the second dose of influenza vaccine for influenza vaccine–naive children younger than 9 years.

**Data Sources:**

MEDLINE, EMBASE, and CINAHL were searched for articles published from inception to March 24, 2025.

**Study Selection:**

Peer-reviewed studies that reported vaccine efficacy or vaccine effectiveness against influenza for influenza vaccine–naive children aged 6 months to younger than 9 years by dose number were included. Modeling and cost-effectiveness studies were excluded.

**Data Extraction and Synthesis:**

Two reviewers independently screened and extracted data and assessed studies’ risk of bias. The study followed Preferred Reporting Items for Systematic Reviews and Meta-Analyses (PRISMA) reporting guideline.

**Main Outcomes and Measures:**

For studies reporting both 1 and 2 dose estimates, the difference in vaccine efficacy or effectiveness was calculated in order to estimate the additional protection associated with the second dose. Pooled vaccine efficacy or effectiveness and difference were calculated separately for inactivated influenza and live attenuated influenza vaccines.

**Results:**

There were 51 studies with 415 050 participants included in this analysis. The pooled absolute increase in vaccine effectiveness of a second inactivated influenza vaccine dose in the first year of vaccination was 15 percentage points (pp) (95% CI, −2.8 pp to 33 pp) for those younger than 9 years and 28 pp (95% CI, 4.7 pp to 51 pp) for children younger than 3 years. Insufficient estimates were available to assess the incremental benefit associated with a second dose of live attenuated influenza vaccine.

**Conclusions and Relevance:**

In this systematic review and meta-analysis of influenza vaccine efficacy and effectiveness in the first year of vaccination, receiving 2 doses of inactivated influenza vaccine was associated with improved protection for children younger than 3 years compared with those who receive one dose; however, when the age range was broadened to younger than 9 years, the second dose of inactivated influenza vaccine was not significantly associated with increased protection. Additional high-quality studies are needed to assess the impact of the 2-dose schedule for both vaccine types by age to determine the age range for which a 2-dose schedule is beneficial.

## Key Points

**Question:**

Does research evidence support a 2-dose schedule in the first year of vaccination for influenza vaccine–naive children younger than 9 years?

**Findings:**

This meta-analysis including 51 studies with 415 050 participants estimated a statistically significant increase in vaccine effectiveness (28 percentage points) of a second inactivated influenza vaccine dose in the first year of vaccination for children younger than 3 years. However, there was no significant increase when the age range was broadened to children younger than 9 years.

**Meaning:**

Our findings suggest the second dose of inactivated influenza vaccine confers additional protection for influenza vaccine–naive children younger than 3 years but that the benefit attenuates with age.

## Teaser

This systematic review and meta-analysis estimates the difference in vaccine efficacy and vaccine effectiveness for 1 vs 2 doses of influenza vaccine among children younger than 9 years who have not previously been vaccinated against influenza.

## Introduction

Influenza causes considerable pediatric morbidity and mortality. An estimated 20% of children are infected each year^1^ and children younger than 5 years have the highest hospitalization rates for influenza of any age group.^2^ Children aged under 5 years are critical for influenza transmission^3,4^ and are, therefore, prioritized for influenza vaccination in many countries.

The World Health Organization recommends that influenza vaccine–naive children aged under 9 years receive 2 doses of influenza vaccine, at least 4 weeks apart, with 1 dose each year thereafter.^1^ Evidence supporting this recommendation is based on immunogenicity studies which show influenza vaccine–naive children require 2 doses to reach the seropositivity threshold that corresponds to a 50% reduction in the risk of contracting influenza in adults.^5,6,7^ It is theorized that 2 doses are necessary for influenza vaccine–naive children because they may also be influenza infection–naive and therefore do not benefit from the boosting of naturally acquired antibodies.^8^ However, the evidence supporting the seropositivity threshold for children is limited.^6^

Two systematic reviews, Wall et al^5^ and Kalligeros et al,^9^ sought to compare the vaccine effectiveness (VE) of full vaccination (2 doses) with partial vaccination (1 dose) in children under age 9 years. However, Wall et al’s study^5^ did not include a meta-analysis and Kalligeros et al’s study^9^ was limited to test-negative studies of severe influenza (hospitalization) (VE of partial vaccination: 33.9% [95% CI, 21.1%-46.7%] and VE of full vaccination: 61.8% [95% CI, 54.5%-69.1%]). Most importantly, neither review restricted the included studies to populations of previously influenza vaccine–naive children, meaning these studies do not fully align with immunization policy. Many of the studies included in each review assumed that children who were unvaccinated in the current season had never been vaccinated and fully vaccinated (receipt of 2 or more doses over 1 or more seasons) was equivalent to a previously unvaccinated child who had received 2 doses in the current season. Consequently, studies included in these reviews and their associated reviews are vulnerable to misclassification bias.

The clinical impact of the 2-dose schedule for children being vaccinated against influenza for the first time remains unclear. This review investigates whether vaccine efficacy and effectiveness studies support the recommended 2-dose schedule for influenza vaccine–naive children.

## Methods

We conducted a systematic review and meta-analysis of studies reporting influenza vaccine efficacy or effectiveness in previously influenza vaccine–naive children by number of doses received (PROSPERO identifier: CRD42023460374). Our review was reported according to Preferred Reporting Items for Systematic Reviews and Meta-Analyses (PRISMA) reporting guidelines (eTable 6 in Supplement 1).

### Eligibility and Exclusion Criteria

The population for this review was children aged 6 months up to 9 years who had not been vaccinated against influenza prior to the season(s) under study (ie, influenza vaccine–naive). The intervention of interest was the number of influenza vaccine doses, and the comparator of interest was influenza vaccine–naive children. For efficacy and effectiveness studies of pandemic A(H1N1)pdm09 influenza monovalent vaccines, children were classified as influenza vaccine–naive if they had never received a vaccine containing A(H1N1)pdm09 and the outcome was infection with A(H1N1)pdm09 (as distinct from prior infection or vaccination with seasonal A[H1N1]). The primary outcome was VE or efficacy, by the number of doses, against laboratory-confirmed influenza. There was no limit on the date of publication or language, provided an English language title and abstract were available. Modeling and cost-effectiveness studies, as well as studies that had not been peer reviewed and conference abstracts, were excluded.

### Search Strategy and Risk of Bias Assessment

The search strategy used terms synonymous with *influenza*, *children*, *vaccine*, and *effectiveness or efficacy* to identify potentially relevant studies (eTable 1 in Supplement 1). We searched EMBASE, Medline OVID, and CINAHL databases from inception on September 14, 2023, and March 24, 2025.

Search results were imported into systemic review software (Covidence) for study selection and data extraction. After removal of duplicates, 2 reviewers (J.G. and C.V.) independently evaluated studies for inclusion. A predefined decision tree was used to support a consistent approach to full-text screening by reviewers (eFigure 1 in Supplement 1). Studies in languages other than English were evaluated for inclusion by individuals fluent in the language.

Reference lists of relevant systematic reviews and eligible studies were also searched. Two reviewers (J.G. and S.T.) independently undertook data extraction. Information extracted from each selected study included influenza vaccination history, number of doses of influenza vaccine received in the study year, and final sample size. Vaccine efficacy or effectiveness estimates were extracted from each study for multiseason and seasonal estimates for any age group under 9 years for all influenza and influenza by type, subtype, or lineage. Reviewers used a customized form for data extraction (eAppendix 1 in Supplement 1) and study authors were contacted when additional data were required.

Two reviewers (J.G. and S.T.) independently evaluated the included studies for risk of bias. The revised Cochrane risk of bias tool (RoB2)^10^ was used to assess randomized controlled trials (RCTs) while the ROBINS-I tool (Risk of Bias in Nonrandomized Studies of Interventions)^11^ was used to assess observational studies. Where disagreements occurred in study identification, data extraction, and bias analysis, discussions were held to reach consensus and, where necessary, a third reviewer (S.S.) was involved.

### Statistical Analysis

Studies were grouped according to design (vaccine efficacy or effectiveness). VE studies were further classified as naive studies (ie, all exposure groups were previously influenza vaccine–naive) and mixed-history studies (ie, only children classified as partially vaccinated were previously influenza vaccine–naive). This was a pragmatic decision motivated by concern that the mixed-history studies were vulnerable to bias (eAppendix 4 in Supplement 1). Studies were described according to their grouping, vaccine type, source of vaccination history, study design, and source population. Vaccine efficacy and effectiveness estimates were classified by intervention as 1 dose or 2 doses for vaccine efficacy and naive VE studies and partially vaccinated or fully vaccinated for mixed-history VE studies using the definitions in Table 1. Estimates from interventions that did not meet these definitions were excluded from the meta-analysis.

**Table 1.** Intervention Definitions

Study group	Intervention	Intervention group	Reference group
Vaccine efficacy and naive VE studies	1 Dose	Previously influenza vaccine–naive and received 1 dose in the current season.	Influenza vaccine–naive
	2 Doses	Previously influenza vaccine–naive and received 2 doses in the current season.	
Confounded VE studies	Partially vaccinated	Previously influenza-vaccine-naive and received 1 dose in the current season.	Not vaccinated against influenza in the current season but may have been vaccinated in a prior season.^a^
	Fully vaccinated	Received 2 doses of influenza vaccine in the current season or at least 1 dose in the current season and 1 prior seasons.	

Abbreviation: VE, vaccine effectiveness.

^a^ This was assumed to be the case where the study did not explicitly state the comparison group was influenza vaccine–naive.

Separate meta-analyses were conducted for each of the groups described above by type of vaccine (inactivated influenza vaccine [IIV], live attenuated [LAIV] and monovalent A[H1N1]pdm09) and by intervention (1 dose, 2 doses, partially vaccinated, fully vaccinated). Analyses of IIV were separated from LAIV because IIV can be used from 6 months of age while LAIV is not recommended for children aged less than 2 years. No distinction was made between trivalent and quadrivalent formulations or between full and half antigen doses. However, a distinction was made between monovalent and seasonal multivalent vaccines available for the initial A(H1N1)pdm09 pandemic season because the multivalent vaccines did not contain the A(H1N1)pdm09 strain. Furthermore, adjuvanted monovalent A(H1N1)pdm09 vaccines were separated from nonadjuvanted monovalent A(H1N1)pdm09 vaccines because 90% of children reached the seropositivity threshold following a single pediatric dose of the adjuvanted vaccine.^12^ Estimates that were not vaccine type–specific or, for naive and pandemic studies, assessed to have a critical risk of bias were excluded from the meta-analysis. Mixed-history studies were not excluded if they had a critical risk of bias because these studies were, by definition, at a heightened risk of bias.

If a study estimated the vaccine efficacy or effectiveness for 1 and 2 doses or partially and fully vaccinated within the same season for the same age group, the percentage point (pp) difference between the 2 estimates was calculated as *VE difference = VE_2_ – VE_1_*, where *VE_2_* is the vaccine efficacy or effectiveness for 2 doses or fully vaccinated and *VE_1_* is the vaccine efficacy or effectiveness for 1 dose or partially vaccinated. The 95% CI for VE difference was estimated by bootstrapping 1000 samples for *VE_2_* and *VE_1_*.^13,14^ If multiple estimates were available, estimates with the broadest applicable age range were selected. If both multiseason and seasonal estimates were available, seasonal estimates were selected.

Pooled estimates were calculated using both random-effects and fixed-effects models to check for instability.^15^ Where data were sufficient, subgroup meta-analyses were performed by virus type or subtype, age less than 3 years, and/or season. The lower age limit was a pragmatic choice given the available estimates. Statistical heterogeneity was calculated using Cochran *Q* and the *I^2^*.^16^

Publication bias was assessed using funnel plots for all categories for which there were 4 or more estimates, and funnel plot asymmetry was tested using the Eggar regression test for categories where 10 or more estimates were available.^17^ All data analysis was done in R version 4.4.1 using the Metafor^18^ and Epitools^19^ packages (eAppendix 2 in Supplement 1).

## Results

There were 4452 records identified from searches of EMBASE, Medline OVID, CINAHL, and reviews of reference lists. Of these, 51 were included in this review (eFigure 2 in Supplement 1). The earliest was published in 1998^20^ and the latest in 2024.^21^ Most studies excluded during full-text screening either did not report the results of children younger than 9 years separately or did not account for vaccine history in any study group (eTable 5 in Supplement 1).

**Figure 1.**
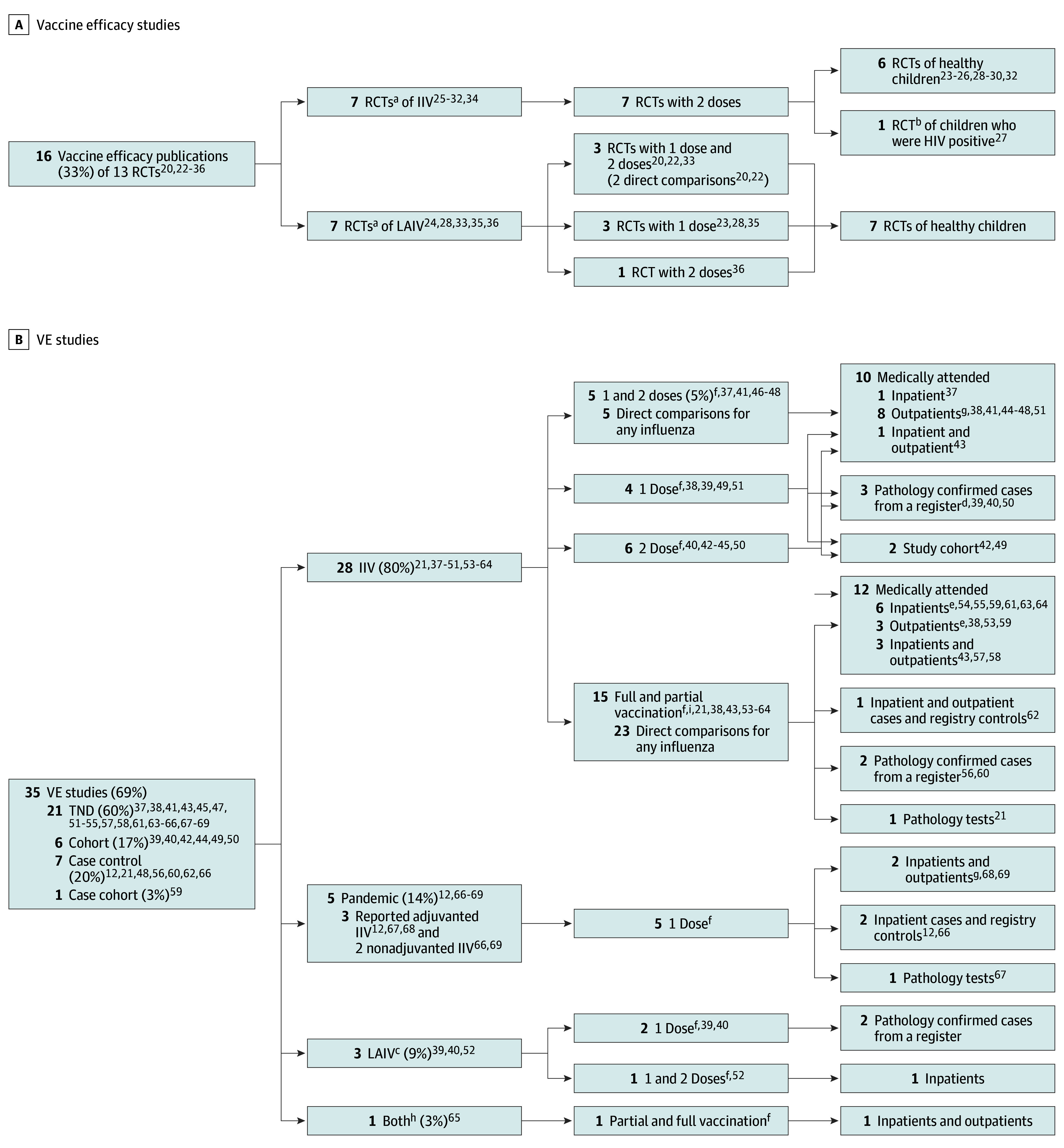
Studies by Study Design, Vaccine, Intervention, and Source Population for Influenza Vaccine Efficacy and Vaccine Effectiveness (VE) ^a^Krishnan et al^28^ reported vaccine efficacy for both inactivated influenza vaccine (IIV) and live attenuated IV (LAIV). ^b^This paper was excluded from meta-analysis because it was the only trial of children with HIV positivity. ^c^These studies^39,40^also reported on IIV VE. ^d^Two separate studies reported on 1-dose^39^ and 2-dose^40^ IIV VE in the same influenza vaccine–naive cohort in 2015-16. ^e^One study^59^ reported separate estimates for inpatient and outpatient populations. ^f^One dose and/or 2 doses intervention denotes estimates where all intervention groups were previously influenza vaccine–naive (naive studies) while partial and full vaccination intervention group denotes estimates where only those classified as partially vaccinated were previously influenza vaccine–naive (mixed-history studies). ^g^One naive study^51^ and 2 pandemic studies^68,69^ were excluded from the meta-analysis because they were judged to be at a critical risk of bias (eFigures 15 and 16 in Supplement 1). ^h^Treanor et al^65^ was excluded from the meta-analysis because VE was not reported by vaccine type. ^i^Two studies^38,43^ reported both naive and mixed history estimates.

Sixteen included publications (33%)^20,22,23,24,25,26,27,28,29,30,31,32,33,34,35,36^ were vaccine efficacy studies that reported results of 13 RCTs (Figure 1A; Table 2). All trials exclusively recruited previously influenza vaccine–naive children. The source of vaccine history was only reported by 1 trial,^30,31^ which ascertained prior exposure to influenza vaccination from parent or guardian report. All^25,26,27,30,31,32,34^ but 1^28^ of the IIV efficacy studies had a lower age limit of 6 months, compared with only 2^22,36^ of the 7^20,22,23,24,28,33,35,36^ LAIV efficacy studies (Table 2).

**Table 2.** Studies Meeting the Inclusion Criteria

Source	Design	Source population	Seasons^a^	Participant age, y	Participants	Influenza types and subtypes	Location^b^	Vaccine types^c^	Vaccine history	Vaccine data source	Meta-analysis
**Vaccine efficacy studies**											
Belshe et al,^20^ 1998	RCT	Trial participants	1996-1997	1.25 to <6	2602	Any, H3N2, B	US	LAIV	Not described in the paper but described by Block et al,^74^ as being influenza vaccine–naive	NR	Included
Bracco Neto et al,^22^2009	RCT	Trial participants	2001	0.5 to <3	3200	Any, H3N2, B, matched	Argentina, Brazil, South Africa	LAIV	Children who had never received an influenza vaccination.	NR	Included
Brooks et al,^23^ 2016 and Rotrosen et al,^24^2017	RCT	Trial participants	2013	2 to <5	1761	Any, H1N1, H3N2, B(Yam), B(Vic), matched	Bangladesh	LAIV	Children who had never received an influenza vaccination	NR	Included
Claeys et al,^25^ 2018 and Dbaibo et al,^26^2020	RCT	Trial participants	2011-2012, 2012, 2012-2013, 2013, 2014	0.5 to <3	12 018	Any, A, H1N1, H3N2, B, B(Yam), B(Vic), matched	Belgium, Bangladesh, Czechia, Dominican Republic, Spanish, UK, Honduras, India, Lebanon, Philippines, Poland, Thailand, Turkey	IIV	99.2% of participants had not been previously vaccinated against influenza	NR	Included
Esposito et al,^27^ 2022	RCT	Trial participants	2017-2018 to 2019	0.5 to <3	2000	Any, H1N1, H3N2, B, matched	Asia and Europe	IIV	Children who had never received an influenza vaccination or been infected with influenza	NR	Included
Krishnan et al,^28^ 2021	RCT	Trial participants	2015, 2016	2 to <5, 3 to <5, 5 to <9	3041	Any	India	IIV, LAIV	All participants assumed vaccine-naive prior to enrolment	NR	Included
Madhi et al,^29^2013	RCT	Trial participants	2009	0.5 to <5	410	Any, H3N2	South Africa	IIV	Children who had never received an influenza vaccination	NR	Excluded only trial of children positive for HIV
Pepin et al,^30,31^ 2019	RCT	Trial participants	2014, 2014-2015, 2015, 2015-2016	0.5 to <3	5806	Any, A, H1N1, H3N2, B, B(Vic), B(Yam), matched	Dominican Republic, Spain, France, Greece, Honduras, Italy, Philippines, Romania, South Africa	IIV	Children who had never received an influenza vaccination or been infected with influenza	Parent or guardian	Included
Rolfes et al,^32^ 2017	RCT	Trial participants	2010-2011, 2011-2012, 2012-2013, 2013-2014	0.5 to <2	4081	Any, matched	Bangladesh	IIV	All participants assumed vaccine-naive prior to enrolment	NR	Included
Tam et al,^33^ 2007	RCT	Trial participants	2000-2001, 2001-2002	1 to <3	3174	Any, H1N1, B, H3N2, matched	China, India, Malaysia, Philippines, Singapore, Thailand, Taiwan	LAIV	Not described in the paper but described by Block et al,^74^ as being influenza-vaccine-naive	NR	Included
Vesikari et al,^36^ 2006	RCT	Trial participants	2000-2001	0.5 to <3	1616	Any, H1N1, B, matched	Belgium, Finland, Israel, Spain, UK	LAIV	Children who had never received an influenza vaccination	NR	Included
Vesikari et al,^34^ 2011	RCT	Trial participants	2007-2008 to 2008-2009	0.5 to <6, 0.5 to <3	4707	Any, B, H3N2	Finland, Germany	aIIV, IIV	Children who had never received an influenza vaccination	NR	Included
Victor et al,^35^ 2016	RCT	Trial participants	2013	2 to <6	1761	Matched	Senegal	LAIV	Post hoc analysis reports VE stratified by prior receipt of IIV in any prior season	NR	Included
**Vaccine effectiveness studies (naive)**											
Baum et al,^40^2020	Cohort	Pathology confirmed cases from a register	2015-2016, 2016-2017, 2017-2018	2 to <3	181 293	Any, A, B	Finland	IIV, LAIV	Children who had never received an influenza vaccination	Immunization register	Included^c^
Chung et al,^41^2020	TND	Outpatients	2014-2015 to 2017-2018	0.5 to <8, 0.5 to <2	2440	Any, H1N1, H3N2, B	US	IIV	Children who had never received an influenza vaccination	Electronic medical records; immunization registries	Included
Heinonen et al,^42^ 2011	Nested cohort	Trial participants	2007-2008	0.75 to <3	631	Any, A, B	Finland	IIV	All children were assumed to be previously-vaccine-naive as prior to 2007 influenza vaccination was not included in the childhood vaccination program and was rarely given	None	Included
Katayose et al,^50^ 2011	Cohort	Inpatients and outpatients	2002-2003, 2003-2004, 2004-2005, 2005-2006, 2006-2007, 2007-2008	0.5 to <1	1247	A, B	Japan	IIV	Age group was too young to be eligible for influenza vaccination in prior seasons	Electronic medical records	Included
Katz et al,^43^2016	Nested TND	Trial participants	2010-2011, 2011-2012, 2012-2013	0.5 to <9	18 000	Any	Kenya	IIV	Population assumed influenza vaccine–naive prior to start of trial as <45 000 doses of influenza vaccine are purchased every year for a population of 44 million	None	Included also included in mixed-history VE meta-analyses
Kildegaard et al,^52^ 2023	TND^d^	Inpatients	2021-2022	0.5 to <6	4377	Any	Denmark	LAIV	Children who had never received an influenza vaccination	Immunization register	Included
Mattila et al,^44^ 2021	Cohort	Pathology confirmed cases from a register	2015-2016	0.5 to <1	431	Any, A, B	Finland	IIV	Age group was too young to be eligible for influenza vaccination in prior seasons	NR	Included
Nohynek et al,^39^ 2016	Cohort	Pathology confirmed cases from a register	2015-2016	2.5 to <3.5	55 258	Any, A, B	Finland	IIV, LAIV	Children who had never received an influenza vaccination	Immunization register	Included
Rao et al,^45^2021	TND	Outpatients	2016-2017 to 2017-2018	0.5 to <2	1252	Any, A, B	US	IIV, mixed	Children who had never received an influenza vaccination	Parents and guardians; immunization information systems; electronic medical records	Included
Shinjoh et al,^46^ 2019	TND	Outpatients	2013-2014 to 2017-2018	0.5 to <1	944	Any, A, B	Japan	IIV	Age group was too young to be eligible for influenza vaccination in prior seasons	Maternal and child health handbooks	Included
Shinjoh et al,^47^ 2021	TND	Outpatients	2018-2019	0.5 to <1	206	A	Japan	IIV	Shinjoh et al,^46^ 2019	Shinjoh et al,^46^ 2019	Included
Shuler et al,^48^ 2017	Case-control	Outpatients	2003-2004	0.5 to <5, 0.5 to <2	870	Any	US	IIV	Current season vaccinations and unvaccinated are stratified by vaccination status in the prior season	Medical practitioners; health records	Included
Sofia Arriola et al,^37^ 2019	TND	Inpatients	2013-2017	0.5 to <2	2389	Any, H1N1, H3N2, B	Argentina, Brazil, Chile, Colombia, Paraguay	IIV	Children who had never received an influenza vaccination	Vaccination cards; electronic immunization registries	Included
Thompson et al,^38^ 2016	TND	Outpatients	2011-2012 to 2012-2013	0.5 to <9	2768	Any, H3N2, B	US	IIV	Children may have been previously vaccinated against influenza if classed as fully vaccinated or unvaccinated	Immunization register	Included also included in mixed-history VE meta-analyses
Wagner et al,^49^ 2022	Cohort	Nicaraguan Influenza Birth Cohort	2012-2016	0.5 to <2	742	Any	Nicaragua	IIV	Children who had never received an influenza vaccination	Parents and guardians; vaccination cards; health records	Included
**Vaccine effectiveness studies (mixed-history)**											
Blyth et al,^53^ 2014	TND	Inpatients	2008-2012 (excludes 2009)	0.5 to <5	1515	Any	Australia	IIV	Children may have been previously vaccinated against influenza if classed as fully vaccinated. No definitions provided for partially vaccinated and unvaccinated	Immunization register; immunization providers	Included
Buchan et al,^54^ 2017	TND	Inpatients	2010-2011, 2011-2012, 2012-2013, 2013-2014	0.5 to <5, 0.5 to <2	9982	Any, A, B, H1N1, H3N2	Canada	IIV	Children may have been previously vaccinated against influenza if classed as fully vaccinated. No definition provided for unvaccinated	Ontario Health Insurance Plan database	Included
Chua et al,^55^ 2019	TND	Inpatients	2011-2012 to 2018-2019	0.5 to <9, 0.5 to <3	23 187	Any, H1N1, H3N2, B	China	IIV	Children may have been previously vaccinated against influenza if classed as fully vaccinated. No definition provided for unvaccinated	Parent or guardian and verified using immunization and electronic health records or by contacting vaccine providers	Included
Cochran et al,^56^ 2010	Case-control	Pathology confirmed cases from a register	2003-2004, 2004-2005, 2005-2006	0.5 to <2	1648	Any	US	IIV	Children may have been previously vaccinated against influenza if classed as fully vaccinated or unvaccinated	KPNC immunization tracking system	Included
Eisenberg et al,^57^ 2008	TND	Inpatients and outpatients	2003-2004, 2004-2005	0.5 to <6, 0.5 to <2	2474	Any	US	IIV	Children may have been previously vaccinated against influenza if classed as fully vaccinated or unvaccinated	Vaccination cards; medical records	Included
Shrestha et al,^21^ 2024	Case-control	Pathology confirmed cases and COVID-19 negative controls	2022	0.5 to <9, 0.5 to <2.5	17 646	Any	Australia	IIV	Children may have been previously vaccinated against influenza if classed as fully vaccinated or unvaccinated	Immunization register	Included
Staat et al,^58^ 2011	TND	Inpatients and outpatients	2005-2006, 2006-2007	0.5 to <5, 0.5 to <2	528	Any	US	IIV	Children may have been previously vaccinated against influenza if classed as fully vaccinated or unvaccinated	Medical practitioners; health records; immunization records	Included
Szilagyi et al,^59^ 2008	Case-cohort	Inpatients and outpatients	2003-2004, 2004-2005	0.5 to <5, 0.5 to <2	9220	Any	US	IIV	Children may have been previously vaccinated against influenza if classed as fully vaccinated or unvaccinated	Health record	Included
Thangara-jah et al,^60^ 2021	Case-control	Pathology confirmed cases from a register	2018	0.5 to <6, 0.5 to <2, 2 to <3	11 770	Any, A, H1N1, H3N2, B	Australia	IIV	Children may have been previously vaccinated against influenza if classed as fully vaccinated or unvaccinated	Immunization record	Included
Treanor et al,^65^ 2012	TND	Inpatients and outpatients	2010-2011	0.5 to <9	2122	Any	US	Mixed	Children may have been previously vaccinated against influenza if classed as fully vaccinated. No definition provided for unvaccinated	Health records; immunization records	Excluded VE by vaccine type not reported
Wang et al,^61^ (2016)	TND	Inpatients and outpatients	2011-2012	0.5 to <3	584	Any	China	IIV	Children may have been previously vaccinated against influenza if classed as fully vaccinated or unvaccinated	Immunization record	Included
Yang et al,^62^ 2012	Case-control	Inpatient and inpatient cases and registry controls	2009, 2010	0.5 to <5, 0.5 to <2	7068	Any	China	IIV	Children may have been previously vaccinated against influenza if classed as fully vaccinated or unvaccinated	Immunization record	Included^e^
Yeung et al,^63^ 2018	TND	Inpatients	2014-2015 to 2015-2016	0.5 to <6	2900	Any, A, B	China	IIV	Children may have been previously vaccinated against influenza if classed as fully vaccinated or unvaccinated	Parents and guardians and immunization record	Included
Yildirim et al,^64^ 2021	TND	Inpatients	2012-2013 to 2016-2017	0.5 to <9	1024	Any	US	IIV	Children may have been previously vaccinated against influenza if classed as fully vaccinated. No definition provided for unvaccinated	Immunization record	Included
**Vaccine effectiveness studies (pandemic A[H1N1]pdm09)**											
Gaglani et al,^51^ 2016	TND	Outpatients	2013-2014	2 to <9	121	H1N1	US	IIV	Children who had not been previously vaccinated with a pH1N1 containing vaccine	Parents and guardians, health records and immunization records	Excluded critical risk of bias.
Gilca et al,^12^ 2011	Case-control	Inpatient cases and registry controls	2009-2010	0.5 to <9	884	H1N1	Canada	Mono. A(H1N1)pdm09 aIIV	Pandemic vaccine not available prior to November 2009	Provincial health insurance registry	Included
Griffin et al,^69^ 2011	TND	Inpatients and outpatients	2009-2010	0.5 to <9	2168	H1N1	US	Mono. A(H1N1)pdm09 IIV	Pandemic vaccine not available prior to November 2009	Patient or parental report and confirmed by medical record review and/or immunization register	Excluded critical risk of bias
Hadler et al,^66^ 2012	Case-control	Inpatient cases & registry controls	2009-2010	3 to <9	807	H1N1	US	Mono. A(H1N1)pdm09 IIV	Pandemic vaccine not available prior to November 2009	Immunization record	Included
Mahmud et al,^67^ 2011	TND	Pathology tests	2009-2010	0.5 to <3	339	H1N1	Canada	Mono. A(H1N1)pdm09 aIIV	Pandemic vaccine not available prior to November 2009	Immunization register	Included
Van Buynder et al,^68^ 2010	TND	Inpatients and outpatients	2009-2010	0.5 to <5	38	H1N1	Canada	Mono. A(H1N1)pdm09 aIIV	Pandemic vaccine not available prior to November 2009	Immunization register	Excluded critical risk of bias

Abbreviations: IIV, inactivated influenza vaccine; KPNC, Kaiser Permanente Northern California; LAIV, live attenuated influenza vaccine; NR, not reported; RCT, randomized controlled trials; TND, test-negative design; VE, vaccine effectiveness.

^a^ Where multiple seasons are joined by “to,” single season estimates were unavailable.

^b^ LAIV estimates from 2015-2016 were excluded from the meta-analysis because 1-dose LAIV had been previously reported by Nohynek using an overlapping cohort.

^c^ Mixed indicates that VE estimates included children vaccinated with either LAIV or IIV, and aIIV indicates adjuvanted inactivated influenza vaccine.

^d^ TND published in the appendix as a sensitivity analysis.

^e^ Estimates from 2009 were excluded from the meta-analysis because type of vaccine being used (seasonal, monovalent A[H1N1]pdm09, or both) was unclear.

The remaining 35 publications were VE studies (Table 2). Sixteen reported naive VE estimates for seasonal influenza,^37,38,39,40,41,42,43,44,45,46,47,48,49,50,51,52^ 16 mixed-history VE studies for seasonal influenza,^21,38,43,53,54,55,56,57,58,59,60,61,62,63,64,65^ and 5 VE studies for A(H1N1)pdm09 influenza^12,58,66,67,68^ (Figure 1B). Most^21,37,38,39,40,41,42,43,44,45,46,47,48,49,50,53,54,55,56,57,58,59,60,61,62,63,64^ were IIV VE studies^21,37,38,39,40,41,42,43,44,45,46,47,48,49,50,53,54,55,56,57,58,59,60,61,62,63,64^ (28 [80%]) and over half used a test negative design (TND).^37,38,41,43,45,46,47,51,52,53,54,55,57,58,61,63,64,65,67,68,69^ Three studies^39,40,52^ reported VE for 1 dose of LAIV, 2^39,40^ of which were conducted in Finland using the same data source. Immunization records (23 studies^12,21,37,38,39,40,41,45,51,52,53,55,58,60,61,62,63,64,65,66,67,68,69^) were the most common source for vaccination history. One observational study^44^ did not report the source of vaccination history and 2 assumed universal influenza vaccine–naivety based on influenza vaccination availability.^42,43^ No study reported relying solely on parental report for vaccination history (Table 2). All further analysis of the VE studies for A(H1N1)pdm09 influenza is reported in eAppendix 3 in Supplement 1.

### Meta-Analysis of Vaccine Efficacy

#### IIV Vaccines

Five RCTs^25,26,27,28,30,31,32^ with 25 931 participants were included in the meta-analysis of IIV vaccine efficacy. Pooled vaccine efficacy of 2 doses was 52% (95% CI, 43%-60%; *Q* = 44) (eFigure 3 in Supplement 1) for children younger than 9 years and 51% (95% CI, 41%-60%, *Q* = 36) for children younger than 3 years (eFigure 4 in Supplement 1). Point estimates for pooled vaccine efficacy for 2 doses for children younger than 3 years were higher for A(H1N1)pdm09 (63% [95% CI, 44%-75%]; *Q* = 10) than H3N2 (45% [95% CI, 36%-53%]; *Q* = 6) (eFigure 5 in Supplement 1) and higher for B/Yamagata (51% [95% CI, 40% to 61%]; *Q* = 2) than B/Victoria (28% [95% CI, −11% to 53%]; *Q* = 4) (eFigure 6 in Supplement 1). No 1-dose IIV efficacy studies were identified.

#### LAIV Vaccines

**Figure 2.**
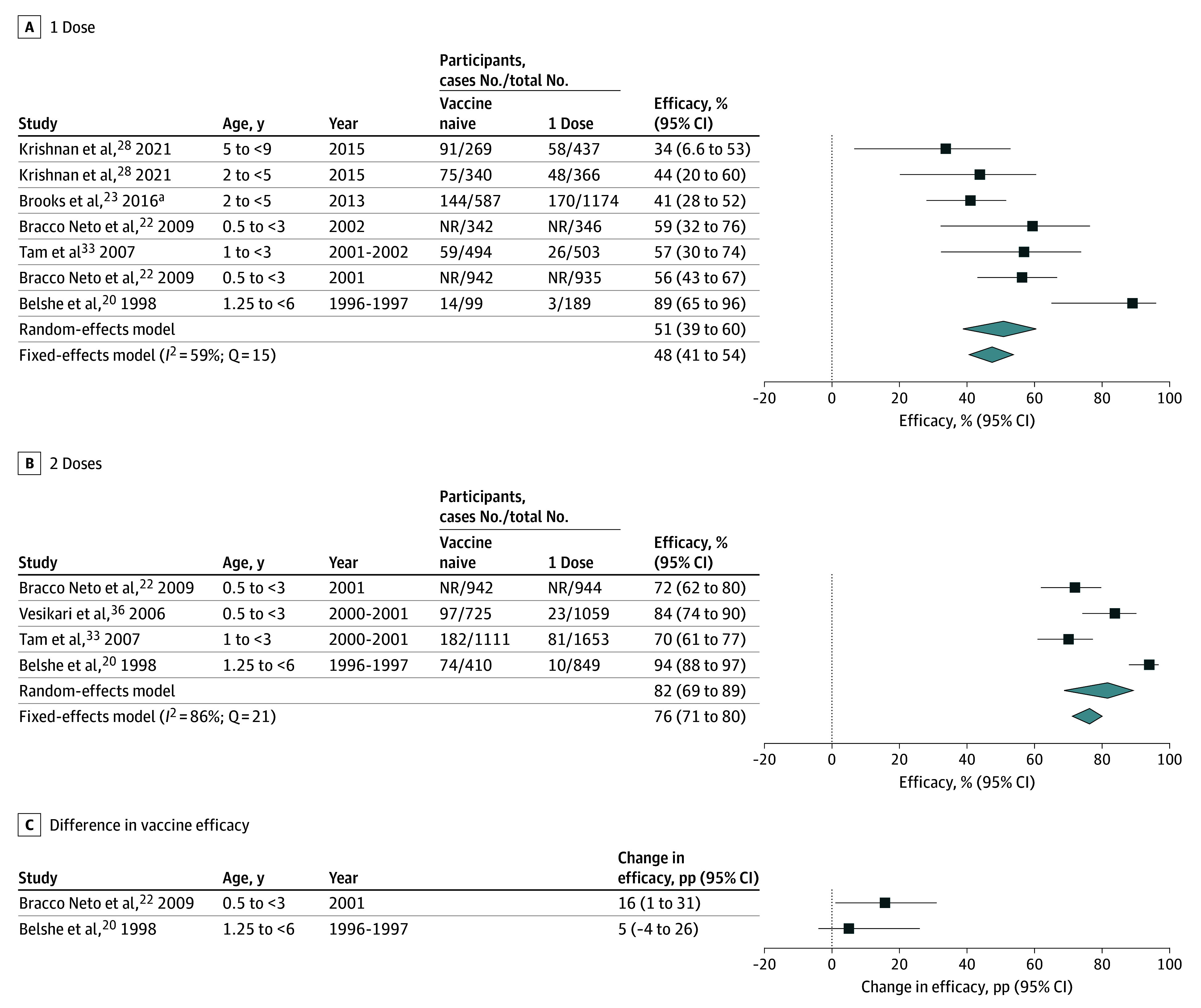
LAIV Efficacy Meta-Analysis for Previously Influenza Vaccine–Naive Children Younger than 9 Years by Doses Received and Difference Between Comparable Estimates LAIV indicates live attenuated influenza vaccine; NR, not reported. The reference group is children younger than 9 years who have never been vaccinated. Difference in vaccine efficacy was calculated as efficacy for 2 doses minus efficacy 1 dose. Difference in vaccine efficacy that is greater than zero indicates greater efficacy for 2 doses. B/Victoria lineage was dominant and not included in the vaccine. Pooled LAIV vaccine efficacy estimates for 1 dose and 2 doses for matched influenza strains was consistent (51% [95% CI, 32%-64%] and 76% [95% CI, 69%-80%]).

Seven RCTs^20,22,23,24,28,33,35,36^ with 16 145 participants were included in the meta-analysis of LAIV vaccine efficacy. Pooled vaccine efficacy against any influenza was 51% (95% CI, 39%-60%; *Q* = 15) for 1 dose and 82% (95% CI, 69%-89%; *Q* = 21) for 2 doses. Two studies compared 1- and 2-dose estimates.^20,22^ Belshe et al^20^ reported efficacy for 1 dose was 89% (95% CI, 65%-96%) and for 2 doses was 94% (95% CI, 88%-97%), leading to change in efficacy of 5 percentage points (pp) (95% CI, −4 pp to 26 pp). Bracco Neto et al^22^ reported efficacy for 1 dose was 56% (95% CI, 43%-67%) and for 2 doses was 72% (95% CI, 62%-80%), leading to change in efficacy of 16 pp (95% CI, 1 pp to 31 pp) (Figure 2). For H3N2 the pooled vaccine efficacy was 60% (95% CI, 48%-70%; *Q* = 3) for 1 dose, 86% (95% CI, 66%-94%; *Q* = 11) for 2 doses. For influenza B the pooled vaccine efficacy was 75% (95% CI, 46%-89%; *Q* = 17) for 2 doses (eFigure 7 in Supplement 1). Insufficient estimates were available to estimate pooled change in efficacy for LAIV, pooled vaccine efficacy for 1 dose for influenza B, or for any other subtype specific estimates.

### Meta-Analysis of Vaccine Effectiveness

#### IIV Vaccines

**Figure 3.**
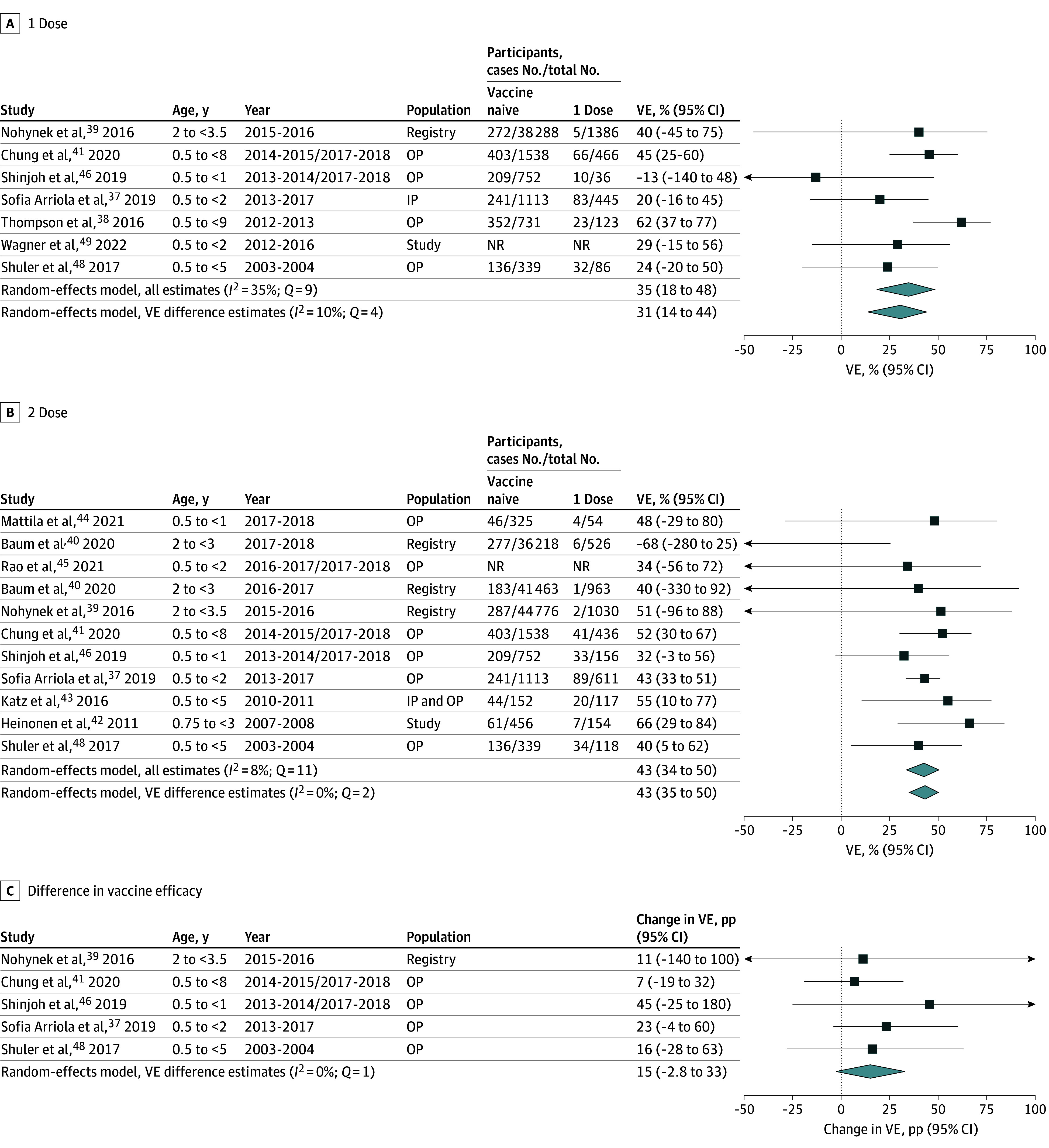
IIV Effectiveness Meta-Analysis for Previously Influenza Vaccine–Naive Children Younger Than 9 Years by Doses Received and Difference Between Comparable Estimates IIV indicates inactivated influenza vaccine; IP, inpatient; NR, not reported; OP, outpatient; VE, vaccine effectiveness. The reference group is children younger than 9 years who have never been vaccinated for influenza. VE difference = *VE_2_ − VE_1_*. A VE difference greater than 0 indicates greater effectiveness for 2 doses.

Twelve studies^37,38,39,40,41,42,43,44,45,46,48,49^ were included in the naive VE meta-analysis for IIV against any influenza. Pooled VE against any influenza was 35% (95% CI, 18%-48%; *Q* = 9) for 1 dose (7 individual estimates) and 43% (95% CI, 34%-50%; *Q* = 1) for 2 doses (11 individual estimates) for vaccine-naive children aged under 9 years. Five studies compared 1- and 2-dose estimates, for which the pooled VE estimates were 31% (95% CI, 14%-44%; *Q* = 4) and 43% (95% CI, 35%-50%; *Q* = 2), respectively, with a pooled difference of change in VE of 15 pp (95% CI, −2.8 pp to 33 pp; *Q* = 1) (Figure 3). When limited to children younger than 3 years, pooled VE for 1 dose dropped to 14% (95% CI, −9.8% to 33%; *Q* = 5) while 2-dose VE remained comparable at 41% (95% CI, 29% to 51%; *Q* = 11) and pooled difference increased to change in VE of 28 pp (95% CI, 4.7 pp to 51 pp; *Q* = 0) (eFigure 8 in Supplement 1).

Pooled VE for IIV against influenza A was 61% (95% CI, 36%-76%; *Q* = 23) for 2 doses. For influenza B pooled IIV, VE was 36% (95% CI, 1.3%-58%; *Q* = 3) for 1 dose and 44% (95% CI, 23%-60%; *Q* = 4) for 2 doses for vaccine-naive children younger than 3 years (eFigure 9 in Supplement 1). Wide confidence intervals associated with the estimates for 1-dose influenza A IIV VE and difference for influenza A and influenza B IIV VE precluded calculation of pooled estimates for these measures. Insufficient estimates were available for subtype-specific comparisons of IIV.

#### LAIV Vaccines

Pooled VE for 1-dose LAIV against any influenza was 26% (95% CI, 6%-42%; *Q* = 0) from 4 individual estimates (eFigure 10 in Supplement 1). Insufficient estimates were available for type or subtype-specific comparisons of LAIV and only 1 study^52^ reported a 2-dose LAIV effectiveness estimate.

#### Mixed Vaccine History

Fifteen studies^21,38,43,53,54,55,56,57,58,59,60,61,62,63,64^ were included in the mixed-history VE meta-analysis for IIV against any influenza. Pooled VE for partially vaccinated children younger than 9 years was 32% (95% CI, 21%-41%; *Q* = 44) and for fully vaccinated children was 54% (95% CI, 45%-62%; *Q* = 73) against any influenza. Fourteen studies provided 23 direct comparisons of partial and full vaccination estimates, for which the pooled VE estimate for partial vaccination was 31% (95% CI, 20%-41%; *Q* = 43) while the VE estimate for full vaccination remained unchanged. The pooled difference between partial and full vaccination VE was 23 pp (95% CI, 14 pp to 32 pp; *Q* = 24) (eFigure 11 in Supplement 1). When limited to children younger than 3 years, pooled VE for partial vaccination dropped to 19% (95% CI, 6.5%-30%; *Q* = 21) and full vaccination to 43% (95% CI, 28%-55%; *Q* = 43). The pooled difference for children younger than 3 years was 26 pp (95% CI, 7.6 pp to 43 pp; *Q* = 21) (eFigure 12 in Supplement 1).

### Bias Assessment

Most trials (10 trials) included in the vaccine efficacy meta-analysis were rated as having a low risk of bias (eFigure 14 in Supplement 1). For the observational studies, 7 of the naive studies in the vaccine effectiveness meta-analysis were rated as having a moderate risk of bias and 7 as having a high risk of bias. The main sources of bias for these studies were potential confounding, bias in the selection of participants, and missing data bias (eFigures 15 through 17 in Supplement 1). One naive study was judged to have a critical risk of bias due to potential uncontrolled confounding (eFigure 15 in Supplement 1). All mixed-history studies were judged to have a critical risk of bias from misclassification of interventions (eFigure 17 in Supplement 1).

The Egger test suggested the presence of publication bias for IIV against any influenza for 2-dose efficacy (*P* < .001) and for effectiveness of 2 doses for influenza A (*P* = .01). Examination of the funnel plots supports this finding (eAppendix 5, eTable 4, and eFigures 19 and 21 in Supplement 1).

## Discussion

This systematic review of 51 studies of influenza vaccine efficacy and VE for influenza vaccine–naive children has found the second dose of IIV was not associated with a statistically significant increase in VE in the first year of vaccination for children younger than 9 years. However, it was associated with a statistically significant increase of 28 pp for those under 3 years old. These results suggest a 2-dose schedule may be protective for very young children, who are less likely to have been previously exposed to influenza, but that this benefit may not^5,8,41^ extend to 9 years of age. This assessment is supported by the lower pooled VE for 1-dose IIV effectiveness for studies of children younger than 3 years compared with studies of children younger than 9 years (14% and 35%, respectively). There are insufficient data to determine a more suitable age window.

There were no RCTs that estimated 1-dose IIV efficacy in influenza vaccine–naive children. Two-dose vaccine efficacy for IIV was 52% while 2-dose VE was 43% for children younger than 9 years. VE is typically lower than vaccine efficacy because RCTs (from which vaccine efficacy is estimated) tend to limit recruitment to healthy individuals while observational studies (from which VE is estimated) measure the protection outcomes offered by a vaccine, including in individuals whom may have medical conditions that blunt protection.^70^ We would expect that, had vaccine efficacy of 1 dose been measured, it would also have been higher than the corresponding estimates for 1-dose VE, and similarly demonstrate that the 2-dose schedule was preferable for influenza vaccine–naive children younger than 3 years.

Insufficient estimates were available to assess the pooled change in efficacy of LAIV. Pooled 1-dose LAIV efficacy (51%) was comparable with pooled 2-dose IIV efficacy and 2-dose LAIV efficacy was significantly higher (82%). However, pooled 1-dose LAIV VE (26%) was comparable with 1-dose IIV VE (35%). Therefore, the difference in efficacy between LAIV and IIV may be a function of the seasonal variation in antigenic similarity between vaccine and virus rather than improved protection.

Most of the direct comparisons identified in this review (23 of 28 studies) defined children as partially or fully vaccinated rather than by the number of doses received during the season. We classified these studies as mixed-history studies. The pooled estimates for partially and fully vaccinated children from these studies (32% for partial vaccination and 43% for full vaccination) are comparable with the results of Kalligeros et al’s systematic review^9^ of hospitalized influenza VE from TND studies (33.9% for partial and 61.8% for full vaccination).

The mixed-history studies were analyzed separately because they are vulnerable to differential and dependent exposure misclassification bias (eAppendix 4 in Supplement 1). This source of bias occurs because these studies make 2 assumptions. The first assumption is that 2 doses in the first year is the same as being fully vaccinated. However, the definition for fully vaccinated articulated in these studies included children vaccinated in prior years and thus could not validly test this assumption. The second assumption that may be source of study bias is that children who did not receive an influenza vaccination in the current year have never received an influenza vaccine. Longitudinal studies have found that between 15% and 50% of children vaccinated against influenza in one season will not be vaccinated in the subsequent season; therefore, influenza vaccine status in the current season may not be a reliable predictor of vaccine history.^71^ So despite influenza vaccination being a well-established public health intervention, few unbiased studies have been published that explore the benefit of a 2-dose schedule over and above a 1-dose schedule for influenza vaccine–naive children.

Unlike the observational studies in this review, all RCTs included applied consistent restrictions on influenza vaccine history across all study groups. However, only 1 RCT reported how influenza vaccine–history was ascertained. As sensitivity and specificity of vaccination history can vary significantly by source and within sources by location^72,73^ and may contribute to the heterogeneity of the estimates, this information should be routinely provided.

### Limitations

The overarching limitation of this systematic review and meta-analysis was the small number of available estimates and the high levels of uncertainty for those estimates resulting from small sample sizes. The limited number of available estimates meant there that were insufficient data to stratify the analysis and assess whether the incremental impact of the second influenza dose changed by disease severity, study design, source of vaccination history, or season. It was also not possible to assess the impact of 2 doses in children aged between 3 and 9 years separately from the broader age group because there were insufficient estimates. Furthermore, the pooled 1 and 2 dose estimates did not control for interseasonal variation including influenza vaccine mismatch. The use of directly comparable 1 and 2 dose estimates negated the impact of these interseasonal differences, but came at the cost of fewer observations. This meta-analysis has not assessed how the different approaches to adjustment may impact the comparability of vaccine efficacy or vaccine effectiveness estimates.

## Conclusions

In this systematic review and meta-analysis of influenza vaccine efficacy and effectiveness in the first year of vaccination, receiving 2 doses of IIV in the first year of influenza vaccination was associated with improved protection for children younger than 3 years compared with those who received 1 dose. When the age range was broadened to younger than 9 years the available evidence did not demonstrate a statistically significant increase in protection for children who receive 2 doses in the first year of vaccination with IIV. Insufficient estimates were available to assess the incremental benefit associated with a second dose of LAIV. We found that few RCTs have investigated the level of protection offered by both 1 and 2 doses in the same season or seasons and population and many of the observational studies identified were at a critical risk of bias from misclassification. Additional high-quality RCTs and observational studies with sufficient sample size and valid intervention definitions are needed to support a more definitive assessment of the impact of the recommended 2-dose schedule above a 1-dose schedule by age and disease severity for current influenza vaccines.

## Notes

Supplement 1. eTable 1. Search Strategy eFigure 1. Full-Text Decision Tree eAppendix 1. Data Extraction Form eAppendix 2. Additional Methodological Detail eFigure 2. Study Selection eFigure 3. Pooled IIV Vaccine Efficacy Estimates for Any Influenza for Children <9 Years Who Have Been Vaccinated for Influenza for the first Time in the Current Season With 2 Doses eFigure 4. Pooled IIV Vaccine Efficacy Estimates for Any Influenza for Children <3 Years Who Have Been Vaccinated for Influenza for the First Time in the Current Season With 2 Doses eFigure 5. Pooled IIV Vaccine Efficacy Estimates for Children <3 Years Who Have Been Vaccinated for Influenza for the First Time in the Current Season With 2 Doses for Influenza A and Influenza A Subtypes eFigure 6. Pooled IIV Vaccine Efficacy Estimates for Children <3 Years Who Have Been Vaccinated for Influenza for the First Time in the Current Season With 2 Doses for Influenza B and Influenza B Lineages eFigure 7. Pooled LAIV Vaccine Efficacy Estimates for Children <9 Years Who Have Been Vaccinated for Influenza for the First Time in the Current Season by the Number of Doses Received and the Difference Between These Estimates for H3N2 and Influenza B eFigure 8. Pooled IIV Vaccine Effectiveness Estimates for Children <3 Years Who Have Been Vaccinated for Influenza for the First Time in the Current Season by the Number of Doses Received and the Difference Between These Estimates eFigure 9. Pooled IIV Vaccine Effectiveness Estimates for Children <3 Years Who Have Been Vaccinated for Influenza for the First Time in the Current Season by the Number of Doses Received and the Difference Between These Estimates eFigure 10. Pooled LAIV Vaccine Effectiveness Estimates for Any Influenza for Children <9 Years Who Have Been Vaccinated for Influenza for the first Time in the Current Season With 1 Dose eFigure 11. Pooled IIV VE Estimates Against Influenza for Partially and Fully Vaccinated Children <9 Years and the Difference Between These Estimates eFigure 12. Pooled IIV VE Estimates Against Influenza for Partially and Fully Vaccinated Children <3 Years and the Difference Between These Estimates eAppendix 3. Monovalent A(H1N1)pdm09 Vaccine Subanalysis eFigure 13. Adjuvanted and Nonadjuvanted Monovalent IIV VE Estimates Against A(H1N1)pdm09 Influenza for Children <9 Years Who Have Received 1 Dose of Vaccine for the First Time in 2009/10 eTable 2. Comparison of Fixed Effect Model Pooled Estimates With Random Effect Model Pooled Estimates for Vaccine Efficacy Studies eTable 3. Comparison of Fixed Effect Model Pooled Estimates With Random Effect Model Pooled Estimates for Vaccine Effectiveness Studies eFigure 14. Risk of Bias Assessment for Included RCTs eFigure 15. Risk of Bias Assessment for Included Vaccine Effectiveness Studies Classified as Naive Studies eFigure 16. Risk of Bias Assessment for Included Vaccine Effectiveness Studies Classified as Monovalent A(H1N1)pdm09 Studies eFigure 17. Risk of Bias Assessment for Included Vaccine Effectiveness Studies Classified as Mixed History Studies eAppendix 4. Classification of Information Bias in Mixed-History Studies eFigure 18. Graphical Depiction of Differential and Dependent Misclassification Occurring in the Mixed-History Studies eAppendix 5. Publication Bias eFigure 19. Funnel Plots for Each Pooled Analysis for Any Influenza by Age Group and by Subtype for Children <3 Years Old for 2-Dose IIV RCTs Using a Previously Vaccine Naive Population eFigure 20. Funnel Plot for Pooled Analysis for Any Influenza for Children <3 Years Old for 1-Dose LAIV RCTs Using a Previously Vaccine Naive Population eTable 4. Egger Test for Publication Bias eFigure 21. Funnel Plots for Each Pooled Analysis by Influenza Type, Age Group and Number of Doses for IIV VE for Studies Using a Previously Vaccine Naive Population eFigure 22. Funnel Plots for Each Pooled Analysis by Influenza Type and Age Group for IIV VE for Studies Vulnerable to Confounding as a Result of Not Controlling for Vaccine History in Study Arms Other Than the Children Vaccinated for the First Time Who Only Received 1 Dose eTable 5. Studies Excluded at Full Text Screening Stage by Reason eTable 6. PRISMA 2020 Checklist eReferences.

Supplement 2. Data Sharing Statement
