# Error in Byline

**DOI:** 10.1001/jamanetworkopen.2025.46151

**Published:** 2025-10-31

In the Original Investigation titled “Comparison of 2 Doses vs 1 Dose in the First Season Children Are Vaccinated Against Influenza: A Systematic Review and Meta-Analysis,”^1^ published October 3, 2025, Katherine B. Gibney was omitted from the byline. This article has been corrected.^1^
