# Supplementary material for: Comparison of 2 Doses vs 1 Dose in the First Season Children Are Vaccinated Against Influenza: A Systematic Review and Meta-Analysis
Source: JAMA Netw Open. 2025 Oct 3;8(10):e2535250. doi: 10.1001/jamanetworkopen.2025.35250 (PMC12495502; doi:10.1001/jamanetworkopen.2025.35250)
Supplement: Supplement 1. — eTable 1. Search Strategy eFigure 1. Full-Text Decision Tree eAppendix 1. Data Extraction Form eAppendix 2. Additional Methodological Detail eFigure 2. Study Selection eFigure 3. Pooled IIV Vaccine Efficacy Estimates for Any Influenza for Children <9 Years Who Have Been Vaccinated for Influenza for the first Time in the Current Season With 2 Doses eFigure 4. Pooled IIV Vaccine Efficacy Estimates for Any Influenza for Children <3 Years Who Have Been Vaccinated for Influenza for the First Time in the Current Season With 2 Doses eFigure 5. Pooled IIV Vaccine Efficacy Estimates for Children <3 Years Who Have Been Vaccinated for Influenza for the First Time in the Current Season With 2 Doses for Influenza A and Influenza A Subtypes eFigure 6. Pooled IIV Vaccine Efficacy Estimates for Children <3 Years Who Have Been Vaccinated for Influenza for the First Time in the Current Season With 2 Doses for Influenza B and Influenza B Lineages eFigure 7. Pooled LAIV Vaccine Efficacy Estimates for Children <9 Years Who Have Been Vaccinated for Influenza for the First Time in the Current Season by the Number of Doses Received and the Difference Between These Estimates for H3N2 and Influenza B eFigure 8. Pooled IIV Vaccine Effectiveness Estimates for Children <3 Years Who Have Been Vaccinated for Influenza for the First Time in the Current Season by the Number of Doses Received and the Difference Between These Estimates eFigure 9. Pooled IIV Vaccine Effectiveness Estimates for Children <3 Years Who Have Been Vaccinated for Influenza for the First Time in the Current Season by the Number of Doses Received and the Difference Between These Estimates eFigure 10. Pooled LAIV Vaccine Effectiveness Estimates for Any Influenza for Children <9 Years Who Have Been Vaccinated for Influenza for the first Time in the Current Season With 1 Dose eFigure 11. Pooled IIV VE Estimates Against Influenza for Partially and Fully Vaccinated Children <9 Years and the Difference Between These Estimates eFigur [file jamanetwopen-e2535250-s001.pdf]

Supplemental Online Content

Goldsmith JJ, Tavlian S, Vu C, Regan AK, Campbell PT, Sullivan SG. Comparison of 2 doses vs 1 dose in the first season children are vaccinated against influenza: a systematic review and meta-analysis. *JAMA Netw Open*. 2025;8(10):e2535250. doi:10.1001/jamanetworkopen.2025.35250

eTable 1. Search Strategy

eFigure 1. Full-Text Decision Tree

eAppendix 1. Data Extraction Form

eAppendix 2. Additional Methodological Detail

eFigure 2. Study Selection

eFigure 3. Pooled IIV Vaccine Efficacy Estimates for Any Influenza for Children <9 Years Who Have Been Vaccinated for Influenza for the first Time in the Current Season With 2 Doses

eFigure 4. Pooled IIV Vaccine Efficacy Estimates for Any Influenza for Children <3 Years Who Have Been Vaccinated for Influenza for the First Time in the Current Season With 2 Doses

eFigure 5. Pooled IIV Vaccine Efficacy Estimates for Children <3 Years Who Have Been Vaccinated for Influenza for the First Time in the Current Season With 2 Doses for Influenza A and Influenza A Subtypes

eFigure 6. Pooled IIV Vaccine Efficacy Estimates for Children <3 Years Who Have Been Vaccinated for Influenza for the First Time in the Current Season With 2 Doses for Influenza B and Influenza B Lineages

eFigure 7. Pooled LAIV Vaccine Efficacy Estimates for Children <9 Years Who Have Been Vaccinated for Influenza for the First Time in the Current Season by the Number of Doses Received and the Difference Between These Estimates for H3N2 and Influenza B

eFigure 8. Pooled IIV Vaccine Effectiveness Estimates for Children <3 Years Who Have Been Vaccinated for Influenza for the First Time in the Current Season by the Number of Doses Received and the Difference Between These Estimates

eFigure 9. Pooled IIV Vaccine Effectiveness Estimates for Children <3 Years Who Have Been Vaccinated for Influenza for the First Time in the Current Season by the Number of Doses Received and the Difference Between These Estimates

eFigure 10. Pooled LAIV Vaccine Effectiveness Estimates for Any Influenza for Children <9 Years Who Have Been Vaccinated for Influenza for the first Time in the Current Season With 1 Dose

eFigure 11. Pooled IIV VE Estimates Against Influenza for Partially and Fully Vaccinated Children <9 Years and the Difference Between These Estimates

eFigure 12. Pooled IIV VE Estimates Against Influenza for Partially and Fully Vaccinated Children <3 Years and the Difference Between These Estimates

eAppendix 3. Monovalent A(H1N1)pdm09 Vaccine Subanalysis

eFigure 13. Adjuvanted and Nonadjuvanted Monovalent IIV VE Estimates Against A(H1N1)pdm09 Influenza for Children <9 Years Who Have Received 1 Dose of Vaccine for the First Time in 2009/10

eTable 2. Comparison of Fixed Effect Model Pooled Estimates With Random Effect Model Pooled Estimates for Vaccine Efficacy Studies

eTable 3. Comparison of Fixed Effect Model Pooled Estimates With Random Effect Model Pooled Estimates for Vaccine Effectiveness Studies

eFigure 14. Risk of Bias Assessment for Included RCTs eFigure 15. Risk of Bias Assessment for Included Vaccine Effectiveness Studies Classified as Naïve Studies

eFigure 16. Risk of Bias Assessment for Included Vaccine Effectiveness Studies Classified as Monovalent A(H1N1)pdm09 Studies

eFigure 17. Risk of Bias Assessment for Included Vaccine Effectiveness Studies Classified as Mixed History Studies

eAppendix 4. Classification of Information Bias in Mixed-History Studies

eFigure 18. Graphical Depiction of Differential and Dependent Misclassification Occurring in the Mixed-History Studies

eAppendix 5. Publication Bias

eFigure 19. Funnel Plots for Each Pooled Analysis for Any Influenza by Age Group and by Subtype for Children <3 Years Old for 2-Dose IIV RCTs Using a Previously Vaccine Naive Population

eFigure 20. Funnel Plot for Pooled Analysis for Any Influenza for Children <3 Years Old for 1-Dose LAIV RCTs Using a Previously Vaccine Naive Population

eFigure 22. Funnel Plots for Each Pooled Analysis by Influenza Type and Age Group for IIV VE for Studies Vulnerable to Confounding as a Result of Not Controlling for Vaccine History in Study Arms Other Than the Children Vaccinated for the First Time Who Only Received 1 Dose

eTable 4. Egger Test for Publication Bias eFigure 21. Funnel Plots for Each Pooled Analysis by Influenza Type, Age Group and Number of Doses for IIV VE for Studies Using a Previously Vaccine Naive Population

eTable 5. Studies Excluded at Full Text Screening Stage by Reason

eTable 6. PRISMA 2020 Checklist

eReferences

This supplemental material has been provided by the authors to give readers additional information about their work.

eTable 1. Search strategy

Domain	Search terms	Fields
Influenza	Influenza or flu	Title
Vaccination effectiveness or efficacy	((efficacy or effectiveness) within 6 words of (vaccin* or immuni$e or immuni$ation* or protect*))	Keyword
Children	(child* or juvenil* or kindergarten* or minor? or paediatric* or peadiatric* or pediatric* or PICU or preschool* or pre-school* or toddler? or young or youngster* or youth* or school-age* or schoolage or infant* or infancy or baby or babies)	Keyword

Notes: : "adj" is the proximity operator used by EMBASE and Ovid MEDLINE databases. CINAHL uses "w".

eFigure 1. Full-text decision tree

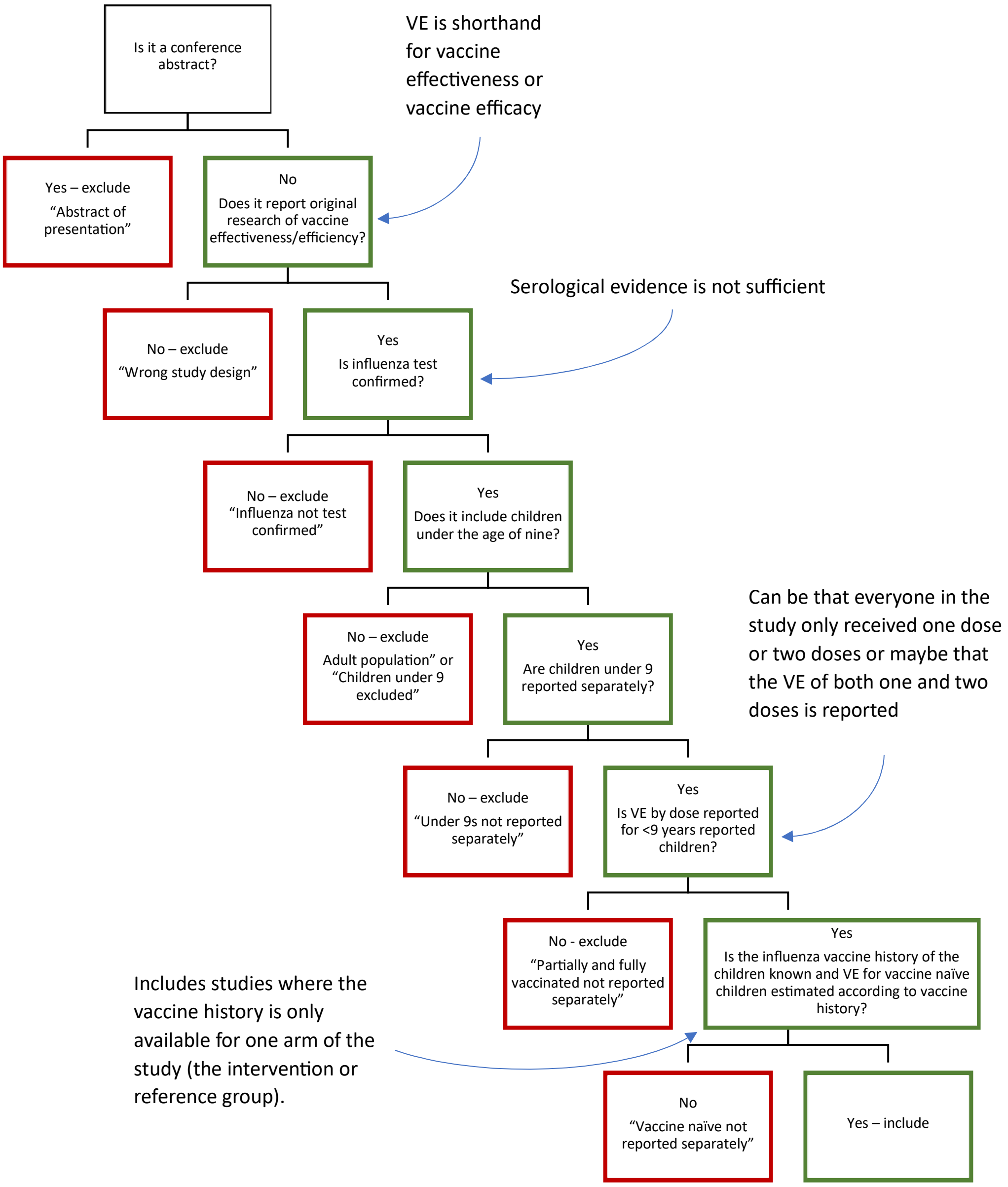

eAppendix 1. Data extraction form

1. Contact author’s name ______________________
2. Contact author’s email ______________________
3. Supplementary material
   - ☐ Yes
   - ☐ No
4. Errata
   - ☐ Yes
   - ☐ No
5. Publication language
   - ☐ English
   - ☐ Other ______________________
6. Study design
   - ☐ Cohort (prospective)
   - ☐ Cohort (retrospective)
   - ☐ Case-control
   - ☐ Case-control (case-case)
   - ☐ Case-control (test-negative design)
   - ☐ Screening
   - ☐ Randomized control trial
   - ☐ Other ______________________
7. Effect measure
   - ☐ Odds ratio
   - ☐ Risk ratio
   - ☐ Hazard ratio
   - ☐ Other ______________________
8. Hemisphere
   - ☐ Northern
   - ☐ Southern
   - ☐ Both
9. Country
   - ☐ UK
   - ☐ USA
   - ☐ Japan

☐ Australia

☐ China

☐ China (HK)

☐ Other ______________________________

10. Settings

☐ GP clinics

☐ Emergency departments

☐ Hospitals

☐ Other ______________________________

11. Source of influenza vaccination history

☐ Parent/guardian

☐ Medical practitioner

☐ Health record

☐ Immunization record

☐ Insurance company

☐ Unstated

☐ Other ______________________________

12. Vaccination definitions and history ______________________________

13. Measures of effect tables

Measures of effect table for RCTs

Characteristic	1st measure	Additional measures →
Year/s		
Number of seasons		
Type/subtype		
Age range		
Doses intervention (1, 1+, 2, 2+)		
Doses comparator (0, 1)		
Vaccine type		
VE (%)		
VE lower CI (95%)		
VE upper CI (95%)		
Analysis type (PP/ITT)		
Intervention arm (infected(n)/n)		
Control arm (infected(n)/n)		
Vaccine history of intervention arm (N,1,1+)		
Vaccine history of control arm (N,1,1+)		

Measures of effect table for observational studies

Characteristic	1st measure	Additional measures →
Year/s		
Number of seasons		
Type/subtype		
Age range		
Doses intervention (1, 1+, 2, 2+)		
Doses comparator (0, 1)		
Vaccine type		
Crude VE (%)		
Crude VE lower CI (95%)		
Crude VE upper CI (95%)		
Adjusted VE (%)		
Adjusted VE lower CI (95%)		
Adjusted VE upper CI (95%)		
Adjustment variables		
Cases (vaccinated(n)/n) or vaccinated (infected(n)/n)		
Controls (vaccinated(n)/n) or comparison(infected(n)/n)		
Vaccine history of cases or vaccinated (N,1,1+)		
Vaccine history of controls or unvaccinated (N,1,1+)		

14. Hand searched papers ______________________________

15. Notes ______________________________

eAppendix 2. Additional methodological detail

Code uses Metafor, Epitools and Tidyverse packages. ".c" is used to denote functions for two dose or full vaccination estimates and ".b" for functions for one dose or partial vaccination estimates. Much of this code has been adapted from R code published by Jones-Gray and colleagues[1].

Functions to convert VE to OR coefficients - needed for the rma models.

```
logscale <- function(x){ # x is the original dataframe
    logscale.m <- dfn %>%
    mutate(coef = log((100-ve)/100,base=exp(1)),
           coef.lb = log((100-ll)/100,base=exp(1)),
           coef.ub = log((100-ul)/100,base=exp(1)))
  return(logscale.m)
}

logscale.c <-function(x){
  logscale.m <-x
  logscale.m$full.or <- log((100-x$ve_full)/100,base=exp(1))
  logscale.m$full.or.lb <- log((100-x$ul_full)/100,base=exp(1))
  logscale.m$full.or.ub <- log((100-x\ll_full)/100,base=exp(1))
  return(logscale.m)
  }


logscale.b <-function(x){
  logscale.o <- x
  logscale.o$part.or <- log((100-x$ve_part)/100,base=exp(1))
  logscale.o$part.or.lb <- log((100-x$ul_part)/100,base=exp(1))
  logscale.o$part.or.ub <- log((100-x$ll_part)/100,base=exp(1))
  return(logscale.o)
}
```

Functions to convert confidence intervals to standard error.

```
stderror <- function(x){
  se <- x %>%
    mutate(se = (coef.ub - coef.lb)/3.92,
           V = se^2)
  return(se)
}

stderror.c <- function(x) {
  c.se <- x
  c.se$full.se <- (c.se$full.or.ub-c.se$full.or.lb)/3.92
  return(c.se)
}

stderror.b <- function(x){
  b.se <- x
  b.se$part.se <- (b.se$part.or.ub-b.se$part.or.lb)/3.92
  return(b.se)}
```

Function to convert summary statistic from rma models back to VE scale.

```
changescale <- function(res){
  res.c <- res
  res.c$b <- (1-exp(res$b))*100
  res.c$beta <- (1-exp(res$b))*100
```

```
  res.c$ci.lb <- (1-exp(res$ci.ub))*100
  res.c$ci.ub <- (1-exp(res$ci.lb))*100
  return(res.c)
}
```

Function for pooled estimates.

```
calc.ma.model <- function(dfn.mod){
  models <- list()
  models$remodel <- changescale(rma(yi=dfn.mod$coef, sei=dfn.mod$se,
                                     data=dfn.mod, measure="OR",
                                    slab=dfn.mod$study_id, method="DL"))
  models$femodel <- changescale(rma(yi=dfn.mod$coef, sei=dfn.mod$se,
                                    data=dfn.mod, measure="OR",
                                    slab=dfn.mod$study_id, method="FE"))
  return(models)
}
```

Functions that combines the above functions for pooled estimates by dose or full and partial vaccination.

```
get.ma <- function(x){
  ma.out <- x %>%
    logscale() %>%
    stderror() %>%
    calc.ma.model()
  return(ma.out)
}
```

Function for pooled difference between two and one doses or full and partial vaccination.

```
calc.ma.model.delta<-function(dfn.mod){
  models <- list()
  models$remodel <- rma(yi=dfn.mod$meta.delta.ve.mean,
                        sei=dfn.mod$meta.delta.ve.se,
                        data = dfn.mod, method="DL")
  models$femodel <- rma(yi=dfn.mod$meta.delta.ve.mean,
                        sei=dfn.mod$meta.delta.ve.se,
                        data = dfn.mod, method="FE")
  return(models)
}
```

Function for bootstrapping confidence intervals for the delta VE estimates

```
get_delta_cis<-function(x, seed, n){

  delta_ve_dat<-x %>%
    mutate(both = as.numeric(onedose) +as.numeric(twodose)) %>%
    filter(both ==2)%>%
    pivot_wider(id_cols = c(study_id, study_design, setting, year,
                            type,  country, overall_flag, age),
                            names_from = vaccine_status,
                            values_from = c(ve,ll,ul, cases_vaccinated,
                                            cons_vaccinated)) %>%
    filter(is.na(ve_full)==FALSE) %>%
    filter(is.na(ve_part)==FALSE) %>%
    mutate(delta_ve = ve_full - ve_part)

  ci.dat <- delta_ve_dat
  ci.dat <- logscale.c(ci.dat)
  ci.dat <- logscale.b(ci.dat)
  ci.dat<- stderror.c(ci.dat)
  ci.dat<- stderror.b(ci.dat)

  set.seed (seed)
  n.sample <- n

  delta.ve.ll <- delta.ve.ul <- rep(NA, n.sample)
  # calculate mean and SE for each delta VE for meta - analysis
  meta.delta.ve.mean <- meta.delta.ve.se <- rep(NA, n.sample)
  # Bootstrap
  for (i in 1 : nrow (ci.dat)){
    for(j in 1:n.sample){
      ve_full <- (1-exp(rnorm(1000,ci.dat$full.or[i],ci.dat$full.se[i])))*100
      ve_part < -(1-exp(rnorm(1000,ci.dat$part.or[i],ci.dat$part.se[i])))*100
      delta.ve.ll[j] <- quantile(ve_full-ve_part,0.025,na.rm=TRUE)
      delta.ve.ul[j] <- quantile(ve_full-ve_part,0.975,na.rm=TRUE)
      meta.delta.ve.mean[j] <- mean(ve_full-ve_part)
      meta.delta.ve.se[j] <- sd(ve_full-ve_part)}

    ci.dat$delta.ve.ll[i] <- round(mean(delta.ve.ll),0)
    ci.dat$delta.ve.ul[i] <- round(mean(delta.ve.ul),0)
    ci.dat$meta.delta.ve.mean[i] <- round(mean(meta.delta.ve.mean),0)
    ci.dat$meta.delta.ve.se[i] <- round(mean(meta.delta.ve.se),3)
  } #end of loop

  return(ci.dat)

}
```

eFigure 2. Study selection

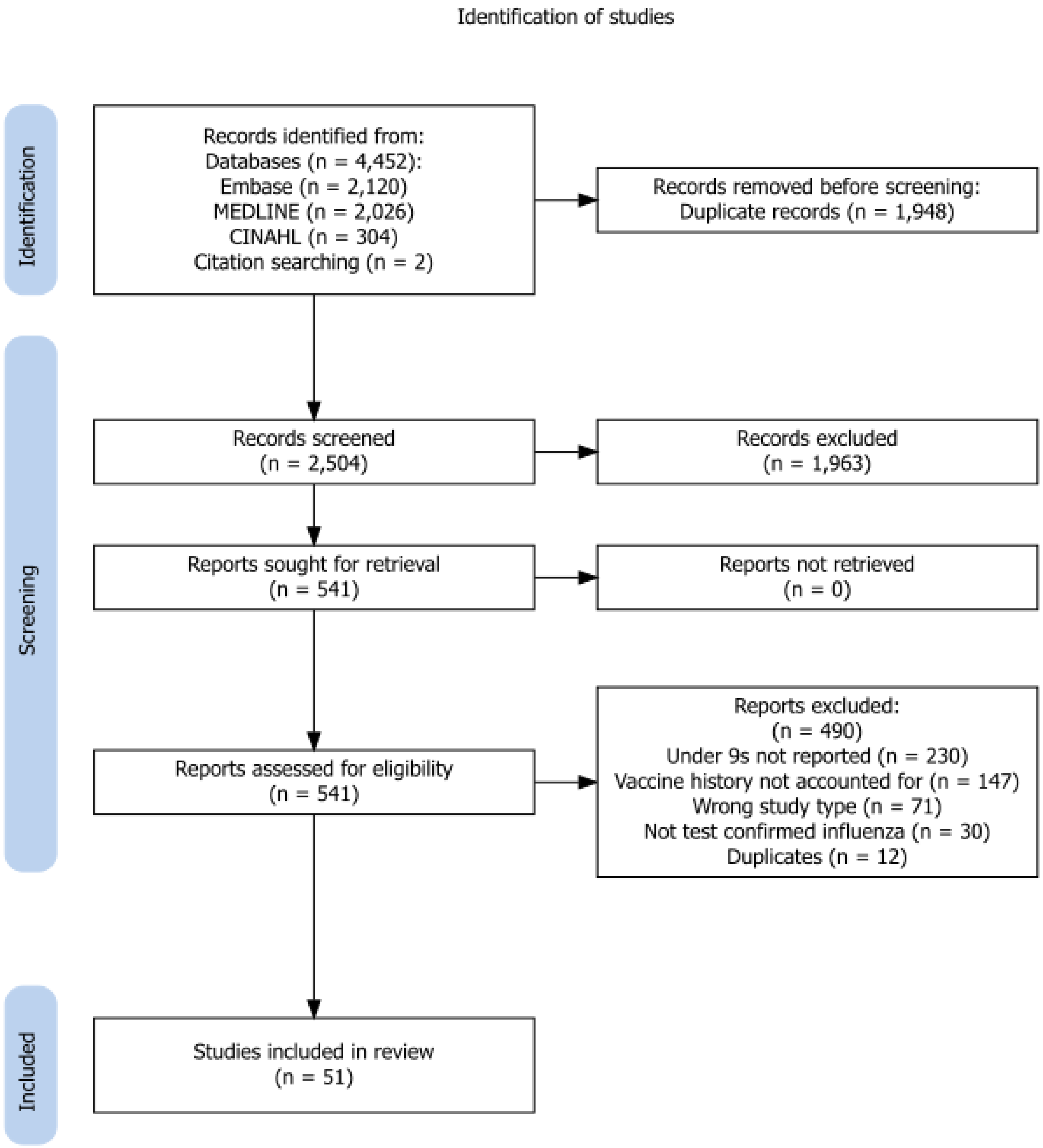

eFigure 3. Pooled IIV vaccine efficacy estimates for any influenza for children <9 years who have been vaccinated for influenza for the first time in the current season with two doses. The reference group is children <9 years who have never been vaccinated for influenza.

Two doses

Study	Age	Years	Vaccine-naïve n/N	2-doses n/N	Efficacy (95% CI)
Esposito	0.5-<3	2019	NR	NR	42 (-60–79)
Esposito	0.5-<3	2017/18-18/19	NR	NR	73 (50–85)
Pepin	0.5-<3	2015/16	62/335	26/344	59 (34–75)
Krishnan	5-<9	2015	91/269	28/478	72 (56–82)
Krishnan*	2-<5	2015	75/340	41/326	46 (22–62)
Pepin	0.5-<3	2015	44/491	19/488	57 (24–76)
Pepin	0.5-<3	2014/15	35/495	14/490	60 (23–80)
Dbaibo	0.5-<3	2014	274/2327	166/2323	41 (29–52)
Pepin	0.5-<3	2014	104/1170	61/1166	41 (19–58)
Dbaibo	0.5-<3	2013	98/750	71/751	30 (5.5–49)
Dbaibo	0.5-<3	2012/13	123/776	35/788	73 (62–82)
Dbaibo	0.5-<3	2012	81/1266	38/1260	53 (31–68)
Dbaibo	0.5-<3	2011/12	100/893	43/884	58 (40–71)
Rolfes*†	0.5-<2	2010/11-13/14	NR	NR	26 (9.2–40)
Vesikari*	0.5-<6	2007/08-08/09	47/993	50/1772	43 (15–61)
Random-effects model					52 (43–60)
Fixed-effects model					47 (42–51) (I²=68%, Q=44)

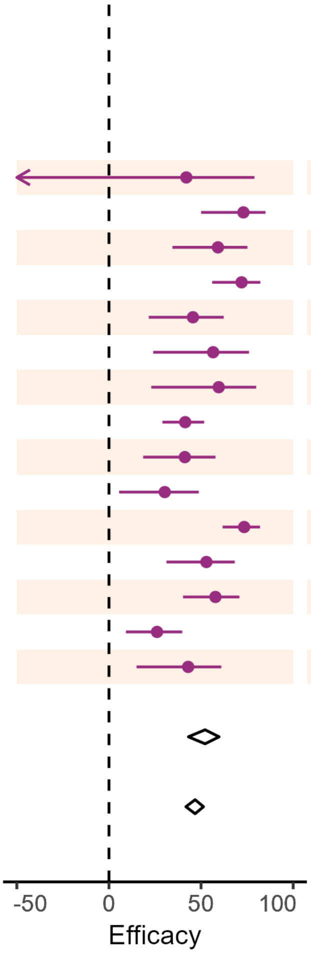

Note: *Children <3-years received 7.5µg per strain and vaccine dose of antigen. †44% of B influenza infections were of the lineage not included in the vaccine. Pooled IIV vaccine efficacy for two doses for matched influenza strains was higher [76% (95% CI: 69%–81%)]. Pooled IIV vaccine efficacy using a random effects model for two full doses (15 µg per strain and vaccine dose of antigen) was 56% (95% CI: 46%-64%).

eFigure 4. Pooled IIV vaccine efficacy estimates for any influenza for children <3 years who have been vaccinated for influenza for the first time in the current season with two doses. The reference group is children <3 years who have never been vaccinated for influenza.

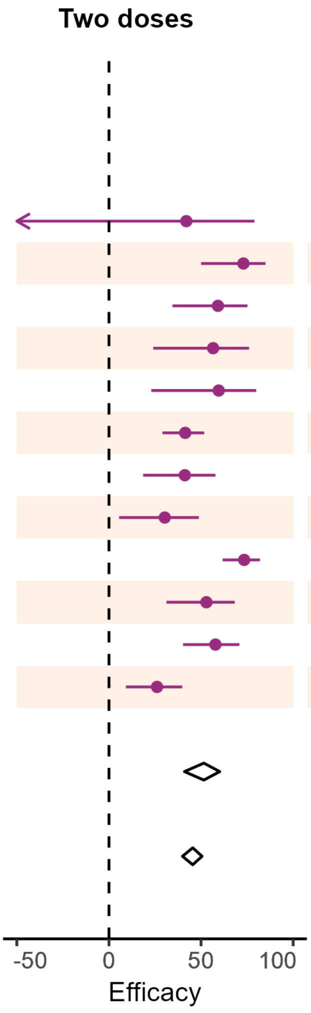

Study	Age	Years	Vaccine-naïve n/N	2-doses n/N	Efficacy (95% CI)
Esposito	0.5-<3	2019	NR	NR	42 (-60–79)
Esposito	0.5-<3	2017/18-18/19	NR	NR	73 (50–85)
Pepin	0.5-<3	2015/16	62/335	26/344	59 (34–75)
Pepin	0.5-<3	2015	44/491	19/488	57 (24–76)
Pepin	0.5-<3	2014/15	35/495	14/490	60 (23–80)
Dbaibo	0.5-<3	2014	274/2327	166/2323	41 (29–52)
Pepin	0.5-<3	2014	104/1170	61/1166	41 (19–58)
Dbaibo	0.5-<3	2013	98/750	71/751	30 (5.5–49)
Dbaibo	0.5-<3	2012/13	123/776	35/788	73 (62–82)
Dbaibo	0.5-<3	2012	81/1266	38/1260	53 (31–68)
Dbaibo	0.5-<3	2011/12	100/893	43/884	58 (40–71)
Rolfes*†	0.5-<2	2010/11-13/14	NR	NR	26 (9.2–40)
Random-effects model					51 (41–60)
Fixed-effects model					45 (40–51)
					(I^2=69%, Q=36)

Note: *Vaccine dose contained 7.5μg per strain of antigen. †44% of B influenza infections were of the lineage not included in the vaccine. Pooled IIV vaccine efficacy for two dose for matched influenza strains was higher [62% (95% CI: 54%–70%)]. Pooled IIV vaccine efficacy using a random effects model for two full doses (15 µg per strain and vaccine dose of antigen) was 54% (95% CI: 44%-62%).

eFigure 5. Pooled IIV vaccine efficacy estimates for children <3 years who have been vaccinated for influenza for the first time in the current season with two doses for influenza A and influenza A subtypes. The reference group is children <3 years who have never been vaccinated for influenza.

A

Study	Age	Years	Vaccine-naïve n/N	2-doses n/N	Efficacy (95% CI)
Esposito	0.5-<3	2017/18-19			
Pepin	0.5-<3	2014-15/16	147/2491	65/2489	56 (40–67)
Dbaibo	0.5-<3	2014			
Dbaibo	0.5-<3	2013			
Dbaibo	0.5-<3	2012/13			
Dbaibo	0.5-<3	2012			
Claeys	0.5-<3	2011/12-14	434/6012	221/6006	50 (42–58)
Dbaibo	0.5-<3	2011/12			
Random-effects model					
Fixed-effects model					

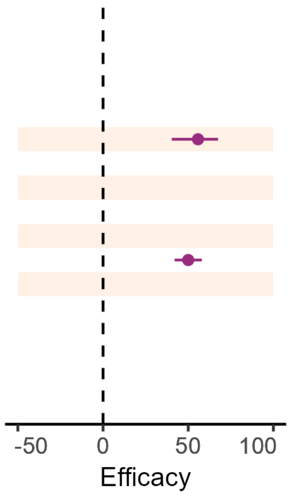

A(H1N1)pdm09

Study	Vaccine-naïve n/N	2-doses n/N	Efficacy (95% CI)
Esposito	45/995	21/1005	52 (16–72)
Pepin	44/2491	11/2489	75 (51–88)
Dbaibo	5/2327	4/2323	22 (-200–81)
Dbaibo	24/750	14/751	43 (-8.7–71)
Dbaibo	60/776	11/788	82 (68–91)
Dbaibo	12/1266	5/1260	58 (-14–86)
Claeys			
Dbaibo	2/893	1/884	50 (-420–98)
Random-effects model			63 (44–75)
Fixed-effects model			63 (51–73) (I²=39%, Q=10)

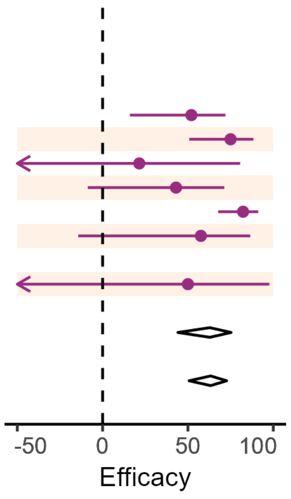

H3N2

Study	Age	Years	Vaccine-naïve n/N	2-doses n/N	Efficacy (95% CI)
Esposito	0.5-<3	2017/18-19	42/995	27/1005	34 (-10–60)
Pepin	0.5-<3	2014-15/16	103/2491	53/2489	48 (28–64)
Dbaibo	0.5-<3	2014	135/2327	88/2323	36 (16–51)
Dbaibo	0.5-<3	2013	60/750	38/751	38 (7.3–59)
Dbaibo	0.5-<3	2012/13	22/776	7/788	68 (30–88)
Dbaibo	0.5-<3	2012	21/1266	10/1260	51 (-0.7–78)
Claeys	0.5-<3	2011/12-14			
Dbaibo	0.5-<3	2011/12	93/893	40/884	58 (39–71)
Random-effects model					45 (36–53)
Fixed-effects model					45 (36–53) (I²=0%, Q=6)

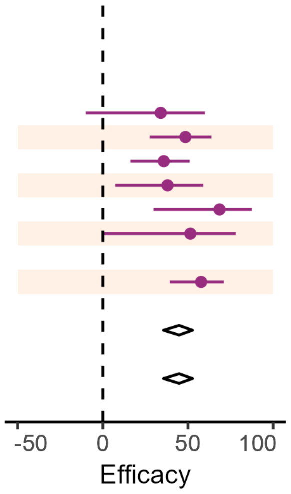

©2025 Goldsmith JJ et al. *JAMA Network Open.*

eFigure 6. Pooled IIV vaccine efficacy estimates for children <3 years who have been vaccinated for influenza for the first time in the current season with two doses for influenza B and influenza B lineages. The reference group is children <3 years who have never been vaccinated for influenza.

B

Study	Age	Years	Vaccine-naïve n/N	2-doses n/N	Efficacy (95% CI)
Esposito	0.5-<3	2017/18-19	37/995	15/1005	70 (40–84)
Pepin	0.5-<3	2014-15/16	106/2491	58/2489	45 (24–61)
Dbaibo	0.5-<3	2014			
Dbaibo	0.5-<3	2013			
Dbaibo	0.5-<3	2012/13			
Dbaibo	0.5-<3	2012			
Claeys	0.5-<3	2011/12-14	258/6012	139/6006	47 (35–57)
Random-effects model					50 (37–60)
Fixed-effects model					49 (39–57) (I²=28%, Q=3)

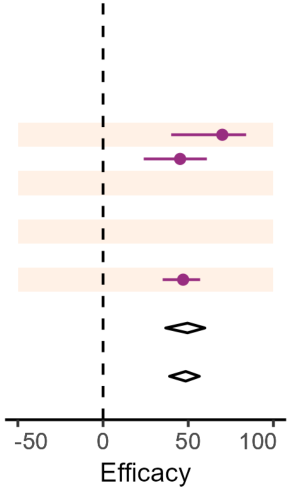

B (Vic.)

Study	Age	Years	Vaccine-naïve n/N	2-doses n/N	Efficacy (95% CI)
Esposito	0.5-<3	2017/18-19			
Pepin	0.5-<3	2014-15/16	20/2491	12/2489	40 (-29–73)
Dbaibo	0.5-<3	2014	9/2327	5/2323	45 (-58–83)
Dbaibo	0.5-<3	2013	16/750	19/751	-17 (-130–40)
Dbaibo	0.5-<3	2012/13	5/776	2/788	60 (-84–94)
Dbaibo	0.5-<3	2012	8/1266	3/1260	62 (-31–92)
Claeys	0.5-<3	2011/12-14			
Random-effects model					28 (-11–53)
Fixed-effects model					28 (-11–53) (I²=0%, Q=4)

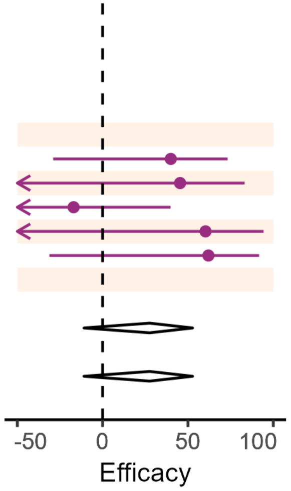

B (Yam.)

Study	Age	Years	Vaccine-naïve n/N	2-doses n/N	Efficacy (95% CI)
Esposito	0.5-<3	2017/18-19			
Pepin	0.5-<3	2014-15/16	63/2491	26/2489	59 (34–75)
Dbaibo	0.5-<3	2014	127/2327	69/2323	47 (29–61)
Dbaibo	0.5-<3	2013	1/750	1/751	1.9 (-2400–96)
Dbaibo	0.5-<3	2012/13	42/776	15/788	65 (38–81)
Dbaibo	0.5-<3	2012	37/1266	21/1260	42 (2.5–67)
Claeys	0.5-<3	2011/12-14			
Random-effects model					51 (40–61)
Fixed-effects model					51 (40–61) (I²=0%, Q=2)

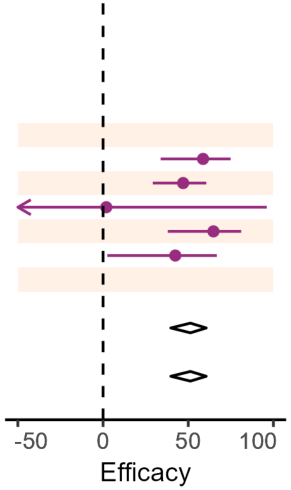

Note: All studies where IIV vaccine efficacy for influenza B and influenza B lineages were reported used quadrivalent vaccines.

©2025 Goldsmith JJ et al. *JAMA Network Open.*

©2025 Goldsmith JJ et al. *JAMA Network Open.*

eFigure 7. Pooled LAIV vaccine efficacy estimates for children <9 years who have been vaccinated for influenza for the first time in the current season by the number of doses received and the difference between these estimates for (A) H3N2 and (B) influenza B. The reference group is children <9 years who have never been vaccinated for influenza.

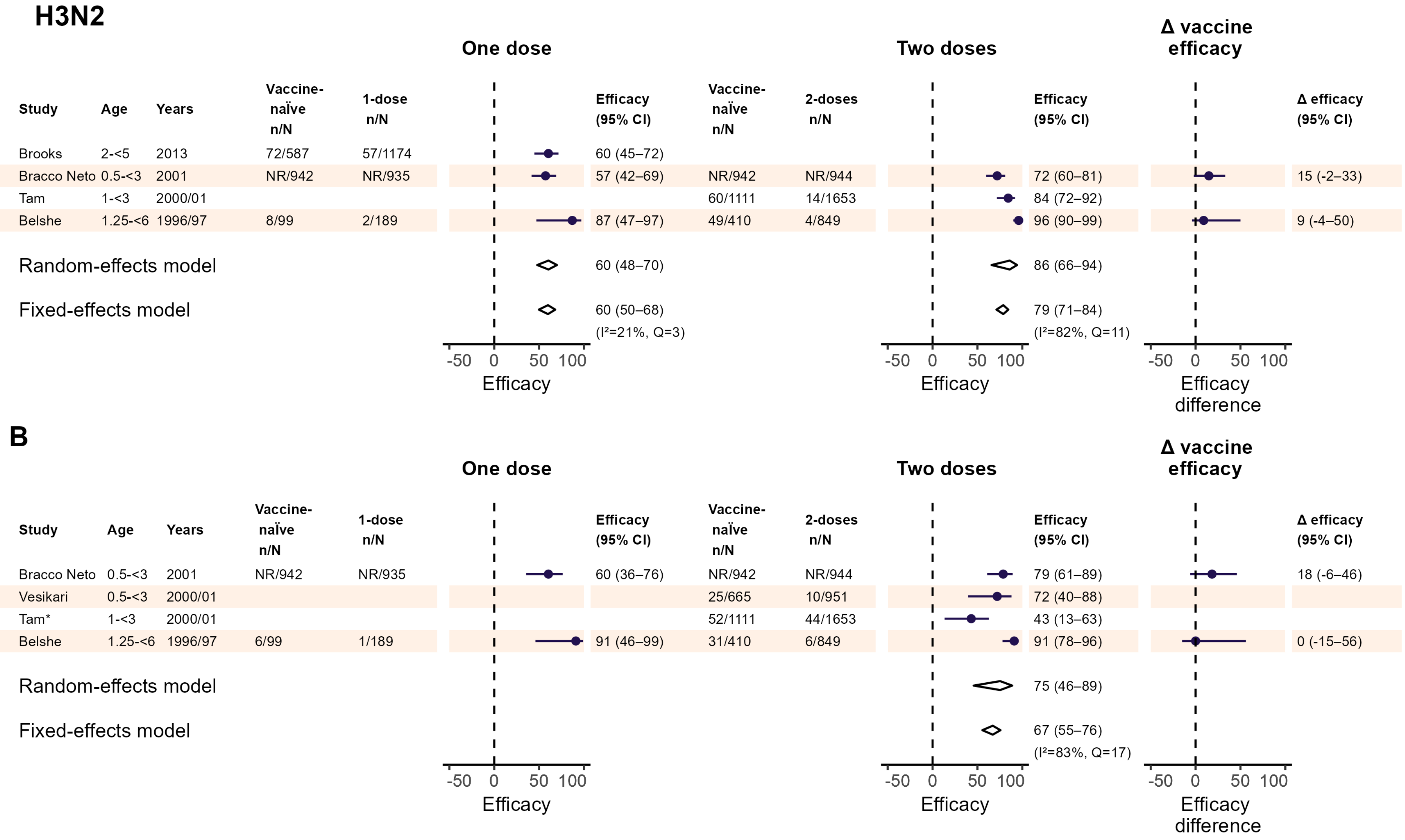

Note: $\Delta VE = VE_2 - VE_1$. $\Delta VE > 0$ indicates greater efficacy for two doses. *29% of B influenza was B/Vic. lineage and unmatched (not in the vaccine).

©2025 Goldsmith JJ et al. *JAMA Network Open.*

eFigure 8. Pooled IIV vaccine effectiveness estimates for children <3 years who have been vaccinated for influenza for the first time in the current season by the number of doses received and the difference between these estimates. The reference group is children <3 years who have never been vaccinated for influenza.

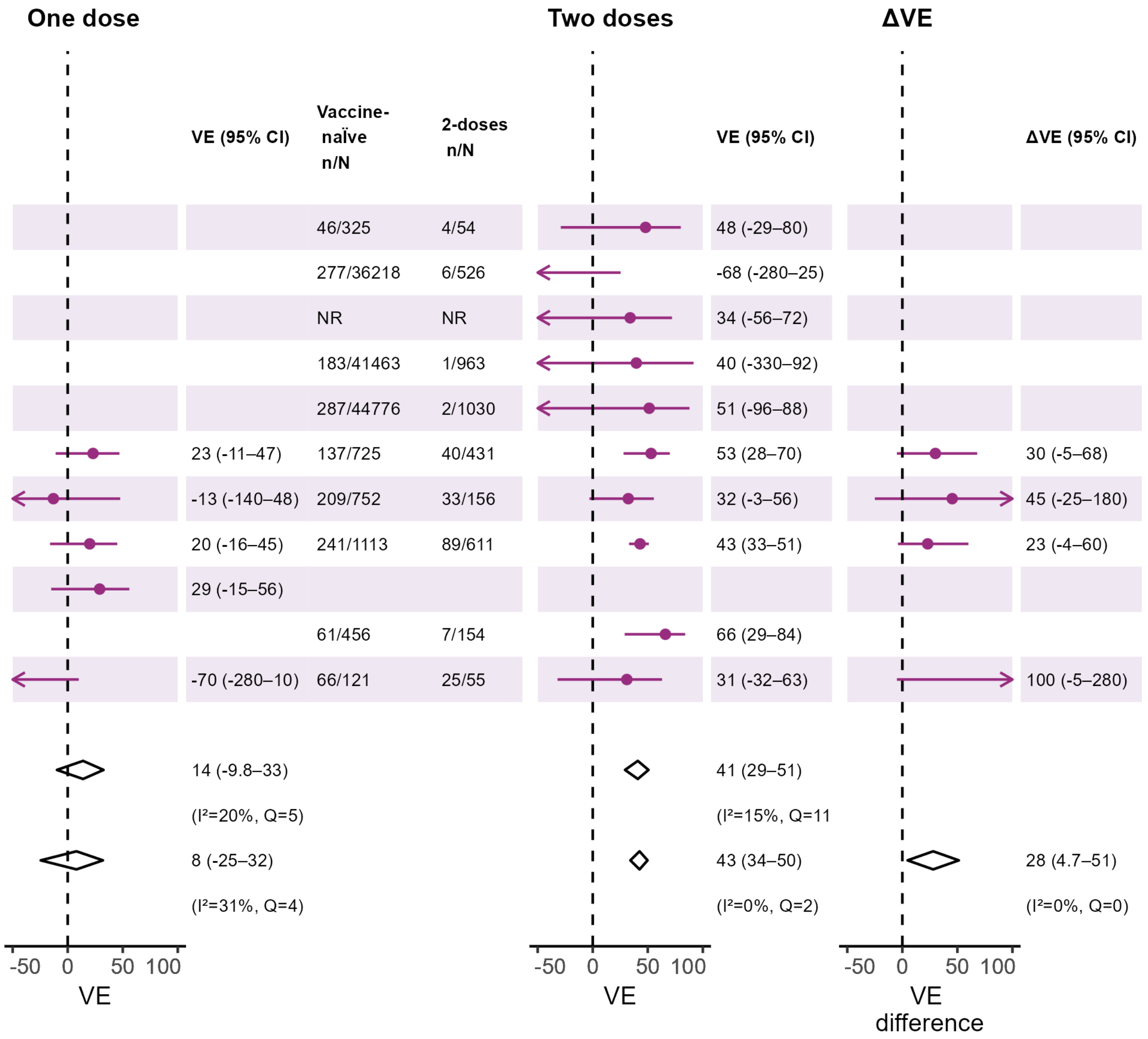

Study	Age	Years	Pop.	Vaccine-naïve n/N	1-dose n/N	VE (95% CI)	Vaccine-naïve n/N	2-doses n/N	VE (95% CI)	ΔVE (95% CI)
Mattila	0.5-<1	2017/18	OP				46/325	4/54	48 (-29–80)	
Baum	2-<3	2017/18	Reg.				277/36218	6/526	-68 (-280–25)	
Rao	0.5-<2	2016/17-17/18	OP				NR	NR	34 (-56–72)	
Baum	2-<3	2016/17	Reg.				183/41463	1/963	40 (-330–92)	
Baum	2-<3	2015/16	Reg.				287/44776	2/1030	51 (-96–88)	
Chung	0.5-<2	2014/15-17/18	OP	137/725	54/363	23 (-11–47)	137/725	40/431	53 (28–70)	30 (-5–68)
Shinjoh 2019	0.5-<1	2013/14-17/18	OP	209/752	10/36	-13 (-140–48)	209/752	33/156	32 (-3–56)	45 (-25–180)
Sofia Arriola	0.5-<2	2013-2017	IP	241/1113	83/445	20 (-16–45)	241/1113	89/611	43 (33–51)	23 (-4–60)
Wagner	0.5-<2	2012-2016	Study	NR	NR	29 (-15–56)				
Heinonen	0.75-<3	2007/08	Study				61/456	7/154	66 (29–84)	
Shuler	0.5-<2	2003/04	OP	27/82	25/55	-70 (-280–10)	66/121	25/55	31 (-32–63)	100 (-5–280)
Random-effects model, all estimates						14 (-9.8–33) (I²=20%, Q=5)			41 (29–51) (I²=15%, Q=11	
Random-effects model, ΔVE estimates						8 (-25–32) (I²=31%, Q=4)			43 (34–50) (I²=0%, Q=2)	28 (4.7–51) (I²=0%, Q=0)

$\Delta VE = VE_2 - VE_1$. $\Delta VE > 0$ indicates greater effectiveness for two doses. Pop. = population, IP = inpatient, OP = outpatient, Reg. = registry, Study = study cohort (e.g. trial participants).

©2025 Goldsmith JJ et al. *JAMA Network Open.*

eFigure 9. Pooled IIV vaccine effectiveness estimates for children <3 years who have been vaccinated for influenza for the first time in the current season by the number of doses received and the difference between these estimates for (A) influenza A and (B) influenza B. The reference group is children <3 years who have never been vaccinated for influenza.

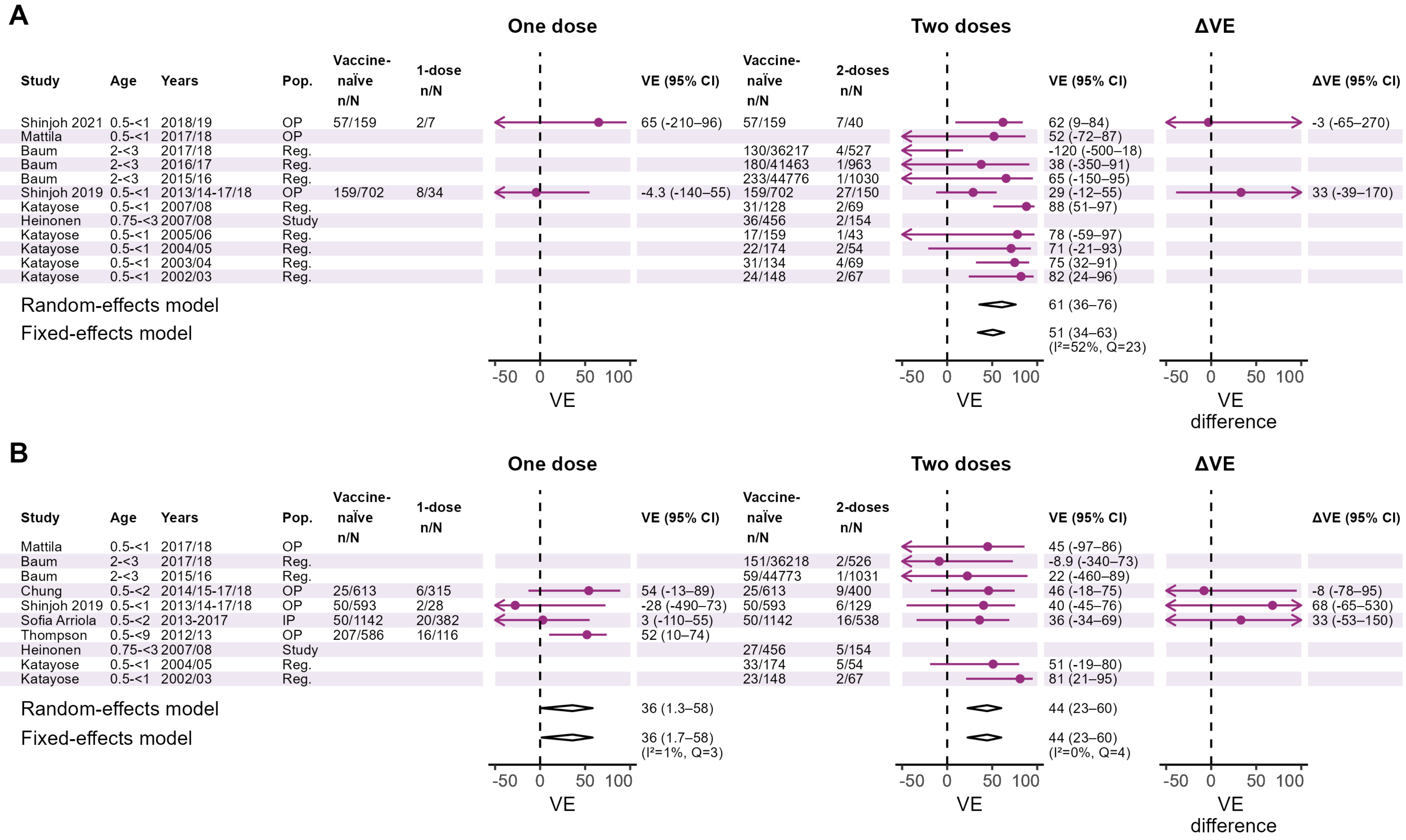

$\Delta VE = VE_2 - VE_1$. $\Delta VE > 0$ indicates greater effectiveness for two doses. Pop. = population, IP = inpatient, OP = outpatient, Reg. = registry, Study = study cohort (e.g. trial participants).

eFigure 10. Pooled LAIV vaccine effectiveness estimates for any influenza for children <9 years who have been vaccinated for influenza for the first time in the current season with one dose. The reference group is children <9 who have never been vaccinated for influenza.

Study	Age	Year	Pop.	Unvaccinated n/N	1-dose n/N	VE (95% CI)
Baum	2-<3	2017/18	Reg.	277/33923	21/3228	20 (-25–48)
Baum	2-<3	2016/17	Reg.	183/38439	13/4452	23 (-35–56)
Nohynek	2.5-<3.5	2015/16	Reg.	272/38288	17/3470	34 (-8.1–60)
Kildegaard	2-<6	2021/22	IP&OP	473/3386	28/339	26 (-16–53)
Random-effects model						26 (5.6–42)
Fixed-effects model						26 (5.6–42)
						(I^2=0%, Q=0)

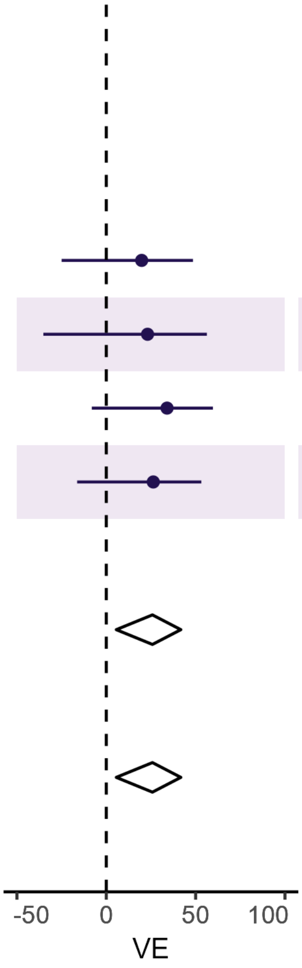

©2025 Goldsmith JJ et al. *JAMA Network Open*.

eFigure 11. Pooled IIV VE estimates against influenza for partially and fully vaccinated children <9 years and the difference between these estimates. The reference group is children <9 years who were not vaccinated in the current season and who's vaccine history is unknown.

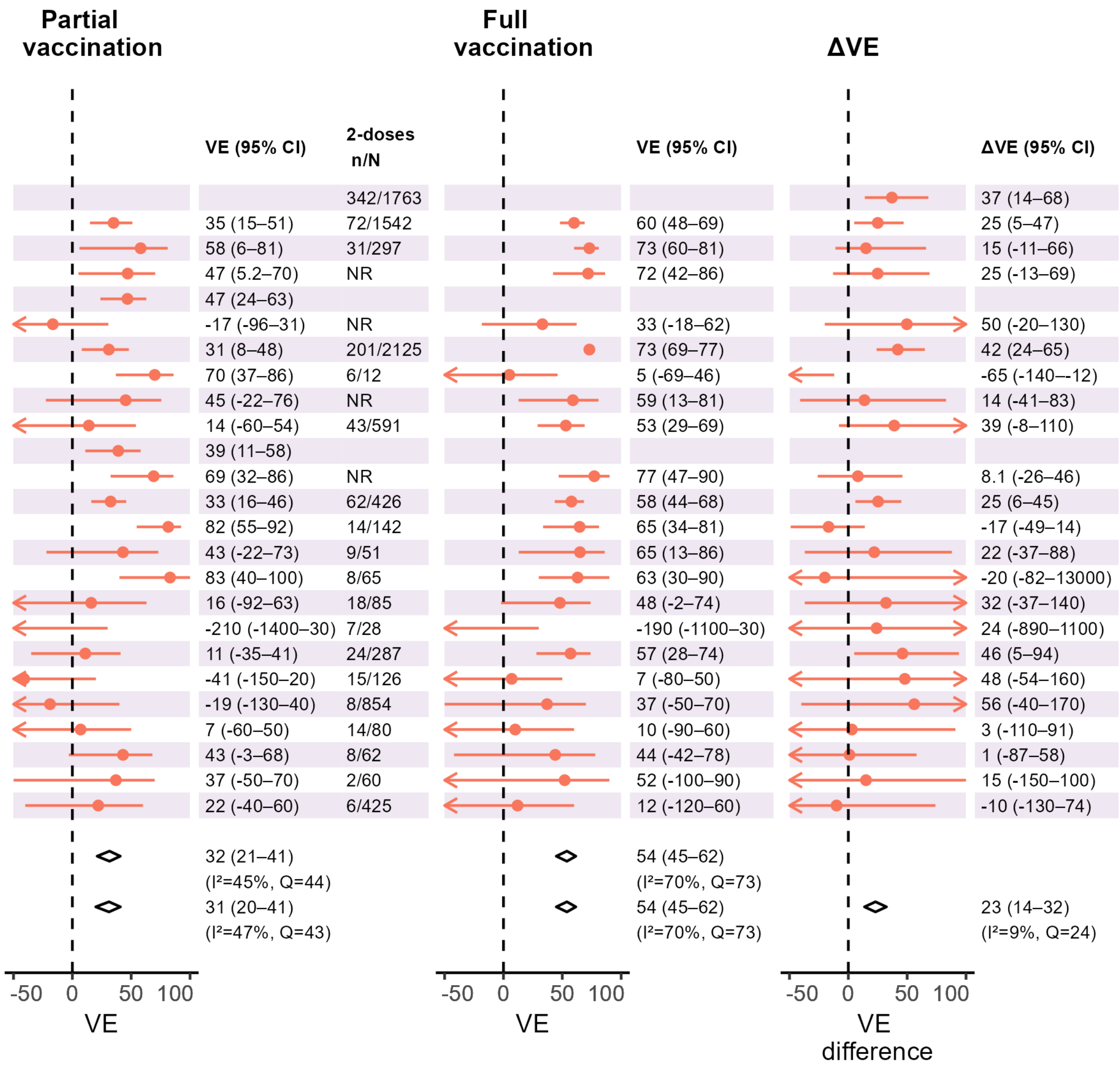

Study	Age	Years	Pop.	Unvaccinated n/N	1-dose n/N	VE (95% CI)	2-doses n/N	VE (95% CI)	ΔVE (95% CI)
Shrestha	0.5-<9	2022	OTR	5221/15685	61/198		342/1763		37 (14–68)
Thangarajah	0.5-<6	2018	Reg.	991/9360	62/868	35 (15–51)	72/1542	60 (48–69)	25 (5–47)
Yeung	0.5-<6	2015/16	IP	696/2553	7/50	58 (6–81)	31/297	73 (60–81)	15 (-11–66)
Buchan	0.5-<5	2013/14	IP	NR	NR	47 (5.2–70)	NR	72 (42–86)	25 (-13–69)
Yildirim	0.5-<9	2012/13-16/17	IP	358/639	81/203	47 (24–63)			
Buchan	0.5-<5	2012/13	IP	NR	NR	-17 (-96–31)	NR	33 (-18–62)	50 (-20–130)
Chua	0.5-<9	2011/12-18/19	IP	3579/20617	72/445	31 (8–48)	201/2125	73 (69–77)	42 (24–65)
Wang	0.5-<3	2011/12	IP	280/534	6/38	70 (37–86)	6/12	5 (-69–46)	-65 (-140–-12)
Buchan	0.5-<5	2011/12	IP	NR	NR	45 (-22–76)	NR	59 (13–81)	14 (-41–83)
Thompson	0.5-<9	2011/12	OP	132/715	15/135	14 (-60–54)	43/591	53 (29–69)	39 (-8–110)
Katz	0.5-<9	2010/11-12/13	IP&OP	NR	NR	39 (11–58)			
Buchan	0.5-<5	2010/11	IP	NR	NR	69 (32–86)	NR	77 (47–90)	8.1 (-26–46)
Yang	0.5-<5	2010	OTR	635/2268	26/168	33 (16–46)	62/426	58 (44–68)	25 (6–45)
Blyth	0.5-<5	2008,2010-2012	OP	294/1282	6/90	82 (55–92)	14/142	65 (34–81)	-17 (-49–14)
Staat	0.5-<5	2006/07	IP&OP	49/142	15/67	43 (-22–73)	9/51	65 (13–86)	22 (-37–88)
Cochran	0.5-<2	2005/06	Reg.	45/189	5/77	83 (40–100)	8/65	63 (30–90)	-20 (-82–13000)
Staat	0.5-<5	2005/06	IP&OP	56/144	11/39	16 (-92–63)	18/85	48 (-2–74)	32 (-37–140)
Cochran	0.5-<2	2004/05	Reg.	12/68	10/57	-210 (-1400–30)	7/28	-190 (-1100–30)	24 (-890–1100)
Eisenberg	0.5-<6	2004/05	IP&OP	130/900	43/315	11 (-35–41)	24/287	57 (28–74)	46 (5–94)
Szilagyi	0.5-<5	2004/05	OP	61/503	19/113	-41 (-150–20)	15/126	7 (-80–50)	48 (-54–160)
Szilagyi	0.5-<5	2004/05	IP	54/3154	18/700	-19 (-130–40)	8/854	37 (-50–70)	56 (-40–170)
Cochran	0.5-<2	2003/04	Reg.	167/893	32/191	7 (-60–50)	14/80	10 (-90–60)	3 (-110–91)
Eisenberg	0.5-<6	2003/04	IP&OP	198/754	22/156	43 (-3–68)	8/62	44 (-42–78)	1 (-87–58)
Szilagyi	0.5-<5	2003/04	OP	66/549	6/87	37 (-50–70)	2/60	52 (-100–90)	15 (-150–100)
Szilagyi	0.5-<5	2003/04	IP	144/3810	15/525	22 (-40–60)	6/425	12 (-120–60)	-10 (-130–74)
Random-effects model, all estimates						32 (21–41) (I²=45%, Q=44)		54 (45–62) (I²=70%, Q=73)	
Random-effects model, ΔVE estimates						31 (20–41) (I²=47%, Q=43)		54 (45–62) (I²=70%, Q=73)	23 (14–32) (I²=9%, Q=24)

Note: $\Delta VE = VE_2 - VE_1$. $\Delta VE > 0$ indicates greater effectiveness for for fully vaccinated. Partially vaccinated received one dose in the current season and were previously influenza-vaccine-naïve. Fully vaccinated received ≥2 doses including ≥1 in the current season. Pop. = population, IP = inpatient, OP = outpatient, Reg. = registry, OTR = other.

©2025 Goldsmith JJ et al. *JAMA Network Open.*

eFigure 12. Pooled IIV VE estimates against influenza for partially and fully vaccinated children <3 years and the difference between these estimates. The reference group is children <3 years who were not vaccinated in the current season and who's vaccine history is unknown.

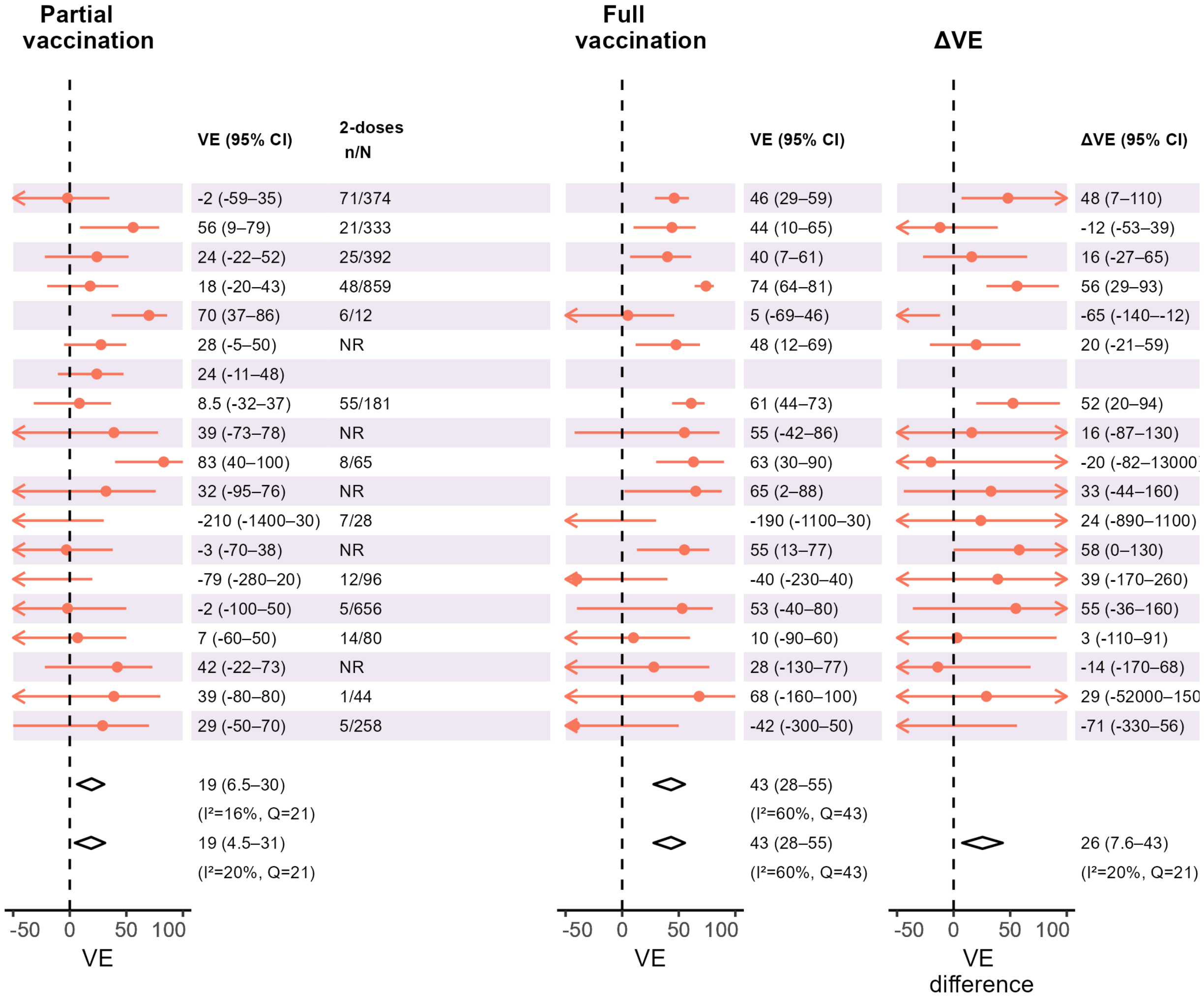

Study	Age	Years	Pop.	Unvaccinated n/N	1-dose n/N	VE (95% CI)	2-doses n/N	VE (95% CI)	ΔVE (95% CI)
Shrestha	0.5-<2.5	2022	OTR	1032/3403	36/103	-2 (-59–35)	71/374	46 (29–59)	48 (7–110)
Thangarajah	2-<3	2018	Reg.	184/1716	8/162	56 (9–79)	21/333	44 (10–65)	-12 (-53–39)
Thangarajah	0.5-<2	2018	Reg.	236/2279	22/270	24 (-22–52)	25/392	40 (7–61)	16 (-27–65)
Chua	0.5-<3	2011/12-2018/19	IP	1513/11657	39/292	18 (-20–43)	48/859	74 (64–81)	56 (29–93)
Wang	0.5-<3	2011/12	IP	280/534	6/38	70 (37–86)	6/12	5 (-69–46)	-65 (-140–-12)
Buchan	0.5-<2	2010/11 - 2013/14	IP	NR	NR	28 (-5–50)	NR	48 (12–69)	20 (-21–59)
Yang	0.5-<2	2010	OTR	274/1111	9/54	24 (-11–48)			
Yang	0.5-<2	2009	OTR	637/1694	15/32	8.5 (-32–37)	55/181	61 (44–73)	52 (20–94)
Staat	0.5-<2	2006/07	IP&OP	NR	NR	39 (-73–78)	NR	55 (-42–86)	16 (-87–130)
Cochran	0.5-<2	2005/06	Reg.	45/189	5/77	83 (40–100)	8/65	63 (30–90)	-20 (-82–13000
Staat	0.5-<2	2005/06	IP&OP	NR	NR	32 (-95–76)	NR	65 (2–88)	33 (-44–160)
Cochran	0.5-<2	2004/05	Reg.	12/68	10/57	-210 (-1400–30)	7/28	-190 (-1100–30)	24 (-890–1100)
Eisenberg	0.5-<2	2004/05	IP&OP	NR	NR	-3 (-70–38)	NR	55 (13–77)	58 (0–130)
Szilagyi	0.5-<2	2004/05	OP	14/162	16/93	-79 (-280–20)	12/96	-40 (-230–40)	39 (-170–260)
Szilagyi	0.5-<2	2004/05	IP	31/1107	15/562	-2 (-100–50)	5/656	53 (-40–80)	55 (-36–160)
Cochran	0.5-<2	2003/04	Reg.	167/893	32/191	7 (-60–50)	14/80	10 (-90–60)	3 (-110–91)
Eisenberg	0.5-<2	2003/04	IP&OP	NR	NR	42 (-22–73)	NR	28 (-130–77)	-14 (-170–68)
Szilagyi	0.5-<2	2003/04	OP	30/242	4/59	39 (-80–80)	1/44	68 (-160–100)	29 (-52000–150
Szilagyi	0.5-<2	2003/04	IP	76/1747	9/330	29 (-50–70)	5/258	-42 (-300–50)	-71 (-330–56)
Random-effects model, all estimates						19 (6.5–30) (I²=16%, Q=21)		43 (28–55) (I²=60%, Q=43)	
Random-effects model, ΔVE estimates						19 (4.5–31) (I²=20%, Q=21)		43 (28–55) (I²=60%, Q=43)	26 (7.6–43) (I²=20%, Q=21)

Note: $\Delta VE = VE_2 - VE_1$. $\Delta VE > 0$ indicates greater effectiveness for for fully vaccinated. Partially vaccinated received one dose in the current season and were previously influenza-vaccine-naïve. Fully vaccinated received ≥2 doses including ≥1 in the current season. Pop. = population, IP = inpatient, OP = outpatient, Reg. = registry, OTR = other.

eAppendix 3. Monovalent A(H1N1)pdm09 vaccine sub-analysis

Results

All five of the IIV monovalent A(H1N1)pdm09 VE studies,[2, 3, 4, 5, 6] were from the first year of A(H1N1)pdm09 circulation in North America (2009/10) (Table 1). Three of the studies [2, 5, 6] used an adjuvanted monovalent A(H1N1)pdm09 vaccine and two [3, 4] used a non-adjuvanted monovalent A(H1N1)pdm09 vaccine. VE point estimates reported by the studies using adjuvanted vaccine were higher than the VE point estimates from the studies using the unadjuvanted vaccine in 2009/10.

Two of the A(H1N1)pdm09 monovalent studies (eFigure 16) were judged to have a critical risk of bias due to potential uncontrolled confounding. Insufficient estimates were available to calculate pooled VE for the adjuvanted monovalent A(H1N1)pdm09 or the non-adjuvanted A(H1N1)pdm09 vaccine (eFigure 13).

Discussion

The estimates for one-dose IIV 2009 adjuvanted monovalent A(H1N1)pdm09 effectiveness against A(H1N1)pdm09 in 2009/10 were high and statistically significant. This finding supports the decision made by some jurisdictions to use a single dose of adjuvanted vaccine for children during the initial waves of a pandemic, when vaccine supply and time were limited, to maximise the number of children protected.[2]

eFigure 13. Adjuvanted and non-adjuvanted monovalent IIV VE estimates against A(H1N1)pdm09 influenza for children <9 years who have received one dose of vaccine for the first time in 2009/10. The reference group is children <9 years who have never been vaccinated for A(H1N1)pdm09 influenza but may have been vaccinated previously against seasonal H1N1.

Adjuvanted monovalent pandemic vaccine

Study	Age	Pop.	Unvaccinated n/N	1-dose n/N	VE (95% CI)
Gilca	0.5-<9	OTR	63/663	9/221	85 (61–94)
Mahmud	0.5-<3	Reg.	NR	NR	97 (72–100)

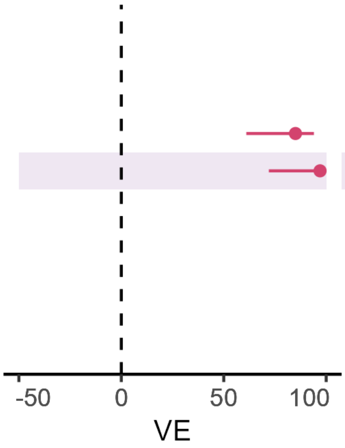

Non-adjuvanted monovalent pandemic vaccine

Study	Age	Pop.	Unvaccinated n/N	1-dose n/N	VE (95% CI)
Hadler	3-<9	OTR	44/413	1/26	66 (-120–99)

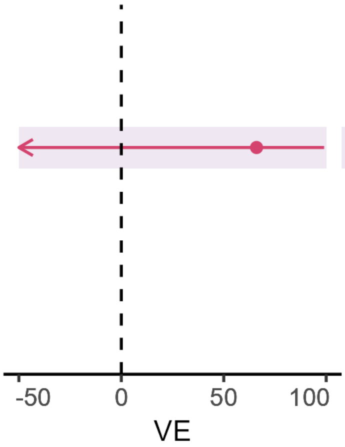

Note: Pop. = population, Reg. = registry, OTR = other.

©2025 Goldsmith JJ et al. *JAMA Network Open*.

eTable 2. Comparison of fixed effect model pooled estimates with random effect model pooled estimates for vaccine efficacy studies.

					Random effects model		Fixed effects model	
Vaccine	Age (years)	Influenza type	Intervention	N	Central estimate	95% CI	Central estimate	95% CI
IIV	<9	Any	2 doses	15	52%	43% – 60%	47%	42% – 51%
	<3	Any	2 doses	12	51%	41% – 60%	45%	40% – 51%
		A	2 doses	2	51%	44% – 58%	51%	44% – 58%
		A(H1N1)pdm09	2 doses	7	63%	44% – 75%	63%	51% – 73%
		H3N2	2 doses	7	45%	36% – 53%	45%	36% – 53%
		B	2 doses	3	50%	37% – 60%	49%	39% – 57%
		B(Vic.)	2 doses	5	28%	-11% – 53%	28%	-11% – 53%
		B(Yam.)	2 doses	5	51%	40% – 61%	51%	40% – 61%
LAIV	<9	Any	1 dose	7	51%	39% – 60%	48%	41% – 54%
			2 doses	4	82%	69% – 89%	76%	71% – 80%
		H3N2	1 dose	3	60%	48% – 70%	60%	50% – 68%
			2 doses	3	86%	66% – 94%	79%	71% – 84%
		B	2 doses	4	75%	46% – 89%	67%	55% – 76%

©2025 Goldsmith JJ et al. *JAMA Network Open.*

eTable 3. Comparison of fixed effect model pooled estimates with random effect model pooled estimates for vaccine effectiveness studies.

						Random effects model		Fixed effects model	
Group	Vaccine	Influenza Type	Age (years)	Intervention	N	Central estimate	95% CI	Central estimate	95% CI
Naive	IIV	Any	<9	1 dose	7	35%	18% – 48%	36%	23% – 46%
				1 dose (Δ estimates only)	5	31%	14% – 44%	31%	16% – 44%
				2 doses	11	43%	34% – 50%	43%	35% – 49%
				2 doses (Δ estimates only)	5	43%	35% – 50%	43%	35% – 50%
				Δ 1 and 2 doses	5	15%	-3% – 33%	15%	-3% – 33%
			<3	1 dose	5	14%	-10% – 33%	15%	-4% – 32%
				1 dose (Δ estimates only)	4	8%	-25% – 32%	12%	-11% – 30%
				2 doses	10	41%	29% – 51%	42%	34% – 49%
				2 doses (Δ estimates only)	4	43%	34% – 50%	43%	34% – 50%
				Δ 1 and 2 doses	4	28%	5% – 51%	28%	5% – 51%
		A	<3	2 doses	12	61%	36% – 76%	51%	34% – 63%
		B	<3	1 dose	4	36%	1% – 58%	36%	2% – 58%
				2 doses	9	44%	23% – 60%	44%	23% – 60%
	LAIV	Any	<4	1 dose	4	26%	6% – 42%	26%	6% – 42%
Mixed history	IIV	Any	<9	Partial vaccination	25	32%	21% – 41%	31%	24% – 37%
				Partial vaccination (Δ estimates only)	24	31%	20% – 41%	31%	24% – 37%
				Full vaccination	23	54%	45% – 62%	61%	57% – 64%
				Full vaccination (Δ estimates only)	23	54%	45% – 62%	61%	57% – 64%
				Δ Full and partial vaccination	23	23%	14% – 32%	24%	16% – 32%
			<3	Partial vaccination	19	19%	7% – 30%	19%	8% – 29%
				Partial vaccination (Δ estimates only)	18	19%	5% – 31%	18%	6% – 29%
				Full vaccination	18	43%	28% – 55%	50%	43% – 56%
				Full vaccination (Δ estimates only)	18	43%	28% – 55%	50%	43% – 56%
				Δ Full and partial vaccination	18	26%	8% – 43%	28%	14% – 43%

Vaccine efficacy studies (RCTs) were assessed using the revised Cochrane risk of bias tool (RoB2).

eFigure 14. Risk of bias assessment for included RCTs

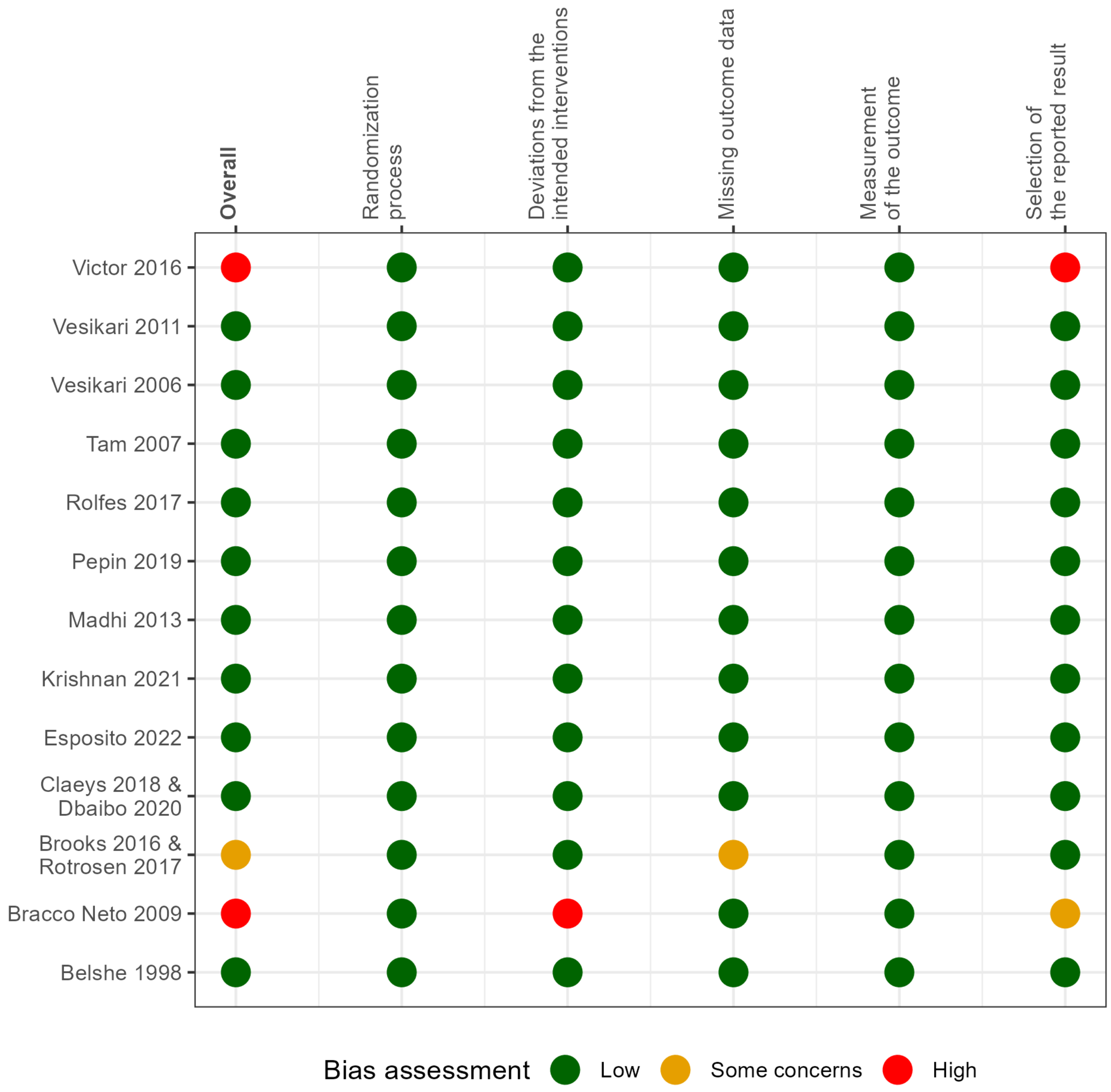

Vaccine effectiveness studies were assess using the Risk Of Bias In Non-randomized Studies-of Interventions tool (ROBINS-I).

eFigure 15. Risk of bias assessment for included vaccine effectiveness studies classified as naïve studies

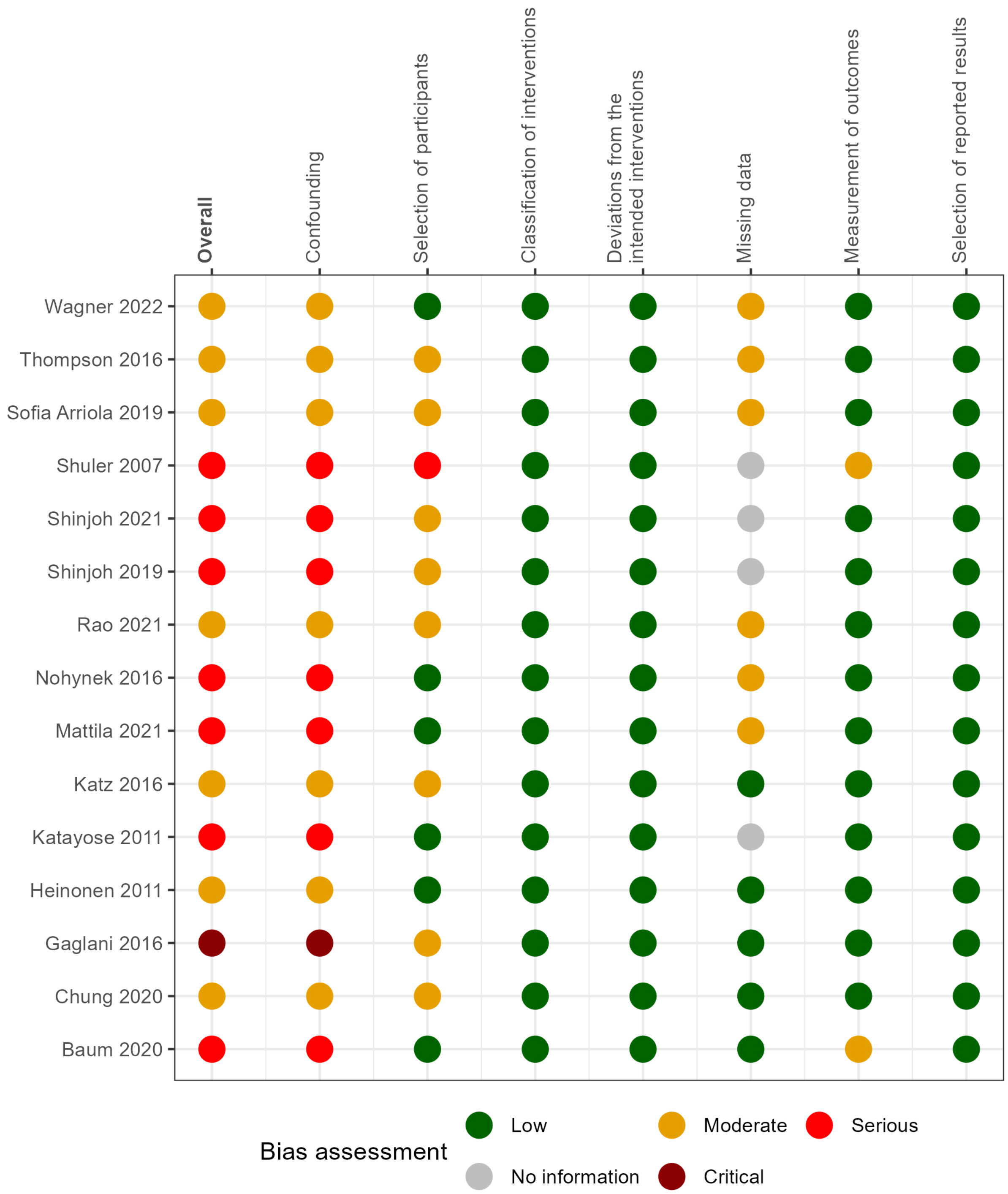

eFigure 16. Risk of bias assessment for included vaccine effectiveness studies classified as monovalent A(H1N1)pdm09 studies

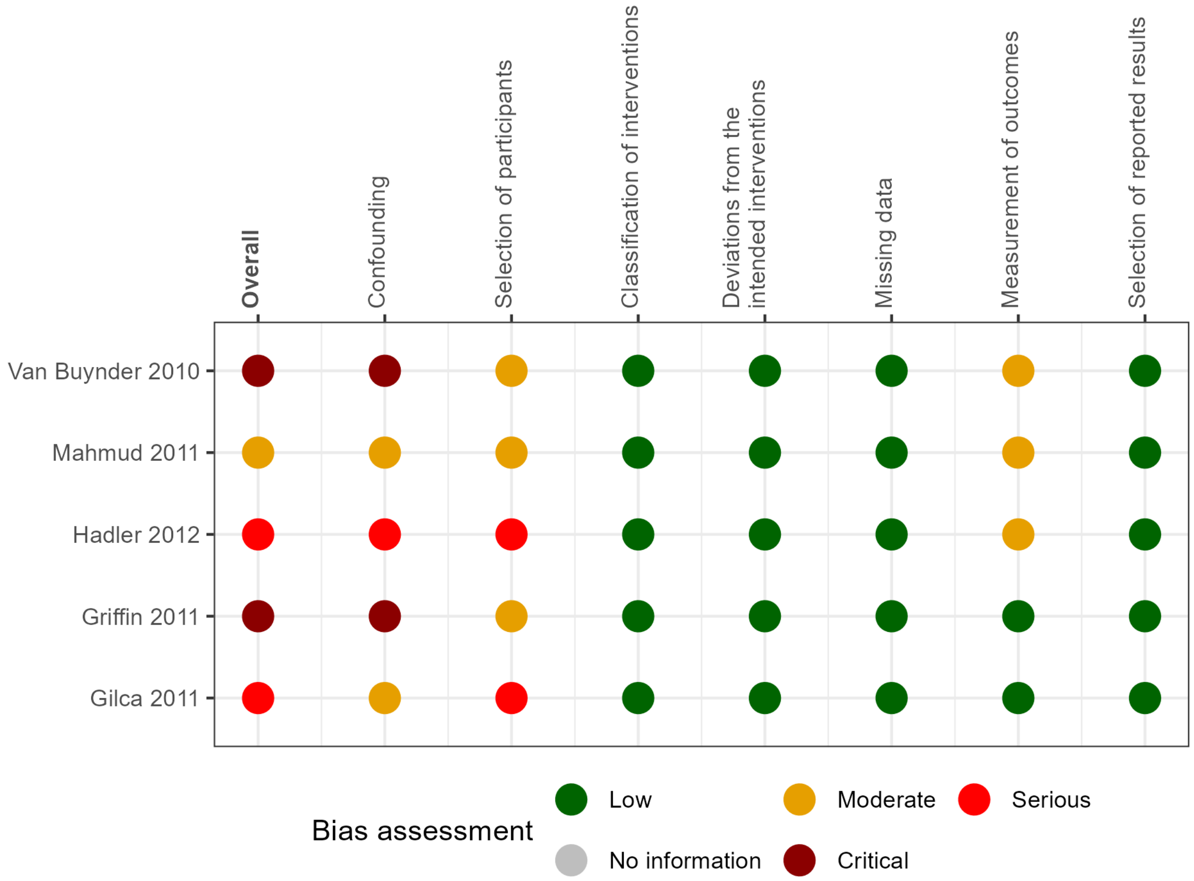

eFigure 17. Risk of bias assessment for included vaccine effectiveness studies classified as mixed history studies

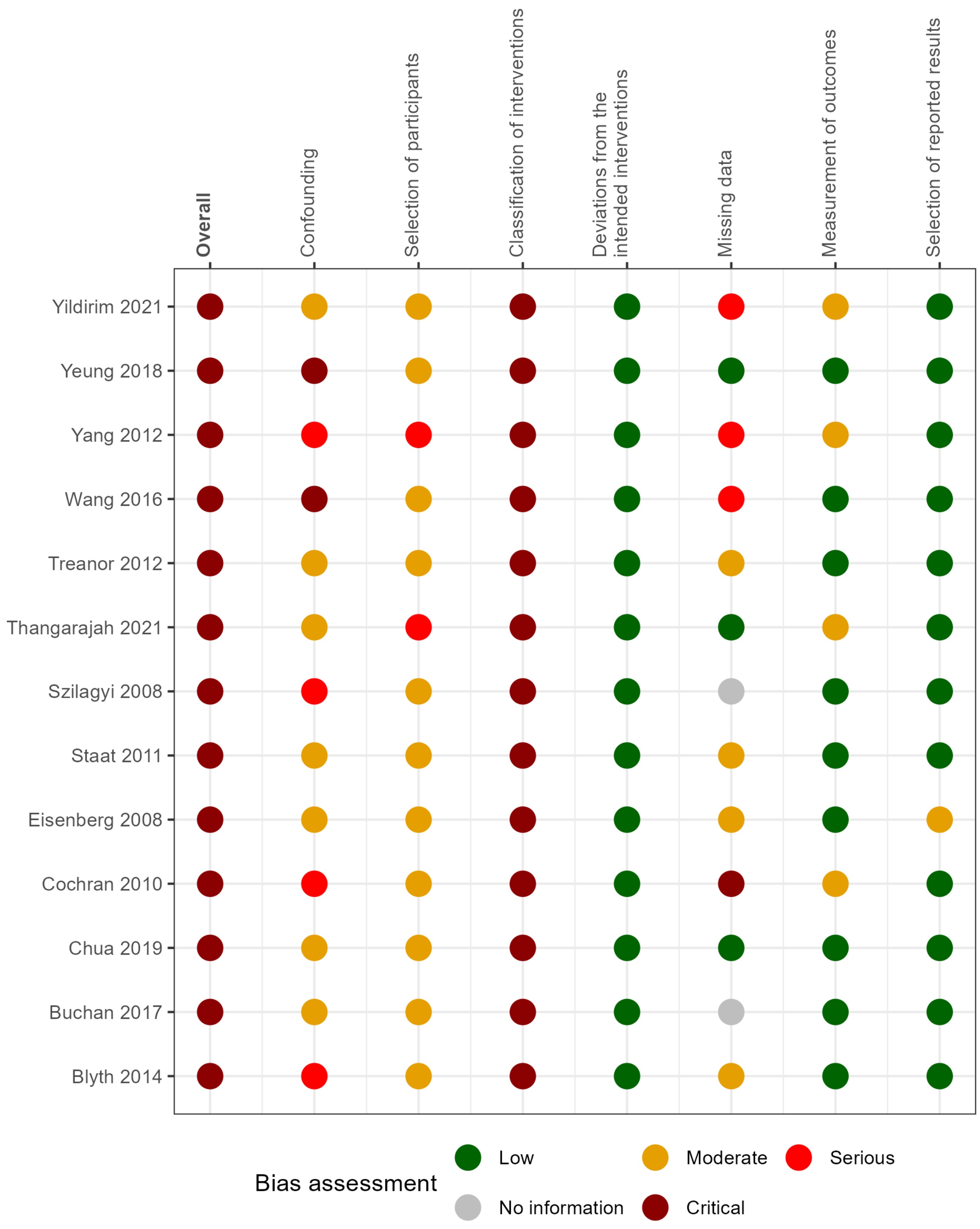

eAppendix 4. Classification of information bias in mixed-history studies

The mixed-history studies define unvaccinated as no influenza vaccine in the current season, partially vaccinated as one influenza vaccine dose in the current season and no prior history of influenza vaccination, and fully vaccinated as at least two vaccine doses, at least one of which occurred in the current season. These definitions of the interventions introduce two instances of differential and dependent misclassification at the conceptual level. First, unvaccinated in the current season is taken to correspond to the underlying conceptual construct of influenza vaccine naïvity. Second, fully vaccinated is taken to correspond to the underlying conceptual construct of two vaccine doses in the current season. This source of misclassification only arises in studies seeking to assess the relative protection of influenza vaccination by number of doses and depicted as a causal graph in eFigure 18. The direction of bias for partial vaccine effectiveness is anticipated to be towards the null, while the direction of bias for full vaccine effectiveness is anticipated to be away from the null. Naïve studies avoid this source of bias by adopting definitions with greater construct validity. Specifically, all children are required to have no prior history of influenza vaccination and influenza vaccination exposure for the current season is classified by dose i.e. zero, one or two.

eFigure 18. Graphical depiction of differential and dependent misclassification occurring in the mixed-history studies. *denotes misclassified.

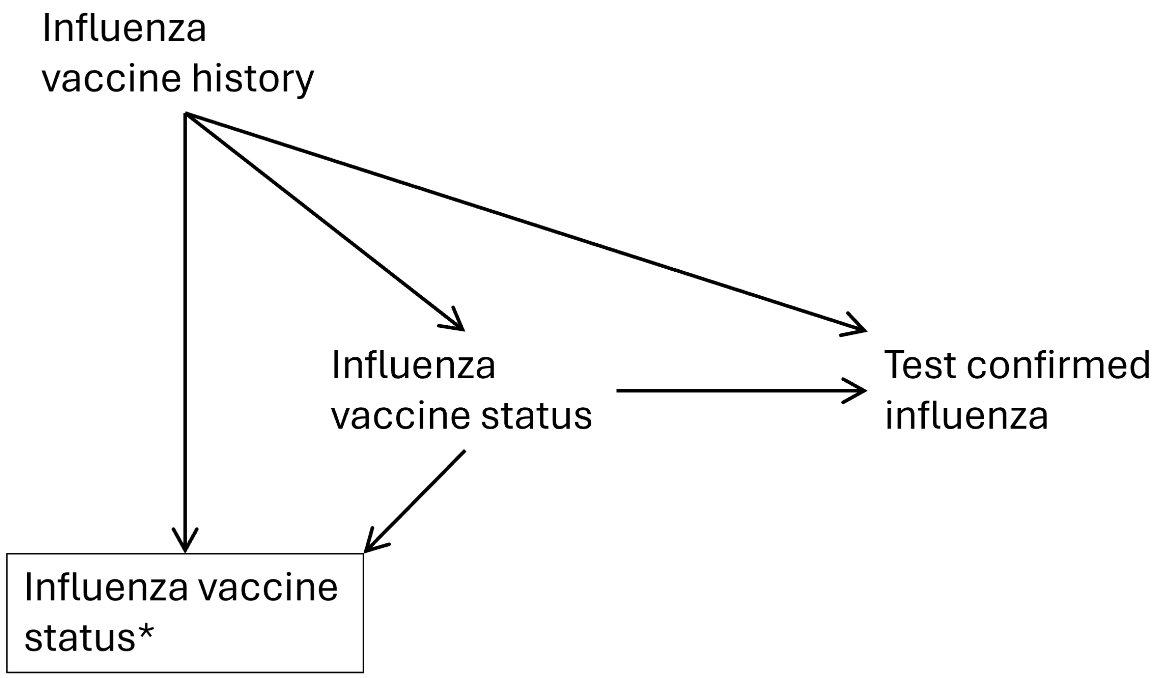

eAppendix 5. Publication bias

Funnel plots plot effect estimates (x axis) against the standard error of the effect estimate (y axis) which has been inverted, so that the studies with the least precision (the largest standard error) are closest to the x axis. If there is no publication bias, a funnel plot will show points distributed symmetrically in the shape of a funnel around the pooled effect estimate. This is because smaller studies will have greater variability due to chance, while as the studies get larger and therefore more precise, the estimates should converge. If this symmetry is missing, the meta-analysis result may be subject to publication bias.

For further information, refer to *The Cochrane Handbook for Systematic Reviews of Interventions: Chapter 13*[7].

eFigure 19. Funnel plots for each pooled analysis for any influenza by age group and by subtype for children <3 years old for two-dose IIV RCTs using a previously vaccine naive population.

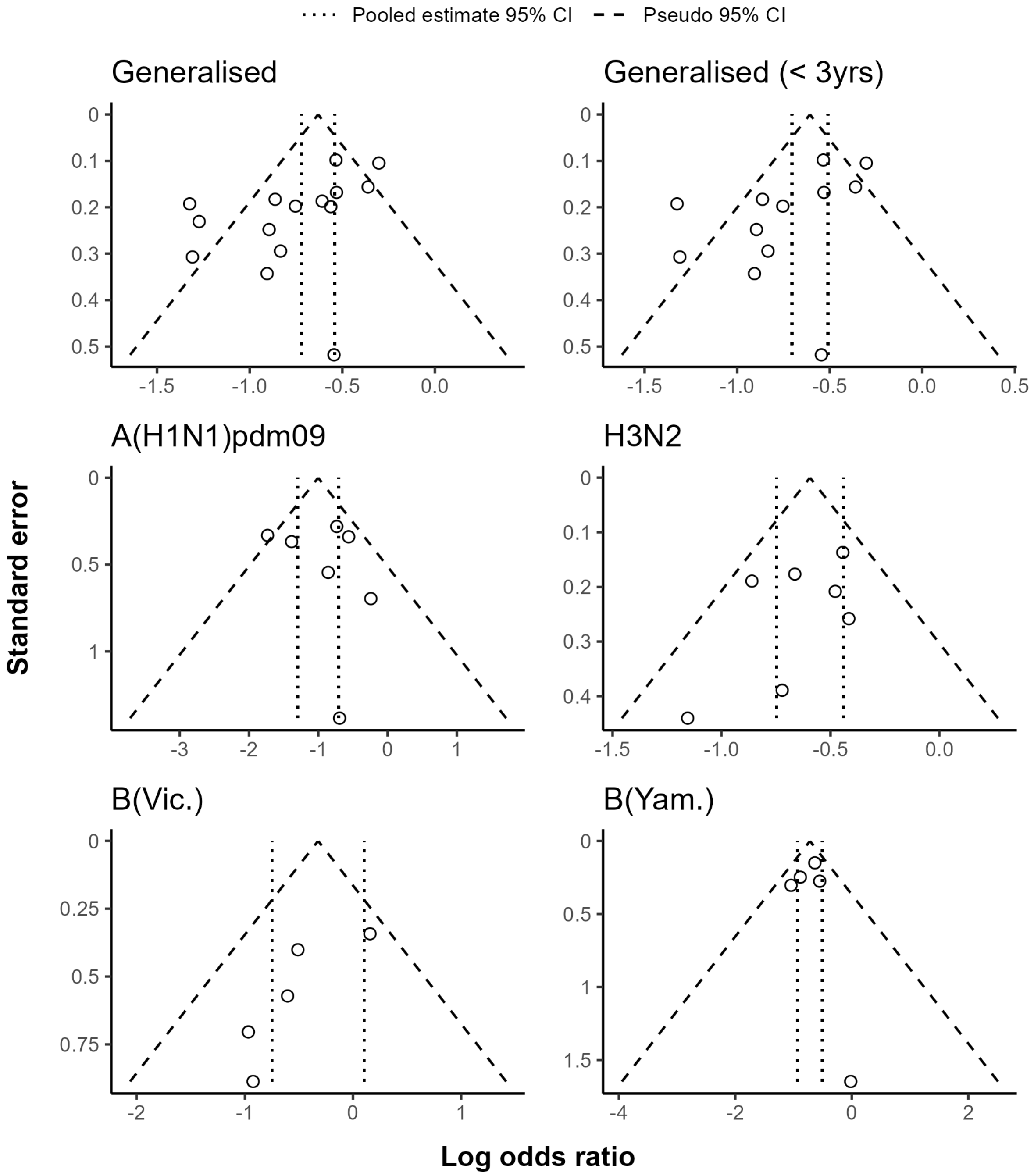

Note: All subtype efficacy estimates include fewer than 10 observations, which is lower than the number recommended by Cochrane for statistical testing.

eFigure 20. Funnel plot for pooled analysis for any influenza for children <3 years old for one-dose LAIV RCTs using a previously vaccine naive population.

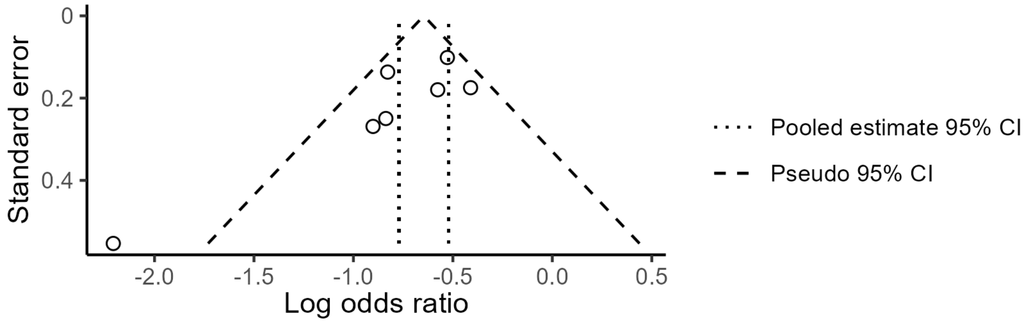

Note: Efficacy estimates include fewer than 10 observations, which is lower than the number recommended by Cochrane for statistical testing.

eFigure 21. Funnel plots for each pooled analysis by influenza type, age group and number of doses for IIV VE for studies using a previously vaccine naive population.

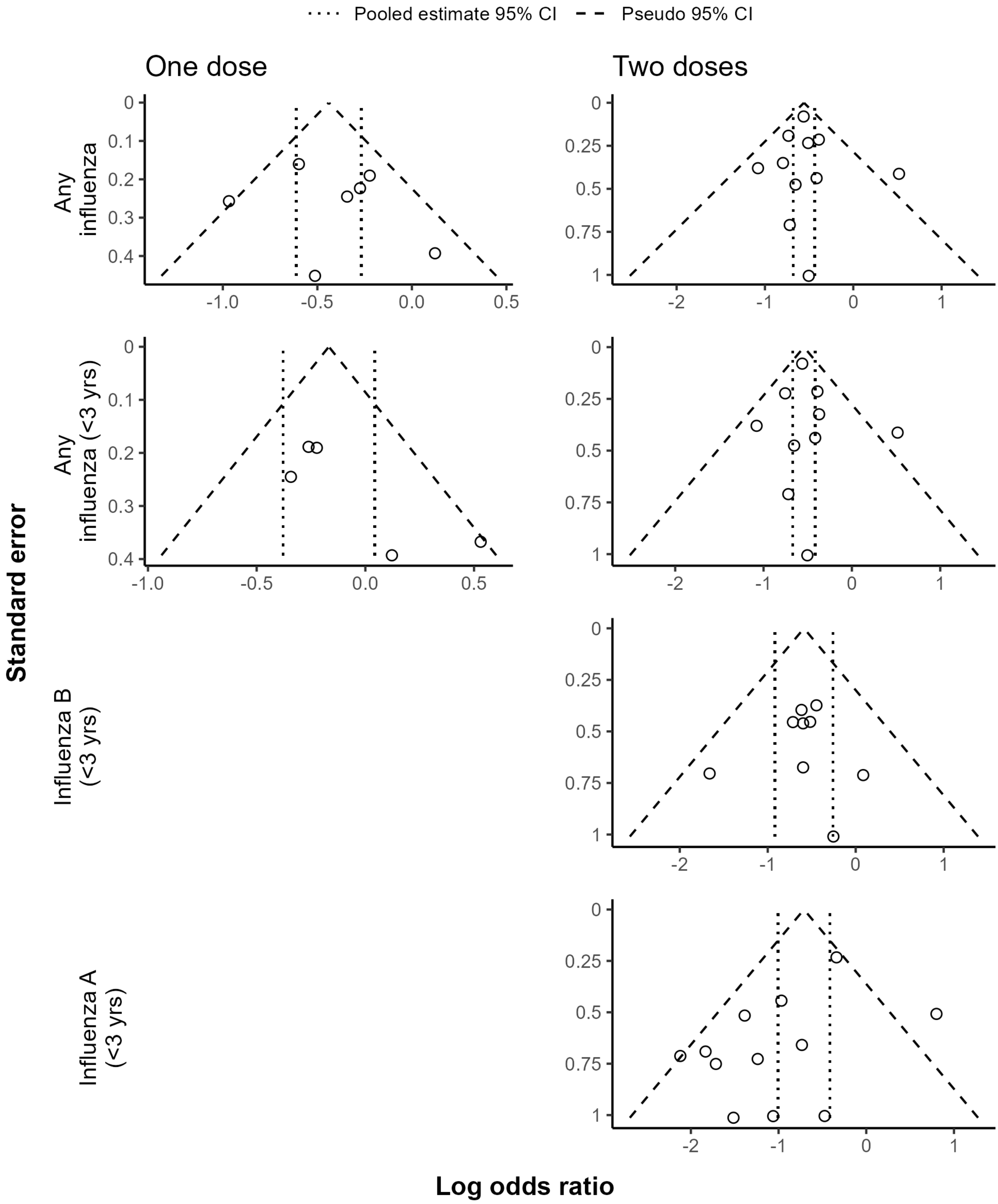

Note: All one-dose and influenza B effectiveness estimates include fewer than 10 observations, which is lower than the number recommended by Cochrane for statistical testing.

eFigure 22. Funnel plots for each pooled analysis by influenza type and age group for IIV VE for studies vulnerable to confounding as a result of not controlling for vaccine history in study arms other than the children vaccinated for the first time who only received one dose.

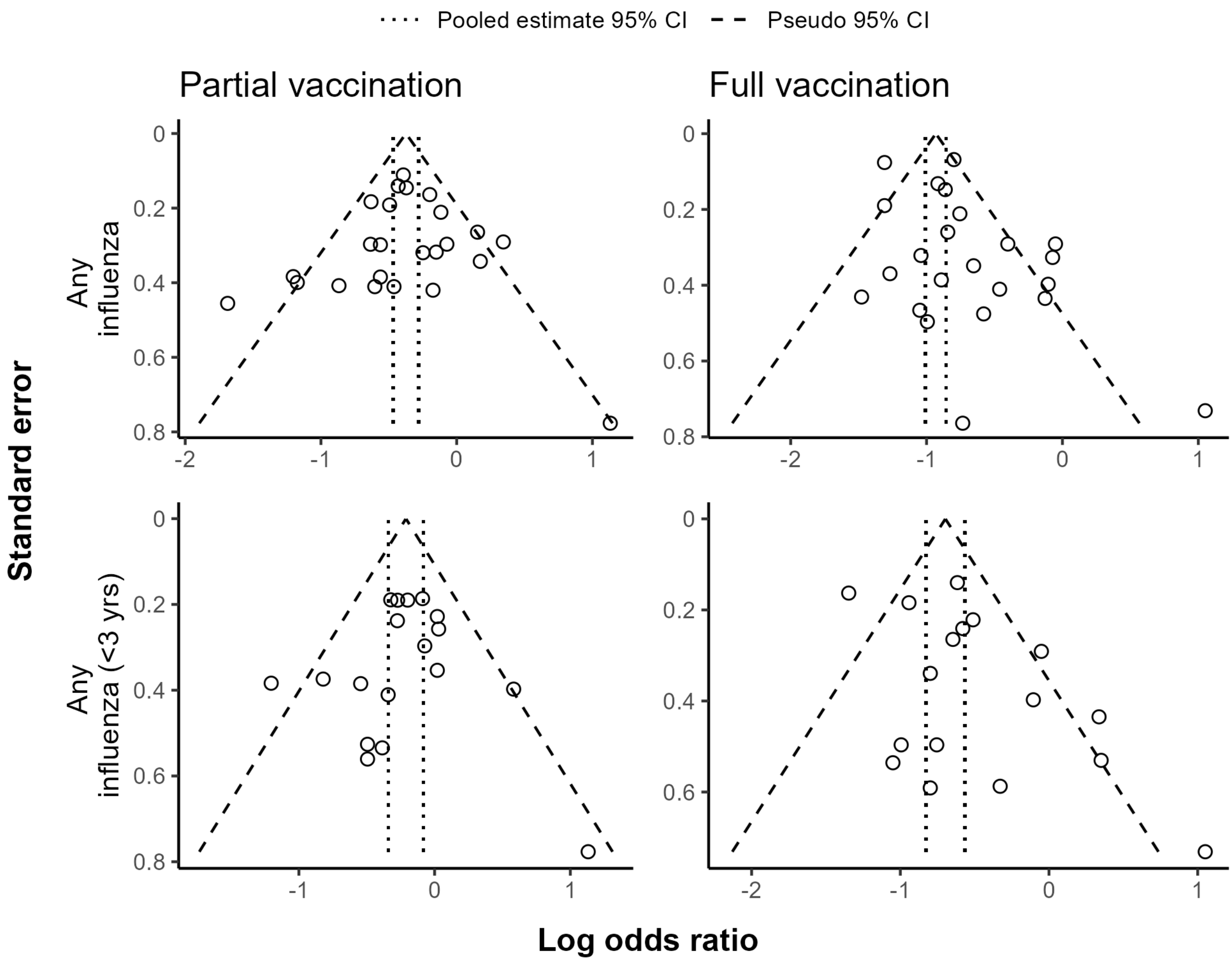

eTable 4. Egger's test for publication bias

Measure	Influenza type and age group	Vaccine type	Vaccination group	Egger's test value (95% CI)	p
Efficacy	Any (<9 yrs)	IIV	Two doses	-0.19% (-0.42%; 0.04%)	<0.001
	Any (<3 yrs)	IIV	Two doses	-0.21% (-0.45%; 0.02%)	<0.001
Effectiveness	Any (<9 yrs)	IIV	Two doses	-0.58% (-0.77%; -0.38%)	0.80
	Any (<3 yrs)	IIV	Two doses	-0.58% (-0.78%; -0.38%)	0.61
	Influenza A (<3 yrs)	IIV	Two doses	0.03% (-0.62%; 0.68%)	0.01
	Any (<9 yrs)	IIV	Partially vaccinated	-0.34% (-0.55%; -0.12%)	0.72
			Fully vaccinated*	-1.11% (-1.23%; -0.99%)	<0.001
	Any (<3 yrs)	IIV	Partially vaccinated	-0.17% (-0.51%; 0.18%)	0.78
			Fully vaccinated*	-1.1% (-1.39%; -0.82%)	0.002

Note: Statistical test for publication bias was only applied to categories with at least 10 estimates. The Egger's test null hypothesis is that symmetry exists in the funnel plot. A low p-value suggests the presence of asymmetry in the funnel plot and that the meta-analysis may be subject to publication bias.
*Only includes fully vaccinated estimates from papers that also reported partially vaccinated estimates.

©2025 Goldsmith JJ et al. *JAMA Network Open.*

eTable 5. Studies excluded at full text screening stage by reason

**Reason**	**Excluded studies**
reason	studies
Duplicates	Belshe et al. (2000)[8]; Belshe et al. (2001)[9]; Belshe et al. (2004)[10]; Block et al. (2009)[11]; Chung et al. (2019)[12]; Chung et al. (2025)[13]; Nakayama et al. (2024)[14]; Rigamonti et al. (2025)[15]; Vasil'eva et al. (1986)[16]; Whitaker et al. (2024)[17]; Zhang et al. (2024)[18]; Zhu et al. (2024)[19]
Not test con-firmed influenza	Allison et al. (2006)[20]; Boikos et al. (2021)[21]; Boikos et al. (2021)[21]; Carrat et al. (1998)[22]; Chen et al. (2018)[23]; Chumakov et al. (1986)[24]; Divino et al. (2020)[25]; Glenn et al. (2024)[26]; He et al. (2013)[27]; Hoberman et al. (2003)[28]; Hurwitz et al. (2000)[29]; Imran et al. (2022)[30]; Jick et al. (2010)[31]; Krishnarajah et al. (2021)[32]; Kulkarni et al. (2014)[33]; Matsuda et al. (2022)[34]; Okuno et al. (1966)[35]; Petrilli et al. (1971)[36]; Piedra et al. (2007)[37]; Rigamonti et al. (2025)[15]; Ritzwoller et al. (2005)[38]; Rudenko et al. (1996)[39]; Rudenko et al. (1996)[40]; Shibata et al. (2018)[41]; Shibata et al. (2019)[42]; Simpson et al. (2012)[43]; Uchida et al. (2017)[44]; Yin et al. (2011)[45]; Yokomichi et al. (2021)[46]; Yokomichi et al. (2023)[47]
Under 9s not reported	Alfelali et al. (2019)[48]; Awadalla et al. (2023)[49]; Balasubramani et al. (2020)[50]; Balasubramani et al. (2021)[51]; Baselga-Moreno et al. (2019)[52]; Bateman et al. (2013)[53]; Bellino et al. (2019)[54]; Belongia et al. (2009)[55]; Blanquart et al. (2025)[56]; Blyth et al. (2015)[57]; Boddington et al. (2019)[58]; Bruxvoort et al. (2019)[59]; Cantarutti et al. (2022)[60]; Carbó Ma et al. (2010)[61]; Carville et al. (2015)[62]; Castilla et al. (2006)[63]; Castilla et al. (2011)[64]; Castilla et al. (2012)[65]; Castilla et al. (2013)[66]; Castilla et al. (2013)[67]; Castilla et al. (2016)[68]; Castilla et al. (2016)[69]; Castilla et al. (2020)[70]; Cervi et al. (1997)[71]; Chan et al. (2019)[72]; Chan et al. (2021)[73]; Cheng et al. (2011)[74]; Cheng et al. (2013)[75]; Cheng et al. (2014)[76]; Cheng et al. (2019)[77]; Cheng et al. (2019)[77]; Cheng et al. (2022)[78]; Chung et al. (2016)[79]; Chung et al. (2024)[80]; Chung et al. (2024)[81]; Colucci et al. (2019)[82]; Costantino et al. (2024)[83]; Cowling et al. (2010)[84]; Cowling et al. (2012)[85]; Cowling et al. (2014)[86]; Darvishian et al. (2017)[87]; DeMarcus et al. (2016)[88]; DeMarcus et al. (2019)[89]; Dogliani et al. (1997)[90]; Dominguez et al. (2012)[91]; Domnich et al. (2024)[92]; Edwards et al. (1994)[93]; El'shina et al. (2000)[94]; Emborg et al. (2012)[95]; Englund et al. (2013)[96]; Falchi et al. (2013)[97]; Ferdinands et al. (2014)[98]; Fielding et al. (2011)[99]; Fielding et al. (2011)[100]; Fielding et al. (2012)[101]; Fielding et al. (2016)[102]; Flannery et al. (2018)[103]; Fleming et al. (2010)[104]; Frutos et al. (2024)[105]; Frutos et al. (2025)[106]; Gaglani et al. (2021)[107]; Galtier et al. (2015)[108]; Garcia-Garcia et al. (2009)[109]; Gattas et al. (2015)[110]; Gharpure et al. (2025)[111]; Gherasim et al. (2016)[112]; Guzmán Her et al. (2012)[113]; Hallmann-Szel et al. (2018)[114]; Hardelid et al. (2011)[115]; Hekimoglu et al. (2018)[116]; Hu et al. (2021)[117]; Jackson et al. (2017)[118]; Jackson et al. (2021)[119]; Jiménez- et al. (2012)[120]; Jiménez- et al. (2013)[121]; Jiménez- et al. (2015)[122]; Joshi et al. (2012)[123]; Kamada et al. (2006)[124]; Kavanagh et al. (2011)[125]; Kavanagh et al. (2013)[126]; Kawai et al. (2003)[127]; Kelly et al. (2009)[128]; Kelly et al. (2016)[129]; Kersellius et al. (2020)[130]; Kim et al. (2022)[131]; King et al. (2024)[132]; Kissling et al. (2011)[133]; Kissling et al. (2013)[134]; Kissling et al. (2014)[135]; Kissling et al. (2018)[136]; Kissling et al. (2019)[137]; Kissling et al. (2019)[138]; Kissling et al. (2023)[139]; Klein et al. (2020)[140]; Klick et al. (2013)[141]; Kotecha et al. (2016)[142]; Kurecic Fili et al. (2015)[143]; Lei et al. (2024)[144]; Lei et al. (2025)[145]; Lo et al. (2013)[146]

Continued on next page

©2025 Goldsmith JJ et al. *JAMA Network Open*.

eTable 5 Continued from previous page

Reason	Excluded studies
Under 9s not reported (continued)	Loeb et al. (2010)[147]; Lytras et al. (2015)[148]; Lytras et al. (2016)[149]; Ma et al. (2017)[150]; Ma et al. (2025)[151]; Machado et al. (2021)[152]; Martíne et al. (2013)[153]; Martíne et al. (2015)[154]; Martíne et al. (2017)[155]; Martíne et al. (2019)[156]; Martíne et al. (2021)[157]; Martíne et al. (2022)[158]; Martíne et al. (2023)[159]; Martíne et al. (2025)[160]; Maurel et al. (2024)[161]; Maurel et al. (2024)[162]; McAnerney et al. (2015)[163]; McAnerney et al. (2015)[164]; McAnerney et al. (2017)[165]; McLean et al. (2017)[166]; McLean et al. (2018)[167]; Menniti-Ippolito et al. (2014)[168]; Mir et al. (2021)[169]; Mouratidou et al. (2020)[170]; Mulpuru et al. (2019)[171]; Nakayama et al. (2024)[14]; Nation et al. (2021)[172]; Neuzil et al. (2001)[173]; Nicholls et al. (2004)[174]; Noble et al. (2023)[175]; Nolan et al. (2021)[176]; Norman et al. (2022)[177]; Ntshoe et al. (2014)[178]; Nunes et al. (2014)[179]; Ogokeh et al. (2021)[180]; Ogra et al. (1977)[181]; Paradowska-Stankiewicz et al. (2018)[182]; Pebody et al. (2011)[183]; Pebody et al. (2016)[184]; Pebody et al. (2017)[185]; Pebody et al. (2019)[186]; Pebody et al. (2020)[187]; Philip et al. (1969)[188]; Pierse et al. (2016)[189]; Poehling et al. (2018)[190]; Puig-Ba et al. (2010)[191]; Puig-Ba et al. (2016)[192]; Puig-Ba et al. (2016)[192]; Radin et al. (2016)[193]; Ray et al. (2019)[194]; Redlberger-Fritz et al. (2016)[195]; Redlberger-Fritz et al. (2020)[196]; Regan et al. (2019)[197]; Regan et al. (2019)[198]; Rizzo et al. (2016)[199]; Rizzo et al. (2020)[200]; Rose et al. (2025)[201]; Saito et al. (2017)[202]; Salleras et al. (2006)[203]; Sanada et al. (2016)[204]; Savulescu et al. (2011)[205]; Savulescu et al. (2011)[206]; Savulescu et al. (2014)[207]; Schuurmans et al. (2011)[208]; Separovic et al. (2025)[209]; Shang et al. (2018)[210]; Shinjoh et al. (2015)[211]; Shinjoh et al. (2018)[212]; Simpson et al. (2010)[213]; Simpson et al. (2015)[214]; Skowronski et al. (2005)[215]; Skowronski et al. (2007)[216]; Skowronski et al. (2012)[217]; Skowronski et al. (2014)[218]; Skowronski et al. (2014)[219]; Skowronski et al. (2015)[220]; Skowronski et al. (2016)[221]; Skowronski et al. (2017)[222]; Skowronski et al. (2019)[223]; Skowronski et al. (2019)[224]; Skowronski et al. (2023)[225]; Slepushkin et al. (1993)[226]; Slobodniuk et al. (2002)[227]; Smith et al. (2020)[228]; Smithgall et al. (2016)[229]; Sohn et al. (2020)[230]; Soldevila et al. (2022)[231]; Steens et al. (2011)[232]; Stuurman et al. (2020)[233]; Su et al. (2023)[234]; Sullivan et al. (2014)[235]; Sullivan et al. (2014)[236]; Sullivan et al. (2016)[237]; Suzuki et al. (2014)[238]; Sykes et al. (2017)[239]; Tam et al. (2018)[240]; Thomas et al. (2014)[241]; Thompson et al. (2013)[242]; Thompson et al. (2013)[242]; Thors et al. (2025)[243]; Torner et al. (2015)[244]; Turner et al. (2014)[245]; Uphoff et al. (2011)[246]; Uzicanin et al. (2012)[247]; Vaikutyte et al. (2023)[248]; Valenciano et al. (2011)[249]; Valenciano et al. (2015)[250]; Valenciano et al. (2016)[251]; Valenciano et al. (2018)[252]; Vasileiou et al. (2020)[253]; Vasilyeva et al. (1986)[254]; Vilcu et al. (2018)[255]; Wang et al. (2016)[256]; Wang et al. (2020)[257]; Whitaker et al. (2024)[258]; Whitaker et al. (2024)[259]; Whitaker et al. (2024)[17]; Wichmann et al. (2010)[260]; Yang et al. (2014)[261]; Yaron-Yakoby et al. (2018)[262]; Yoon et al. (2021)[263]; Zaplatnikov et al. (2001)[264]; Zhang et al. (2017)[265]; Zhang et al. (2018)[266]; Zhang et al. (2020)[267]; Zhang et al. (2024)[268]; Zhu et al. (2024)[269]; de Lus et al. (2021)[270]; van et al. (2017)[271]; Ört et al. (2011)[272]; Ört et al. (2012)[273]

Continued on next page

©2025 Goldsmith JJ et al. *JAMA Network Open*.

eTable 5 Continued from previous page

**Reason**	**Excluded studies**
Vaccine history not accounted for	Adams et al. (2024)[274]; Anderson et al. (2018)[275]; Ando et al. (2018)[276]; Ando et al. (2019)[277]; Ando et al. (2020)[278]; Ando et al. (2021)[279]; Andrews et al. (2014)[280]; Belongia et al. (2011)[281]; Bi et al. (2024)[282]; Blyth et al. (2016)[283]; Blyth et al. (2016)[284]; Blyth et al. (2019)[285]; Blyth et al. (2020)[286]; Boddington et al. (2022)[287]; Buchan et al. (2018)[288]; Campbell et al. (2020)[289]; Campbell et al. (2020)[290]; Campbell et al. (2021)[291]; Caspard et al. (2016)[292]; Castillejos et al. (2019)[293]; Chard et al. (2023)[294]; Chiu et al. (2016)[295]; Chiu et al. (2018)[296]; Chiu et al. (2020)[297]; Chon et al. (2019)[298]; Chua et al. (2021)[299]; Chung et al. (2025)[13]; Clover et al. (1991)[300]; Colucci et al. (2020)[301]; Cowling et al. (2016)[302]; Cowling et al. (2023)[303]; Diallo et al. (2019)[304]; Dixon et al. (2010)[305]; Drori et al. (2020)[306]; El O et al. (2015)[307]; El O et al. (2018)[308]; Feldstein et al. (2021)[309]; Feng et al. (2018)[310]; Flannery et al. (2016)[311]; Flannery et al. (2017)[312]; Flannery et al. (2019)[313]; Flannery et al. (2020)[314]; Fu et al. (2013)[315]; Fu et al. (2013)[316]; Fu et al. (2015)[317]; Fu et al. (2020)[318]; Gao et al. (2024)[319]; Glatman-Freedman et al. (2020)[320]; Grijalva et al. (2015)[321]; Halloran et al. (2003)[322]; Halloran et al. (2007)[323]; Hardelid et al. (2012)[324]; Helmeke et al. (2015)[325]; Hermann et al. (2015)[326]; Hood et al. (2023)[327]; Hu et al. (2021)[328]; Jain et al. (2013)[329]; Janjua et al. (2012)[330]; Joshi et al. (2009)[331]; Kafatos et al. (2013)[332]; Kang et al. (2019)[333]; Kao et al. (2020)[334]; Kelly et al. (2011)[335]; Kelly et al. (2011)[336]; Kim et al. (2012)[337]; Kim et al. (2022)[338]; Kimiya et al. (2018)[339]; Kittikraisak et al. (2016)[340]; Larrauri et al. (2011)[341]; Lee et al. (2024)[342]; Leung et al. (2017)[343]; Leung et al. (2018)[344]; Levy et al. (2014)[345]; Levy et al. (2014)[346]; Levy et al. (2015)[347]; Li-Kim-Moy et al. (2017)[348]; Luo et al. (2019)[349]; Maeda et al. (2004)[350]; Malosh et al. (2021)[351]; Marron et al. (2024)[352]; Matsumoto et al. (2021)[353]; McLean et al. (2015)[354]; Mi et al. (2024)[355]; Mohl et al. (2018)[356]; Mori et al. (2014)[357]; Murphy et al. (2024)[358]; Nakamura et al. (1966)[359]; Ng et al. (2013)[360]; Niang et al. (2021)[361]; Ohmit et al. (2013)[362]; Ohmit et al. (2014)[363]; Ohmit et al. (2015)[364]; Ohmit et al. (2016)[365]; Olson et al. (2022)[366]; Omer et al. (2022)[367]; Orellano et al. (2010)[368]; Pebody et al. (2013)[369]; Pebody et al. (2013)[370]; Pebody et al. (2015)[371]; Pebody et al. (2017)[372]; Pebody et al. (2020)[373]; Perez-Gimeno et al. (2024)[374]; Petrie et al. (2017)[375]; Powell et al. (2019)[376]; Powell et al. (2020)[377]; Price et al. (2023)[378]; Price et al. (2023)[378]; Puig-Ba et al. (2019)[379]; Qin et al. (2016)[380]; Rolfes et al. (2019)[381]; Sahni et al. (2023)[382]; Segaloff et al. (2019)[383]; Shinjoh et al. (2022)[384]; Shinjoh et al. (2023)[385]; Shinjoh et al. (2024)[386]; Simpson et al. (2020)[387]; Smolarchuk et al. (2024)[388]; Sominina et al. (2021)[389]; Stein et al. (2018)[390]; Stuurman et al. (2021)[391]; Stuurman et al. (2023)[392]; Su et al. (2015)[393]; Sugaya et al. (1994)[394]; Sugaya et al. (2016)[395]; Sugaya et al. (2018)[396]; Sullender et al. (2019)[397]; Sumner et al. (2024)[398]; Sun et al. (2025)[399]; Suzuki et al. (2014)[400]; Switzer et al. (2022)[401]; Tenforde et al. (2021)[402]; Tenforde et al. (2024)[403]; Teros-Jaakkola et al. (2019)[404]; Turner et al. (2014)[405]; Valdin et al. (2017)[406]; Wang et al. (2018)[407]; Wang et al. (2018)[408]; Wang et al. (2019)[409]; Widgren et al. (2013)[410]; Wu et al. (2018)[411]; Yamaguchi et al. (2010)[412]; Yang et al. (2024)[413]; Zeno et al. (2024)[414]; Zhang et al. (2017)[415]; Zhang et al. (2024)[18]; Zhu et al. (2024)[19]; Zimmerman et al. (2016)[416]

Continued on next page

©2025 Goldsmith JJ et al. *JAMA Network Open*.

eTable 5 Continued from previous page

**Reason**	**Excluded studies**
Wrong study type	Abraham et al. (2020)[417]; Ahmed et al. (2019)[418]; Ambrose et al. (2010)[419]; Amer et al. (2017)[420]; Ashkenazi et al. (2006)[421]; Bakkaloglu et al. (2016)[422]; Bandell et al. (2020)[423]; Baum et al. (2020)[424]; Bekkat-Berkani et al. (2016)[425]; Belongia et al. (2015)[426]; Belshe et al. (2000)[427]; Belshe et al. (2007)[428]; Belshe et al. (2008)[429]; Belshe et al. (2019)[430]; Campbell et al. (2019)[431]; Cantarutti et al. (2021)[432]; Carvalho et al. (2012)[433]; Chung et al. (2019)[434]; Collignon et al. (2015)[435]; Cowling et al. (2018)[436]; Cowling et al. (2019)[437]; D et al. (2013)[438]; Dierig et al. (2014)[439]; Divino et al. (2021)[440]; Domorazkova et al. (1981)[441]; Fowlkes et al. (2017)[442]; Fukushima et al. (2021)[443]; Hamrin et al. (2010)[444]; Herron et al. (1979)[445]; Homaira et al. (2020)[446]; Isaacs et al. (2016)[447]; Jennings et al. (1980)[448]; Jing-Xia et al. (2017)[449]; Karras et al. (2013)[450]; Keitel et al. (2002)[451]; Kittikraisak et al. (2015)[452]; Kolber et al. (2014)[453]; Ladva et al. (2019)[454]; Loeb et al. (2010)[455]; Longini et al. (2000)[456]; Luna et al. (2009)[457]; Maeda et al. (2002)[458]; Mahmud et al. (2013)[459]; McLean et al. (2017)[460]; Mendelman et al. (2001)[461]; Motaghi et al. (2024)[462]; Nicu et al. (1981)[463]; Nolan et al. (2014)[464]; Ochiai et al. (1986)[465]; Pelton et al. (2021)[466]; Petrie et al. (2016)[467]; Prevot et al. (2023)[327]; Rao et al. (2019)[468]; Regan et al. (2023)[469]; Rose et al. (1999)[470]; Sahni et al. (2023)[382]; Schaad et al. (2000)[471]; Seeborg et al. (2009)[472]; Shibata et al. (2015)[473]; Singh et al. (2010)[474]; Skowronski et al. (2010)[475]; Skowronski et al. (2019)[476]; Tkaczyszyn et al. (2020)[477]; Tsai et al. (2011)[478]; Uphoff et al. (2006)[479]; Vesikari et al. (2018)[480]; Wagner et al. (2021)[481]; Wat et al. (2008)[482]; Wijnans et al. (2010)[483]; de Paiva et al. (2001)[484]

End of table

©2025 Goldsmith JJ et al. *JAMA Network Open*.

eTable 6. PRISMA 2020 Checklist

Section and Topic	Item	Checklist Item	Location where item is reported
TITLE			
Title	1	Identify the report as a systematic review.	Page 1
ABSTRACT			
Abstract	2	See the PRISMA 2020 for Abstracts Checklist.	Page iii
INTRODUCTION			
Rationale	3	Describe the rationale for the review in the context of existing knowledge.	Pages 1-2
Objectives	4	Provide an explicit statement of the objective(s) or question(s) the review addresses.	Page 2
METHODS			
Eligibility Criteria	5	Specify the inclusion and exclusion criteria for the review and how studies were grouped for the syntheses	Pages 3-5, Appendix A.2
Information sources	6	Specify all databases, registers, websites, organisations, reference lists and other sources searched or consulted to identify studies. Specify the date when each source was last searched or consulted.	Page 4
Search strategy	7	Present the full search strategies for all databases, registers and websites, including any filters and limits used.	Appendix A.1
Selection process	8	Specify the methods used to decide whether a study met the inclusion criteria of the review, including how many reviewers screened each record and each report retrieved, whether they worked independently, and if applicable, details of automation tools used in the process.	Pages 3-4
Data collection process	9	Specify the methods used to collect data from reports, including how many reviewers collected data from each report, whether they worked independently, any processes for obtaining or confirming data from study investigators, and if applicable, details of automation tools used in the process.	Page 4
Data items	10a	List and define all outcomes for which data were sought. Specify whether all results that were compatible with each outcome domain in each study were sought (e.g. for all measures, time points, analyses), and if not, the methods used to decide which results to collect.	Appendix A.3, Page 6
	10b	List and define all other variables for which data were sought (e.g. participant and intervention characteristics, funding sources). Describe any assumptions made about any missing or unclear information.	Appendix A.3, Appendix A.4
Study risk of bias assessment	11	Specify the methods used to assess risk of bias in the included studies, including details of the tool(s) used, how many reviewers assessed each study and whether they worked independently, and if applicable, details of automation tools used in the process.	Page 4
Effect measures	12	Specify for each outcome the effect measure(s) (e.g. risk ratio, mean difference) used in the synthesis or presentation of results	Page 4

©2025 Goldsmith JJ et al. *JAMA Network Open*.

PRISMA 2020 Checklist, Cont'd.

Section and Topic	Item	Checklist Item	Location where item is reported
Synthesis methods	13a	Describe the processes used to decide which studies were eligible for each synthesis (e.g. tabulating the study intervention characteristics and comparing against the planned groups for each synthesis (item #5)).	Pages 5-6
	13b	Describe any methods required to prepare the data for presentation or synthesis, such as handling of missing summary statistics, or data conversions.	Pages 5-7
	13c	Describe any methods used to tabulate or visually display results of individual studies and syntheses.	Pages 5-7
	13d	Describe any methods used to synthesize results and provide a rationale for the choice(s). If meta-analysis was performed, describe the model(s), method(s) to identify the presence and extent of statistical heterogeneity, and software package(s) used.	Pages 6-7, Appendix A.5
	13e	Describe any methods used to explore possible causes of heterogeneity among study results (e.g. subgroup analysis, meta-regression).	Pages 5-7
	13f	Describe any sensitivity analyses conducted to assess robustness of the synthesized results.	Pages 5-7
Reporting bias assessment	14	Describe any methods used to assess risk of bias due to missing results in a synthesis (arising from reporting biases).	Page 7
Certainty assessment	15	Describe any methods used to assess certainty (or confidence) in the body of evidence for an outcome.	Pages 6-7

RESULTS

Section and Topic	Item	Checklist Item	Location where item is reported
Study selection	16a	Describe the results of the search and selection process, from the number of records identified in the search to the number of studies included in the review, ideally using a flow diagram.	Page 8, Figure A.1
	16b	Cite studies that might appear to meet the inclusion criteria, but which were excluded, and explain why they were excluded.	Table A.6
Study Characteristics	17	Cite each included study and present its characteristics.	Table 1, Pages 8-9, Figure 1
Risk of bias in studies	18	Present assessments of risk of bias for each included study.	Appendix A.7
Results of individual studies	19	For all outcomes, present, for each study: (a) summary statistics for each group (where appropriate) and (b) an effect estimate and its precision (e.g. confidence/credible interval), ideally using structured tables or plots.	Figures 2-3, Appendix A.6
Results of syntheses	20a	For each synthesis, briefly summarise the characteristics and risk of bias among contributing studies	Pages 8-9, P11
	20b	Present results of all statistical syntheses conducted. If meta-analysis was done, present for each the summary estimate and its precision (e.g. confidence/credible interval) and measures of statistical heterogeneity. If comparing groups, describe the direction of the effect.	Pages 9-11, Appendix A.6
	20c	Present results of all investigations of possible causes of heterogeneity among study results.	Figures 2-3, Appendix A.6
	20d	Present results of all sensitivity analyses conducted to assess the robustness of the synthesized results.	Appendix A.6.2

©2025 Goldsmith JJ et al. *JAMA Network Open*.

PRISMA 2020 Checklist, Cont'd.

Section and Topic	Item	Checklist Item	Location where item is reported
Reporting biases	21	Present assessments of risk of bias due to missing results (arising from reporting biases) for each synthesis assessed.	Appendix A.8
Certainty of evidence	22	Present assessments of certainty (or confidence) in the body of evidence for each outcome assessed.	Appendix A.7
DISCUSSION			
Discussion	23a	Provide a general interpretation of the results in the context of other evidence.	Pages 12-15
	23b	Discuss any limitations of the evidence included in the review.	Page 14
	23c	Discuss any limitations of the review processes used.	Page 14
	23d	Discuss implications of the results for practice, policy, and future research.	Pages 14-15
OTHER INFORMATION			
Registration and protocol	24a	Provide registration information for the review, including register name and registration number, or state that the review was not registered.	Page 3
	24b	Indicate where the review protocol can be accessed, or state that a protocol was not prepared.	Page 3
	24c	Describe and explain any amendments to information provided at registration or in the protocol.	Page 3
Support	25	Describe sources of financial or non-financial support for the review, and the role of the funders or sponsors in the review.	Page i
Competing interests	26	Declare any competing interests of review authors.	Page i
Availability of data, code and other materials	27	Report which of the following are publicly available and where they can be found: template data collection forms; data extracted from included studies; data used for all analyses; analytic code; any other materials used in the review.	Page i

eReferences

1. Jones-Gray E, Robinson EJ, Kucharski AJ, Fox A, and Sullivan SG. Does repeated influenza vaccination attenuate effectiveness? A systematic review and meta-analysis. Lancet Respir. Med. 2023 Jan; 11:27–44. DOI: 10.1016/S2213-2600(22)00266-1
2. Gilca R, Deceuninck G, De Serres G, et al. Effectiveness of pandemic H1N1 vaccine against influenza-related hospitalization in children. Pediatrics 2011; 128:1084–91. DOI: 10.1542/peds.2010-3492
3. Griffin MR, Monto AS, Belongia EA, et al. Effectiveness of non-adjuvanted pandemic influenza A vaccines for preventing pandemic influenza acute respiratory illness visits in 4 U.S. communities. PLoS One 2011 Aug; 6:e23085
4. Hadler JL, Baker TN, Papadouka V, France AM, Zimmerman C, Livingston KA, and Zucker JR. Effectiveness of 1 dose of 2009 influenza A (H1N1) vaccine at preventing hospitalization with pandemic H1N1 influenza in children aged 7 months-9 years. J. Infect. Dis 2012; 206:49–55. DOI: 10.1093/infdis/jis306
5. Mahmud S, Hammond G, Elliott L, et al. Effectiveness of the pandemic H1N1 influenza vaccines against laboratory-confirmed H1N1 infections: population-based case–control study. Vaccine 2011; 29:7975–81. DOI: https://doi.org/10.1016/j.vaccine.2011.08.068
6. Van Buynder PG, Dhaliwal JK, Van Buynder JL, Couturier C, Minville-Leblanc M, Garceau R, and Tremblay FW. Protective effect of single-dose adjuvanted pandemic influenza vaccine in children. Influenza Other Respir. Viruses 2010; 4:171–8. DOI: 10.1111/j.1750-2659.2010.00146.x
7. Page M, Higgins J, and JAC S. Chapter 13: Assessing risk of bias due to missing evidence in a meta-analysis [last updated August 2024]. Ed. by Higgins J, Thomas J, Chandler J, Cumpston M, Li T, Page M, and Welch V. Cochrane, 2024
8. Belshe RB, Gruber WC, Mendelman PM, et al. Efficacy of vaccination with live attenuated, cold-adapted, trivalent, intranasal influenza virus vaccine against a variant (A/Sydney) not contained in the vaccine. J. Pediatr. 2000; 136:168–75
9. Belshe RB and Gruber WC. Safety, efficacy and effectiveness of cold-adapted, live, attenuated, trivalent, intranasal influenza vaccine in adults and children. Philos. Trans. R. Soc. Lond. B. Biol. Sci. 2001; 356:1947–51
10. Belshe RB, Nichol KL, Black SB, et al. Safety, efficacy, and effectiveness of live, attenuated, cold-adapted influenza vaccine in an indicated population aged 5-49 years. Clin. Infect. Dis. 2004; 39:920–7
11. Block SL, Toback SL, Yi T, and Ambrose CS. Efficacy of a single dose of live attenuated influenza vaccine in previously unvaccinated children: a post hoc analysis of three studies of children aged 2 to 6 years. Clin. Ther. 2009; 31:2140–7. DOI: https://doi.org/10.1016/j.clinthera.2009.09.014
12. Chung JR, Flannery B, Gaglani M, et al. Effect of influenza vaccine priming on current season vaccine effectiveness among children and adolescents, US FLU VE Network 2014-2015 through 2017-2018. Open Forum Infect. Dis. 2019; 6:S27. DOI: 10.1093/ofid/ofz359.059
13. Chung JR, Price AM, Zimmerman RK, et al. Influenza vaccine effectiveness against medically attended outpatient illness, United States, 2023-24 season. Clin. Infect. Dis. 2025. DOI: 10.1093/cid/ciae658
14. Nakayama T, Hayashi T, Makino K, and Oe K. The efficacy and safety of a quadrivalent live attenuated influenza nasal vaccine in Japanese children: A phase 3, randomized, placebo-controlled study. J. Infect. Chemother. 2024. DOI: 10.1016/j.jiac.2024.06.023
15. Rigamonti V, Torri V, Morris SK, et al. Real-world effectiveness of influenza vaccination in preventing influenza and influenza-like illness in children. Vaccine 2025; 53:126946. DOI: 10.1016/j.vaccine.2025.126946
16. Vasil'eva RI, Liamtseva GA, Riazantseva TG, Oleinikova EV, and Sosunov AV. [Assessment of the prophylactic effectiveness of an inactivated influenza vaccine by immunizing schoolchildren in the springtime]. Zh. Mikrobiol. Epidemiol. Immunobiol. 1986 :10–14
17. Whitaker H, Findlay B, Zitha J, et al. Interim 2023/2024 season influenza vaccine effectiveness in primary and secondary care in the United Kingdom. Influenza Other Respir. Viruses 2024; 18:e13284. DOI: 10.1111/irv.13284
18. Zhang J, Zhang L, Li J, et al. Moderate effectiveness of influenza vaccine in outpatient settings: A test-negative study in Beijing, China, 2023/24 season. Vaccine 2024; 46:126662. DOI: 10.1016/j.vaccine.2024.126662

19. Zhu L, Han Y, Lu J, et al. Evaluation of influenza vaccine effectiveness from 2021 to 2024: a Guangdong-Based test-negative case-control study. Vaccines 2024; 13. DOI: 10.3390/vaccines13010004
20. Allison MA, Daley MF, Crane LA, et al. Influenza vaccine effectiveness in healthy 6- to 21-month-old children during the 2003-2004 season. J. Pediatr. 2006; 149:755–62
21. Boikos C, Imran M, Nguyen VH, Ducruet T, Sylvester GC, and Mansi JA. Effectiveness of the cell-derived inactivated quadrivalent influenza vaccine in individuals at high risk of influenza complications in the 2018-2019 United States influenza season. Open Forum Infect. Dis. 2021; 8:ofab167. DOI: 10.1093/ofid/ofab167
22. Carrat F, Tachet A, Rouzioux C, Housset B, and Valleron AJ. Field investigation of influenza vaccine effectiveness on morbidity. Vaccine 1998; 16:893–8
23. Chen CM, Chen HJ, Chen WS, Lin CC, Hsu CC, and Hsu YH. Clinical effectiveness of influenza vaccination in patients with rheumatoid arthritis. Int. J. Rheum. Dis. 2018; 21:1246–53. DOI: 10.1111/1756-185X.13322
24. Chumakov MP, Malyshkina LP, Mart'ianova LI, Marinina VP, and Mel'nikova SK. [Immunogenicity and the protective effectiveness of the subunit trivalent influenza vaccine Grippovac]. Zh. Mikrobiol. Epidemiol. Immunobiol. 1986 :44–9
25. Divino V, Krishnarajah G, Pelton SI, Mould-Quevedo J, Anupindi VR, DeKoven M, and Postma MJ. A real-world study evaluating the relative vaccine effectiveness of a cell-based quadrivalent influenza vaccine compared to egg-based quadrivalent influenza vaccine in the US during the 2017-18 influenza season. Vaccine 2020; 38:6334–43. DOI: 10.1016/j.vaccine.2020.07.023
26. Glenn DA, Pate V, Zee J, et al. Influenza vaccine administration and effectiveness among children and adults with Glomerular disease. Kidney Int. Rep. 2024; 9:257EP–265. DOI: 10.1016/j.ekir.2023.10.031
27. He Q, Xu J, Chen X, et al. Effectiveness of seasonal influenza vaccine against clinically diagnosed influenza over 2 consecutive seasons in children in Guangzhou, China: a matched case-control study. Hum. Vaccin. Immunother. 2013; 9:1720–4. DOI: 10.4161/hv.24980
28. Hoberman A, Greenberg DP, Paradise JL, et al. Effectiveness of inactivated influenza vaccine in preventing acute otitis media in young children: a randomized controlled trial. JAMA 2003; 290:1608–16
29. Hurwitz ES, Haber M, Chang A, et al. Studies of the 1996-1997 inactivated influenza vaccine among children attending day care: immunologic response, protection against infection, and clinical effectiveness. J. Infect. Dis 2000; 182:1218–21
30. Imran M, Ortiz JR, McLean HQ, et al. Relative effectiveness of cell-based versus egg-based quadrivalent influenza vaccines in children and adolescents in the United States during the 2019-2020 influenza season. Pediatr. Infect. Dis. J. 2022; 41:769–74. DOI: 10.1097/INF.0000000000003620
31. Jick H and Hagberg KW. Effectiveness of influenza vaccination in the United Kingdom, 1996-2007. Pharmacotherapy 2010; 30:1199–206. DOI: 10.1592/phco.30.12.1199
32. Krishnarajah G, Divino V, Postma MJ, Pelton SI, Anupindi VR, Dekoven M, and Mould-Quevedo J. Clinical and economic outcomes associated with cell-based quadrivalent influenza vaccine vs. Standard-dose egg-based quadrivalent influenza vaccines during the 2018-19 influenza season in the United States. Vaccines 2021; 9:1–17. DOI: 10.3390/vaccines9020080
33. Kulkarni PS, Agarkhedkar S, Lalwani S, et al. Effectiveness of an Indian-made attenuated influenza A(H1N1)pdm 2009 vaccine: a case control study. Hum. Vaccin. Immunother. 2014; 10:566–71
34. Matsuda A, Asayama K, Obara T, Yagi N, and Ohkubo T. Effectiveness of influenza vaccination among children in satellite cities of a metropolitan area in Tokyo, Japan during the 2014/2015-2018/2019 season. Tohoku J. Exp. Med. 2022; 258:69–78. DOI: 10.1620/tjem.2022.J057
35. Okuno Y and Nakamura K. Prophylactic effectiveness of live influenza vaccine in 1965. 1966; 9:89–95
36. Petrilli FL, Crovari P, and Badolati G. A2/Hong Kong influenza in Liguria. II. Vaccination. Giornale di Igiene Medicina Preventiva 1971; 12:209–37
37. Piedra PA, Gaglani MJ, Kozinetz CA, et al. Trivalent live attenuated intranasal influenza vaccine administered during the 2003-2004 influenza type A (H3N2) outbreak provided immediate, direct, and indirect protection in children. Pediatrics 2007; 120:553–64
38. Ritzwoller DP, Bridges CB, Shetterly S, Yamasaki K, Kolczak M, and France EK. Effectiveness of the 2003-2004 influenza vaccine among children 6 months to 8 years of age, with 1 vs 2 doses. Pediatrics 2005; 116:153–9

39. Rudenko LG, Lonskaya NI, Klimov AI, Vasilieva RI, and Ramirez A. Clinical and epidemiological evaluation of a live, cold-adapted influenza vaccine for 3-14-year-olds. Bulletin of the World Health Organization 1996; 74:77–84
40. Rudenko LG, Vasil'eva RI, Ismagulov AT, et al. [Prophylactic effectiveness of a live recombinant influenza type A vaccine in immunizing children aged 3-14 years]. Vopr. Virusol. 1996; 41:37–9
41. Shibata N, Kimura S, Hoshino T, Takeuchi M, and Urushihara H. Effectiveness of influenza vaccination for children in Japan: four-year observational study using a large-scale claims database. Vaccine 2018; 36:2809–15. DOI: 10.1016/j.vaccine.2018.03.082
42. Shibata N, Kimura S, Hoshino T, and Urushihara H. Influenza vaccination effectiveness for people aged under 65 years in Japan, 2013/2014 season: application of a doubly robust method to a large-scale, real-world dataset. BMC Infect. Dis. 2019; 19:586. DOI: 10.1186/s12879-019-4186-x
43. Simpson CR, Ritchie LD, Robertson C, Sheikh A, and McMenamin J. Effectiveness of H1N1 vaccine for the prevention of pandemic influenza in Scotland, UK: a retrospective observational cohort study. Lancet Infect. Dis. 2012; 12:696–702. DOI: 10.1016/S1473-3099(12)70133-0
44. Uchida M, Kaneko M, Hidaka Y, et al. Effectiveness of vaccination and wearing masks on seasonal influenza in Matsumoto City, Japan, in the 2014/2015 season: an observational study among all elementary schoolchildren. Prev. Med. reports 2017; 5:86–91
45. Yin JK, Lahra MM, Iskander M, et al. Pilot study of influenza vaccine effectiveness in urban Australian children attending childcare. J. Paediatr. Child Health. 2011; 47:857–62. DOI: 10.1111/j.1440-1754.2011.02098.x
46. Yokomichi H, Kojima R, Horiuchi S, et al. Effectiveness of influenza vaccination in infants and toddlers with and without prior infection history: The Japan Environment and Children's Study. Vaccine 2021; 39:1800–4. DOI: 10.1016/j.vaccine.2021.02.044
47. Yokomichi H, Mochizuki M, Horiuchi S, et al. Association of influenza vaccination or influenza virus infection history with subsequent infection risk among children: the Japan Environment and Children's Study (JECS). Prev. Med. 2023; 173:107599. DOI: 10.1016/j.ypmed.2023.107599
48. Alfelali M, Barasheed O, Koul P, et al. Influenza vaccine effectiveness among Hajj pilgrims: a test-negative case-control analysis of data from different Hajj years. Expert Rev. Vaccines 2019; 18:1103–14. DOI: 10.1080/14760584.2019.1646130
49. Awadalla ME, Alkadi H, Alarjani M, et al. Moderately low effectiveness of the influenza quadrivalent vaccine: potential mismatch between circulating strains and vaccine strains. Vaccines 2023; 11:1050. DOI: 10.3390/vaccines11061050
50. Balasubramani GK, Nowalk MP, Sax TM, et al. Influenza vaccine effectiveness among outpatients in the US Influenza Vaccine Effectiveness Network by study site 2011-2016. Influenza Other Respir. Viruses 2020; 14:380–90. DOI: 10.1111/irv.12741
51. Balasubramani GK, Zimmerman RK, Eng H, Lyons J, Clarke L, and Nowalk MP. Comparison of local influenza vaccine effectiveness using two methods. Vaccine 2021; 39:1283–9. DOI: 10.1016/j.vaccine.2021.01.013
52. Baselga-Moreno V, Trushakova S, McNeil S, et al. Influenza epidemiology and influenza vaccine effectiveness during the 2016-2017 season in the Global Influenza Hospital Surveillance Network (GIHSN). BMC Public Health 2019; 19:487. DOI: 10.1186/s12889-019-6713-5
53. Bateman AC, Kieke BA, Irving SA, Meece JK, Shay DK, and Belongia EA. Effectiveness of monovalent 2009 pandemic influenza A virus subtype H1N1 and 2010-2011 trivalent inactivated influenza vaccines in Wisconsin during the 2010-2011 influenza season. J. Infect. Dis 2013; 207:1262–9. DOI: 10.1093/infdis/jit020
54. Bellino S, Bella A, Puzelli S, et al. Moderate influenza vaccine effectiveness against A(H1N1)pdm09 virus, and low effectiveness against A(H3N2) subtype, 2018/19 season in Italy. Expert Rev. Vaccines 2019; 18:1201–9. DOI: 10.1080/14760584.2019.1688151
55. Belongia EA, Kieke BA, Donahue JG, et al. Effectiveness of inactivated influenza vaccines varied substantially with antigenic match from the 2004-2005 season to the 2006-2007 season. J. Infect. Dis 2009; 199:159–67. DOI: 10.1086/595861
56. Blanquart F, Vieillefond V, Visseaux B, et al. Influenza vaccine effectiveness against detected infection in the community, France, October 2024 to February 2025. Euro. Surveill. 2025; 30. DOI: 10.2807/1560-7917.ES.2025.30.7.2500074
57. Blyth CC, Cheng AC, Finucane C, et al. The effectiveness of influenza vaccination in preventing hospitalisation in children in Western Australia. Vaccine 2015; 33:7239–44. DOI: 10.1016/j.vaccine.2015.10.122

58. Boddington NL, Warburton F, Zhao H, Andrews N, Ellis J, Donati M, and Pebody RG. Influenza vaccine effectiveness against hospitalisation due to laboratory-confirmed influenza in children in England in the 2015-2016 influenza season - a test-negative case-control study. Epidemiol. Infect. 2019; 147:e201. DOI: 10.1017/S0950268819000876
59. Bruxvoort KJ, Luo Y, Ackerson B, Tanenbaum HC, Sy LS, Gandhi A, and Tseng HF. Comparison of vaccine effectiveness against influenza hospitalization of cell-based and egg-based influenza vaccines, 2017-2018. Vaccine 2019; 37:5807–11. DOI: 10.1016/j.vaccine.2019.08.024
60. Cantarutti A, Barbieri E, Didone F, Scamarcia A, Giaquinto C, and Corrao G. Influenza vaccination effectiveness in paediatric 'healthy' patients: a population-based study in Italy. Vaccines 2022; 10. DOI: 10.3390/vaccines10040582
61. Carbo Malonda RM, Gonzalez Moran F, Vanaclocha Luna H, Martin-Sierra Balibrea M, Guiral Rodrigo S, Perez Perez E, and Castellanos Martinez T. [Pandemic influenza surveillance in the valencian community and seasonal influenza vaccines]. Rev. Esp. Salud Publica 2010; 84:623–33
62. Carville KS, Grant KA, Sullivan SG, et al. Understanding influenza vaccine protection in the community: an assessment of the 2013 influenza season in Victoria, Australia. Vaccine 2015; 33:341–5. DOI: 10.1016/j.vaccine.2014.11.019
63. Castilla J, Arregui L, Baleztena J, et al. [Incidence of influenza and influenza vaccine effectiveness in the 2004-2005 season]. An. Sist. Sanit. Navar. 2006; 29:97–106
64. Castilla J, Moran J, Martinez-Artola V, et al. Effectiveness of the monovalent influenza A(H1N1)2009 vaccine in Navarre, Spain, 2009-2010: cohort and case-control study. Vaccine 2011; 29:5919–24. DOI: 10.1016/j.vaccine.2011.06.063
65. Castilla J, Martinez-Artola V, Salcedo E, et al. Vaccine effectiveness in preventing influenza hospitalizations in Navarre, Spain, 2010-2011: cohort and case-control study. Vaccine 2012; 30:195–200. DOI: 10.1016/j.vaccine.2011.11.024
66. Castilla J, Martinez-Baz I, Martinez-Artola V, et al. Decline in influenza vaccine effectiveness with time after vaccination, Navarre, Spain, season 2011/12. Euro. Surveill. 2013; 18
67. Castilla J, Godoy P, Dominguez A, et al. Influenza vaccine effectiveness in preventing outpatient, inpatient, and severe cases of laboratory-confirmed influenza. Clin. Infect. Dis. 2013; 57:167–75. DOI: 10.1093/cid/cit194
68. Castilla J, Navascues A, Fernandez-Alonso M, et al. Effectiveness of subunit influenza vaccination in the 2014-2015 season and residual effect of split vaccination in previous seasons. Vaccine 2016; 34:1350–7. DOI: 10.1016/j.vaccine.2016.01.054
69. Castilla J, Navascues A, Fernandez-Alonso M, et al. Effects of previous episodes of influenza and vaccination in preventing laboratory-confirmed influenza in Navarre, Spain, 2013/14 season. Euro. Surveill. 2016; 20. DOI: 10.2807/1560-7917.ES.2016.21.22.30243
70. Castilla J, Portillo ME, Casado I, et al. Effectiveness of the current and prior influenza vaccinations in northern Spain, 2018-2019. Vaccine 2020; 38:1925–32. DOI: 10.1016/j.vaccine.2020.01.028
71. Cervi V, Bizzego S, Piacentini I, Cazzola GA, Orandini A, Donatelli I, and Grandolfo M. Evaluation of the efficacy of influenza vaccine in elderly subjects and in children with cystic fibrosis. Microbiologia Medica 1997; 12:402–4
72. Chan YWD, Wong ML, Au KW, and Chuang SK. Seasonal influenza vaccine effectiveness at primary care level, Hong Kong SAR, 2017/2018 winter. Hum. Vaccin. Immunother. 2019; 15:97–101. DOI: 10.1080/21645515.2018.1514222
73. Y.-W. C, M.-L. W, F.-Y. K, A.K.-W. A, E.C.-M. L, and S.-K. C. The effect of seasonal influenza vaccine on medically-attended influenza and non-influenza respiratory viruses infections at primary care level, Hong Kong SAR, 2017/18 to 2019/20. Vaccine 2021; 39:3372–8. DOI: 10.1016/j.vaccine.2021.04.059
74. Cheng AC, Kotsimbos T, Kelly HA, et al. Effectiveness of H1N1/09 monovalent and trivalent influenza vaccines against hospitalization with laboratory-confirmed H1N1/09 influenza in Australia: a test-negative case control study. Vaccine 2011; 29:7320–5. DOI: 10.1016/j.vaccine.2011.07.087
75. Cheng AC, Brown S, Waterer G, et al. Influenza epidemiology, vaccine coverage and vaccine effectiveness in sentinel Australian hospitals in 2012: the Influenza Complications Alert Network (FluCAN). Commun Dis Intell Q Rep 2013; 37:246–52
76. Cheng AC, Dwyer DE, Holmes M, et al. Influenza epidemiology, vaccine coverage and vaccine effectiveness in sentinel Australian hospitals in 2013: the Influenza Complications Alert Network. Commun Dis Intell Q Rep 2014; 38:143–9

77. Cheng AC, Holmes M, Dwyer DE, et al. Influenza epidemiology in patients admitted to sentinel Australian hospitals in 2017: the Influenza Complications Alert Network (FluCAN). Commun. Dis. Intell. (2018) 2019; 43. DOI: 10.33321/cdi.2019.43.39
78. Cheng AC, Dwyer DE, Holmes M, et al. Influenza epidemiology in patients admitted to sentinel Australian hospitals in 2019: the Influenza Complications Alert Network (FluCAN). Commun. Dis. Intell. (2018) 2022; 46. DOI: 10.33321/cdi.2022.46.14
79. Chung JR, Flannery B, Thompson MG, et al. Seasonal effectiveness of live attenuated and inactivated influenza vaccine. Pediatrics 2016; 137:e20153279. DOI: 10.1542/peds.2015-3279
80. Chung J, Shirk P, Gaglani M, et al. Late-season influenza vaccine effectiveness against medically attended outpatient illness, United States, December 2022-April 2023. Influenza Other Respir. Viruses 2024; 18:e13342. DOI: 10.1111/irv.13342
81. Chung H, Campitelli MA, Buchan SA, et al. Measuring waning protection from seasonal influenza vaccination during nine influenza seasons, Ontario, Canada, 2010/11 to 2018/19. Euro. Surveill. 2024; 29:2300239. DOI: 10.2807/1560-7917.ES.2024.29.8.2300239
82. Colucci ME, Veronesi L, Bracchi MT, et al. On field vaccine effectiveness in three periods of 2018/2019 influenza season in Emilia-Romagna Region. Acta Biomed. 2019; 90:21–7. DOI: 10.23750/abm.v90i9-S.8699
83. Costantino C, Mazzucco W, Graziano G, Maida CM, Vitale F, and Tramuto F. Mid-term estimates of influenza vaccine effectiveness against the A(H1N1)pdm09 prevalent circulating subtype in the 2023/24 season: data from the Sicilian RespiVirNet Surveillance System. Vaccines 2024; 12:305. DOI: 10.3390/vaccines12030305
84. Cowling BJ, Ng S, Ma ESK, et al. Protective efficacy of seasonal influenza vaccination against seasonal and pandemic influenza virus infection during 2009 in Hong Kong. Clin. Infect. Dis. 2010; 51:1370–9. DOI: 10.1086/657311
85. Cowling BJ, Ng S, Ma ESK, et al. Protective efficacy against pandemic influenza of seasonal influenza vaccination in children in Hong Kong: a randomized controlled trial. Clin. Infect. Dis. 2012; 55:695–702. DOI: 10.1093/cid/cis518
86. Cowling BJ, Chan KH, Feng S, Chan ELY, Lo JYC, Peiris JSM, and Chiu SS. The effectiveness of influenza vaccination in preventing hospitalizations in children in Hong Kong, 2009-2013. Vaccine 2014; 32:5278–84. DOI: 10.1016/j.vaccine.2014.07.084
87. Darvishian M, Dijkstra F, Doorn E van, et al. Influenza vaccine effectiveness in the Netherlands from 2003/2004 through 2013/2014: the importance of circulating influenza virus types and subtypes. PLoS One 2017; 12:e0169528. DOI: 10.1371/journal.pone.0169528
88. DeMarcus LS, Parms TA, and Thervil JW. The DoD Global, Laboratory-based, Influenza Surveillance Program: summary for the 2013-2014 influenza season. MSMR 2016; 23:2–5
89. DeMarcus L, Shoubaki L, and Federinko S. Comparing influenza vaccine effectiveness between cell-derived and egg-derived vaccines, 2017-2018 influenza season. Vaccine 2019; 37:4015–21. DOI: 10.1016/j.vaccine.2019.06.004
90. Dogliani M, Fidelio T, Scalzo B, Iacono G, Deabate MC, Bagatella M, and Saracco B. [Effectiveness of influenza vaccination in patients undergoing regular dialysis treatments]. Minerva Urol. Nefrol. 1997; 49:121–4
91. Dominguez A, Castilla J, Godoy P, et al. Effectiveness of pandemic and seasonal influenza vaccines in preventing pandemic influenza-associated hospitalization. Vaccine 2012; 30:5644–50
92. Domnich A, Orsi A, Signori A, et al. Waning intra-season vaccine effectiveness against influenza A(H3N2) underlines the need for more durable protection. Expert Rev. Vaccines 2024; 23:380EP–388. DOI: 10.1080/14760584.2024.2331073
93. Edwards KM, Dupont WD, Westrich MK, Plummer WDJ, Palmer PS, and Wright PF. A randomized controlled trial of cold-adapted and inactivated vaccines for the prevention of influenza A disease. J. Infect. Dis 1994; 169:68–76
94. El'shina GA, Gorbunov MA, Bektimirov TA, et al. [The evaluation of the reactogenicity, harmlessness and prophylactic efficacy of Grippol trivalent polymer-subunit influenza vaccine administered to schoolchildren]. Zh. Mikrobiol. Epidemiol. Immunobiol. 2000 :50–4
95. H.-D. E, Krause TG, Hviid A, Simonsen J, and Molbak K. Effectiveness of vaccine against pandemic influenza A/H1N1 among people with underlying chronic diseases: cohort study, Denmark, 2009-10. BMJ 2012; 344:d7901. DOI: 10.1136/bmj.d7901
96. Englund H, Campe H, and Hautmann W. Effectiveness of trivalent and monovalent influenza vaccines against laboratory-confirmed influenza infection in persons with medically attended influenza-like

illness in Bavaria, Germany, 2010/2011 season. Epidemiol. Infect. 2013; 141:1807–15. DOI: 10.1017/S0950268812002282
97. Falchi A, Souty C, Grisoni ML, et al. Field seasonal influenza vaccine effectiveness: evaluation of the screening method using different sources of data during the 2010/2011 French influenza season. Hum. Vaccin. Immunother. 2013; 9:2453–9
98. Ferdinands JM, Olsho LEW, Agan AA, et al. Effectiveness of influenza vaccine against life-threatening RT-PCR-confirmed influenza illness in US children, 2010-2012. J. Infect. Dis 2014; 210:674–83. DOI: 10.1093/infdis/jiu185
99. Fielding JE, Grant KA, Garcia K, and Kelly HA. Effectiveness of seasonal influenza vaccine against pandemic (H1N1) 2009 virus, Australia, 2010. Emerg. Infect. Dis. 2011; 17:1181–7. DOI: 10.3201/eid1707.101959
100. Fielding JE, Grant KA, Papadakis G, and Kelly HA. Estimation of type- and subtype-specific influenza vaccine effectiveness in Victoria, Australia using a test negative case control method, 2007-2008. BMC Infect. Dis. 2011; 11:170. DOI: 10.1186/1471-2334-11-170
101. Fielding JE, Grant KA, Tran T, and Kelly HA. Moderate influenza vaccine effectiveness in Victoria, Australia, 2011. Euro. Surveill. 2012; 17
102. Fielding JE, Levy A, Chilver MB, et al. Effectiveness of seasonal influenza vaccine in Australia, 2015: An epidemiological, antigenic and phylogenetic assessment. Vaccine 2016; 34:4905–12. DOI: 10.1016/j.vaccine.2016.08.067
103. Flannery B, Smith C, Garten RJ, et al. Influence of birth cohort on effectiveness of 2015-2016 influenza vaccine against medically attended illness due to 2009 pandemic influenza A(H1N1) virus in the United States. J. Infect. Dis 2018; 218:189–96. DOI: 10.1093/infdis/jix634
104. Fleming DM, Andrews NJ, Ellis JS, et al. Estimating influenza vaccine effectiveness using routinely collected laboratory data. J. Epidemiol. Community Health 2010; 64:1062–7. DOI: 10.1136/jech.2009.093450
105. Frutos AM, Price AM, Harker E, et al. Interim estimates of 2023-24 seasonal influenza vaccine effectiveness - United States. MMWR Morb. Mortal Wkly. Rep. 2024; 73:168EP–174. DOI: 10.15585/mmwr.mm7308a3
106. Frutos AM, Cleary S, Reeves EL, et al. Interim estimates of 2024-2025 seasonal influenza vaccine effectiveness - four vaccine effectiveness networks, United States, October 2024-February 2025. MMWR Morb. Mortal Wkly. Rep. 2025; 74:83EP–90. DOI: 10.15585/mmwr.mm7406a2
107. Gaglani M, Vasudevan A, Raiyani C, et al. Effectiveness of Trivalent and Quadrivalent Inactivated Vaccines against Influenza B in the United States, 2011-2012 to 2016-2017. Clin. Infect. Dis. 2021; 72:1147–57. DOI: 10.1093/cid/ciaa102
108. Galtier F, Loulergue P, Vanhems P, et al. Influenza in patients with diabetes and obesity: Vaccine effectiveness against hospitalised influenza and complications after hospitalised influenza-like illness. Diabetologia 2015; 58:S157–S158. DOI: 10.1007/s00125-015-3687-4
109. Garcia-Garcia L, Valdespino-Gomez JL, Lazcano-Ponce E, et al. Partial protection of seasonal trivalent inactivated vaccine against novel pandemic influenza A/H1N1 2009: case-control study in Mexico City. BMJ 2009; 339:b3928. DOI: 10.1136/bmj.b3928
110. Gattas VL, Cardoso MRA, Mondini G, Machado CM, and Luna EJA. Effectiveness of influenza vaccination of schoolchildren in the city of Sao Paulo, Brazil, 2009. Influenza Other Respir. Viruses 2015; 9:323–30. DOI: 10.1111/irv.12328
111. Gharpure R, Regan AK, Nogareda F, et al. Effectiveness of 2023 southern hemisphere influenza vaccines against severe influenza-associated illness: pooled estimates from eight countries using the test-negative design. Lancet Glob. Health 2025; 13:e203EP–e211. DOI: 10.1016/S2214-109X{\%}2824{\%}2900473-X
112. Gherasim A, Pozo F, Mateo S de, et al. Waning protection of influenza vaccine against mild laboratory confirmed influenza A(H3N2) and B in Spain, season 2014-15. Vaccine 2016; 34:2371–7. DOI: 10.1016/j.vaccine.2016.03.035
113. Guzman Herrador BR, Aavitsland P, Feiring B, Riise Bergsaker MA, and Borgen K. Usefulness of health registries when estimating vaccine effectiveness during the influenza A(H1N1)pdm09 pandemic in Norway. BMC Infect. Dis. 2012; 12:63. DOI: 10.1186/1471-2334-12-63
114. Hallmann-Szelinska E, Cieslak K, Szymanski K, Kowalczyk D, Korczynska MR, Paradowska-Stankiewicz I, and Brydak LB. Detection of influenza in the epidemic season 2016/2017 based on i-move+ project. Adv. Exp. Med. Biol. 2018; 1114:77–82. DOI: 10.1007/5584{_}2018{_}230

115. Hardelid P, Fleming DM, McMenamin J, et al. Effectiveness of pandemic and seasonal influenza vaccine in preventing pandemic influenza A(H1N1)2009 infection in England and Scotland 2009-2010. Euro. Surveill. 2011; 16
116. Hekimoglu CH, Emek M, Avci E, Topal S, Demiroz M, and Ergor G. Seasonal influenza vaccine effectiveness in preventing laboratory confirmed influenza in 2014-2015 season in Turkey: a test-negative case control study. Balkan Med. J. 2018; 35:77–83. DOI: 10.4274/balkanmedj.2017.0487
117. Hu W, Gruner WE, DeMarcus LS, et al. Influenza surveillance trends and influenza vaccine effectiveness among Department of Defense beneficiaries during the 2019-2020 influenza season. MSMR 2021; 28:2–8
118. Jackson ML, Chung JR, Jackson LA, et al. Influenza vaccine effectiveness in the United States during the 2015-2016 season. N. Engl. J. Med. 2017; 377:534–43. DOI: 10.1056/NEJMoa1700153
119. Jackson ML, Ferdinands J, Nowalk MP, et al. Differences between frequentist and bayesian inference in routine surveillance for influenza vaccine effectiveness: a test-negative case-control study. BMC Public Health 2021; 21:516. DOI: 10.1186/s12889-021-10543-z
120. Jimenez-Jorge S, Savulescu C, Pozo F, Mateo S de, Casas I, Ledesma J, and Larrauri A. Effectiveness of the 2010-11 seasonal trivalent influenza vaccine in Spain: cycEVA study. Vaccine 2012; 30:3595–602. DOI: 10.1016/j.vaccine.2012.03.048
121. Jimenez-Jorge S, Mateo S de, Delgado-Sanz C, et al. Effectiveness of influenza vaccine against laboratory-confirmed influenza, in the late 2011-2012 season in Spain, among population targeted for vaccination. BMC Infect. Dis. 2013; 13:441. DOI: 10.1186/1471-2334-13-441
122. Jimenez-Jorge S, Mateo S de, Delgado-Sanz C, et al. Estimating influenza vaccine effectiveness in Spain using sentinel surveillance data. Euro. Surveill. 2015; 20
123. Joshi AY, Iyer VN, Hartz MF, Patel AM, and Li JT. Effectiveness of trivalent inactivated influenza vaccine in influenza-related hospitalization in children: a case-control study. Allergy Asthma Proc. 2012; 33:23–7. DOI: 10.2500/aap.2012.33.3513
124. Kamada M, Nagai T, Kumagai T, et al. Efficacy of inactivated trivalent influenza vaccine in alleviating the febrile illness of culture-confirmed influenza in children in the 2000-2001 influenza season. Vaccine 2006; 24:3618–23
125. Kavanagh K, Robertson C, and McMenamin J. Assessment of the variability in influenza A(H1N1) vaccine effectiveness estimates dependent on outcome and methodological approach. PLoS One 2011; 6:e28743. DOI: 10.1371/journal.pone.0028743
126. Kavanagh K, Robertson C, and McMenamin J. Estimates of influenza vaccine effectiveness in primary care in Scotland vary with clinical or laboratory endpoint and method–experience across the 2010/11 season. Vaccine 2013; 31:4556–63. DOI: 10.1016/j.vaccine.2013.07.056
127. Kawai N, Ikematsu H, Iwaki N, Satoh I, Kawashima T, Tsuchimoto T, and Kashiwagi S. A prospective, Internet-based study of the effectiveness and safety of influenza vaccination in the 2001-2002 influenza season. Vaccine 2003; 21:4507–13
128. Kelly H, Carville K, Grant K, Jacoby P, Tran T, and Barr I. Estimation of influenza vaccine effectiveness from routine surveillance data. PLoS One 2009; 4:e5079. DOI: 10.1371/journal.pone.0005079
129. Kelly HA, Lane C, and Cheng AC. Influenza vaccine effectiveness in general practice and in hospital patients in Victoria, 2011-2013. Med. J. Aust. 2016; 204:76
130. Kersellius GD, Gruner WE, Fries AC, DeMarcus LS, and Robbins AS. Respiratory pathogen surveillance trends and influenza vaccine effectiveness estimates for the 2018-2019 season among Department of Defense beneficiaries. MSMR 2020; 27:17–23
131. Kim S, Chuang ESY, Sabaiduc S, et al. Influenza vaccine effectiveness against A(H3N2) during the delayed 2021/22 epidemic in Canada. Euro. Surveill. 2022; 27:2200720. DOI: 10.2807/1560-7917.ES.2022.27.38.2200720
132. King JP, Nguyen HQ, Kiniry EL, et al. Elevated body mass index is not significantly associated with reduced influenza vaccine effectiveness. Sci. Rep. 2024; 14:21466. DOI: 10.1038/s41598-024-72081-z
133. Kissling E, Valenciano M, Cohen JM, et al. I-MOVE multi-centre case control study 2010-11: overall and stratified estimates of influenza vaccine effectiveness in Europe. PLoS One 2011; 6:e27622. DOI: 10.1371/journal.pone.0027622
134. Kissling E, Valenciano M, Larrauri A, et al. Low and decreasing vaccine effectiveness against influenza A(H3) in 2011/12 among vaccination target groups in Europe: results from the I-MOVE multicentre case-control study. Euro. Surveill. 2013; 18

135. Kissling E, Valenciano M, Buchholz U, et al. Influenza vaccine effectiveness estimates in Europe in a season with three influenza type/subtypes circulating: the I-MOVE multicentre case-control study, influenza season 2012/13. Euro. Surveill. 2014; 19
136. Kissling E, Valenciano M, Pozo F, et al. 2015/16 I-MOVE/I-MOVE+ multicentre case-control study in Europe: Moderate vaccine effectiveness estimates against influenza A(H1N1)pdm09 and low estimates against lineage-mismatched influenza B among children. Influenza Other Respir. Viruses 2018; 12:423–37. DOI: 10.1111/irv.12520
137. Kissling E, Pozo F, Buda S, et al. Effectiveness of influenza vaccine against influenza A in Europe in seasons of different A(H1N1)pdm09 and the same A(H3N2) vaccine components (2016-17 and 2017-18). Vaccine: X 2019; 3:100042. DOI: 10.1016/j.jvacx.2019.100042
138. Kissling E, Pozo F, Buda S, et al. Low 2018/19 vaccine effectiveness against influenza A(H3N2) among 15-64-year-olds in Europe: exploration by birth cohort. Euro. Surveill. 2019; 24. DOI: 10.2807/1560-7917.ES.2019.24.48.1900604
139. Kissling E, Pozo F, Martinez-Baz I, et al. Influenza vaccine effectiveness against influenza A subtypes in Europe: Results from the 2021-2022 I-MOVE primary care multicentre study. Influenza Other Respir. Viruses 2023; 17:e13069. DOI: 10.1111/irv.13069
140. Klein NP, Fireman B, Goddard K, et al. Vaccine effectiveness of cell-culture relative to egg-based inactivated influenza vaccine during the 2017-18 influenza season. PLoS One 2020; 15:e0229279. DOI: 10.1371/journal.pone.0229279
141. Klick B, Durrani S, Chan KH, et al. Live attenuated seasonal and pandemic influenza vaccine in school-age children: a randomized controlled trial. Vaccine 2013; 31:1937–43. DOI: 10.1016/j.vaccine.2013.02.017
142. Kotecha RS, Wadia UD, Jacoby P, et al. Immunogenicity and clinical effectiveness of the trivalent inactivated influenza vaccine in immunocompromised children undergoing treatment for cancer. Cancer Med. 2016; 5:285–93. DOI: 10.1002/cam4.596
143. Kurecic Filipovic S, Gjenero-Margan I, Kissling E, Kaic B, and Cvitkovic A. Influenza vaccine effectiveness estimates in Croatia in 2010-2011: a season with predominant circulation of A(H1N1)pdm09 influenza virus. Epidemiol. Infect. 2015; 143:2596–603. DOI: 10.1017/S0950268814003677
144. Lei H, Niu B, Sun Z, et al. Influenza vaccine effectiveness against hospital-attended influenza infection in 2023/24 season in Hangzhou, China. medRxiv 2024. DOI: 10.1101/2024.04.29.24306602
145. Lei H, Niu B, Sun Z, et al. Influenza vaccine effectiveness against medically-attended influenza infection in 2023/24 season in Hangzhou, China. Hum. Vaccines Immunother. 2025; 21:2435156. DOI: 10.1080/21645515.2024.2435156
146. Lo YC, Chuang JH, Kuo HW, et al. Surveillance and vaccine effectiveness of an influenza epidemic predominated by vaccine-mismatched influenza B/Yamagata-lineage viruses in Taiwan, 2011-12 season. PLoS One 2013; 8:e58222. DOI: 10.1371/journal.pone.0058222
147. Loeb M, Russell ML, Moss L, et al. Effect of influenza vaccination of children on infection rates in Hutterite communities: a randomized trial. JAMA 2010; 303:943–50. DOI: 10.1001/jama.2010.250
148. Lytras T, Kossyvakis A, Melidou A, et al. Influenza vaccine effectiveness against laboratory confirmed influenza in Greece during the 2013-2014 season: a test-negative study. Vaccine 2015; 33:367–73. DOI: 10.1016/j.vaccine.2014.11.005
149. Lytras T, Kossyvakis A, Melidou A, et al. Influenza vaccine effectiveness in preventing hospitalizations with laboratory-confirmed influenza in Greece during the 2014-2015 season: A test-negative study. J. Med. Virol. 2016; 88:1896–904. DOI: 10.1002/jmv.24551
150. Ma C, Pan Y, Zhang L, et al. Influenza vaccine effectiveness against medically attended influenza illness in Beijing, China, 2014/15 season. Hum. Vaccin. Immunother. 2017; 13:2379–84. DOI: 10.1080/21645515.2017.1359364
151. Ma C, Sun Y, Zhang J, et al. Vaccine effectiveness against influenza B/Victoria-associated medically attended influenza-like illness: Beijing, China, 2021-2022 influenza season. Hum. Vaccines Immunother. 2025; 21:2460859. DOI: 10.1080/21645515.2025.2460859
152. Machado A, Leite A, Larrauri A, Gomez V, Rodrigues AP, Kislaya I, and Nunes B. No effect modification of influenza virus vaccine effectiveness by age or chronic condition was observed in the 2010/11 to 2017/18 seasons. Pharmacoepidemiol. Drug Saf. 2021; 30:1411–9. DOI: 10.1002/pds.5302
153. Martinez-Baz I, Martinez-Artola V, Reina G, et al. Effectiveness of the trivalent influenza vaccine in Navarre, Spain, 2010-2011: a population-based test-negative case-control study. BMC Public Health 2013; 13:191. DOI: 10.1186/1471-2458-13-191

154. Martinez-Baz I, Navascues A, Pozo F, et al. Influenza vaccine effectiveness in preventing inpatient and outpatient cases in a season dominated by vaccine-matched influenza B virus. Hum. Vaccin. Immunother. 2015; 11:1626–33. DOI: 10.1080/21645515.2015.1038002
155. Martinez-Baz I, Casado I, Navascues A, et al. Effect of repeated vaccination with the same vaccine component against 2009 pandemic influenza A(H1N1) virus. J. Infect. Dis 2017; 215:847–55. DOI: 10.1093/infdis/jix055
156. Martinez-Baz I, Navascues A, Casado I, Aguinaga A, Ezpeleta C, and Castilla J. Remaining effect of influenza vaccines received in prior seasons. J. Infect. Dis 2019; 220:1136–40. DOI: 10.1093/infdis/jiz266
157. Martinez-Baz I, Navascues A, Portillo ME, Casado I, Fresan U, Ezpeleta C, and Castilla J. Effect of influenza vaccination in preventing laboratory-confirmed influenza hospitalization in patients with Diabetes Mellitus. Clin. Infect. Dis. 2021; 73:107–14. DOI: 10.1093/cid/ciaa564
158. Martinez-Baz I, Casado I, Miqueleiz A, et al. Effectiveness of influenza vaccination in preventing influenza in primary care, Navarre, Spain, 2021/22. Euro. Surveill. 2022; 27:2200488. DOI: 10.2807/1560-7917.ES.2022.27.26.2200488
159. Martinez-Baz I, Fernandez-Huerta M, Navascues A, et al. Influenza vaccine effectiveness in preventing laboratory-confirmed influenza cases and hospitalizations in Navarre, Spain, 2022-2023. Vaccines 2023; 11:1478. DOI: 10.3390/vaccines11091478
160. Martinez-Baz I, Navascues A, Trobajo-Sanmartin C, et al. Effectiveness of influenza vaccination in preventing confirmed influenza cases and hospitalizations in Northern Spain, 2023/24 season: a population-based test-negative case-control study. Int. J. Infect. Dis. 2025; 151:107364. DOI: 10.1016/j.ijid.2024.107364
161. Maurel M, Pozo F, Perez-Gimeno G, et al. Influenza vaccine effectiveness in Europe: Results from the 2022-2023 VEBIS (Vaccine Effectiveness, Burden and Impact Studies) primary care multicentre study. Influenza Other Respir. Viruses 2024; 18:e13243. DOI: 10.1111/irv.13243
162. Maurel M, Howard J, Kissling E, et al. Interim 2023/24 influenza A vaccine effectiveness: VEBIS European primary care and hospital multicentre studies, September 2023 to January 2024. Euro. Surveill. 2024; 29:2400089. DOI: 10.2807/1560-7917.ES.2024.29.8.2400089
163. McAnerney JM, Walaza S, Cohen AL, et al. Effectiveness and knowledge, attitudes and practices of seasonal influenza vaccine in primary healthcare settings in South Africa, 2010-2013. Influenza Other Respir. Viruses 2015; 9:143–50. DOI: 10.1111/irv.12305
164. McAnerney JM, Treurnicht F, Walaza S, et al. Evaluation of influenza vaccine effectiveness and description of circulating strains in outpatient settings in South Africa, 2014. Influenza Other Respir. Viruses 2015; 9:209–15. DOI: 10.1111/irv.12314
165. McAnerney JM, Walaza S, Tempia S, et al. Estimating vaccine effectiveness in preventing laboratory-confirmed influenza in outpatient settings in South Africa, 2015. Influenza Other Respir. Viruses 2017; 11:177–81. DOI: 10.1111/irv.12436
166. McLean HQ, Caspard H, Griffin MR, et al. Effectiveness of live attenuated influenza vaccine and inactivated influenza vaccine in children during the 2014-2015 season. Vaccine 2017; 35:2685–93. DOI: 10.1016/j.vaccine.2017.03.085
167. McLean HQ, Caspard H, Griffin MR, et al. Association of prior vaccination with influenza vaccine effectiveness in children receiving live attenuated or inactivated vaccine. JAMA Netw. Open 2018; 1:e183742. DOI: 10.1001/jamanetworkopen.2018.3742
168. Menniti-Ippolito F, Da Cas R, Traversa G, et al. Vaccine effectiveness against severe laboratory-confirmed influenza in children: results of two consecutive seasons in Italy. Vaccine 2014; 32:4466–70. DOI: 10.1016/j.vaccine.2014.06.048
169. Mir H, Haq I, and Koul PA. Poor vaccine effectiveness against influenza b-related severe acute respiratory infection in a temperate north indian state (2019-2020): a call for further data for possible vaccines with closer match. Vaccines 2021; 9:1094. DOI: 10.3390/vaccines9101094
170. Mouratidou E, Lambrou A, Andreopoulou A, et al. Influenza vaccine effectiveness against hospitalization with laboratory-confirmed influenza in Greece: A pooled analysis across six seasons, 2013-2014 to 2018-2019. Vaccine 2020; 38:2715–24. DOI: 10.1016/j.vaccine.2020.01.083
171. Mulpuru S, Li L, Ye L, et al. Effectiveness of influenza vaccination on hospitalizations and risk factors for severe outcomes in hospitalized patients With COPD. Chest 2019; 155:69–78. DOI: 10.1016/j.chest.2018.10.044
172. Nation ML, Moss R, Spittal MJ, Kotsimbos T, Kelly PM, and Cheng AC. Influenza vaccine effectiveness against influenza-related mortality in Australian hospitalized patients: a propensity score analysis. Clin. Infect. Dis. 2021; 72:99–107. DOI: 10.1093/cid/ciz1238

173. Neuzil KM, Dupont WD, Wright PF, and Edwards KM. Efficacy of inactivated and cold-adapted vaccines against influenza A infection, 1985 to 1990: the pediatric experience. Pediatr. Infect. Dis. J. 2001; 20:733–40
174. Nicholls S, Carroll K, Crofts J, et al. Outbreak of influenza A (H3N2) in a highly-vaccinated religious community: a retrospective cohort study. Commun. Dis. Public Health 2004; 7:272–7
175. Noble EK, Hayek H, Stewart LS, et al. Effectiveness of influenza vaccination against influenza-associated emergency department (ED) visits and hospitalizations among children with and without underlying medical conditions, New Vaccine Surveillance Network (NVSN), 2015-2016 through 2019-2020 I. Open Forum Infect. Dis. 2023; 10:S805EP–S806. DOI: 10.1093/ofid/ofad500.1564
176. Nolan T, Fortanier AC, Leav B, et al. Efficacy of a cell-culture-derived quadrivalent influenza vaccine in children. N. Engl. J. Med. 2021; 385:1485–95. DOI: 10.1056/NEJMoa2024848
177. Norman DA, Cheng AC, Macartney KK, et al. Influenza hospitalizations in Australian children 2010-2019: The impact of medical comorbidities on outcomes, vaccine coverage, and effectiveness. Influenza Other Respir. Viruses 2022; 16:316–27. DOI: 10.1111/irv.12939
178. Ntshoe GM, McAnerney JM, Tempia S, et al. Influenza epidemiology and vaccine effectiveness among patients with influenza-like illness, viral watch sentinel sites, South Africa, 2005-2009. PLoS One 2014; 9:e94681. DOI: 10.1371/journal.pone.0094681
179. Nunes B, Machado A, Guiomar R, Pechirra P, Conde P, Cristovao P, and Falcao I. Estimates of 2012/13 influenza vaccine effectiveness using the case test-negative control design with different influenza negative control groups. Vaccine 2014; 32:4443–9. DOI: 10.1016/j.vaccine.2014.06.053
180. Ogokeh CE, Campbell AP, Feldstein LR, et al. Comparison of Parental Report of Influenza Vaccination to Documented Records in Children Hospitalized With Acute Respiratory Illness, 2015-2016. Pediatr. Infect. Dis. J. 2021; 10:389–97. DOI: 10.1093/jpids/piaa110
181. Ogra PL, Chow T, Beutner KR, Rubi E, Strussenberg J, DeMello S, and Rizzone C. Clinical and immunologic evaluation of neuraminidase-specific influenza A virus vaccine in humans. J. Infect. Dis 1977; 135:499–506
182. Paradowska-Stankiewicz I, Korczynska MR, Cieslak K, Kowalczyk D, Szymanski K, and Brydak LB. Vaccine effectiveness against influenza in 2015/16 in hospital and ambulatory medical care facilities: Polish results of the European I-MOVE+ multicenter study. Adv. Exp. Med. Biol. 2018; 1023:93–100. DOI: 10.1007/5584{_}2017{_}69
183. Pebody R, Andrews N, Waight P, Malkani R, McCartney C, Ellis J, and Miller E. No effect of 2008/09 seasonal influenza vaccination on the risk of pandemic H1N1 2009 influenza infection in England. Vaccine 2011; 29:2613–8. DOI: 10.1016/j.vaccine.2011.01.046
184. Pebody R, Warburton F, Ellis J, et al. Effectiveness of seasonal influenza vaccine for adults and children in preventing laboratory-confirmed influenza in primary care in the United Kingdom: 2015/16 end-of-season results. Euro. Surveill. 2016; 21. DOI: 10.2807/1560-7917.ES.2016.21.38.30348
185. Pebody R, Warburton F, Ellis J, et al. End-of-season influenza vaccine effectiveness in adults and children, United Kingdom, 2016/17. Euro. Surveill. 2017; 22. DOI: 10.2807/1560-7917.ES.2017.22.44.17-00306
186. Pebody R, Djennad A, Ellis J, et al. End of season influenza vaccine effectiveness in adults and children in the United Kingdom in 2017/18. Euro. Surveill. 2019; 24. DOI: 10.2807/1560-7917.ES.2019.24.31.1800488
187. Pebody RG, Whitaker H, Ellis J, et al. End of season influenza vaccine effectiveness in primary care in adults and children in the United Kingdom in 2018/19. Vaccine 2020; 38:489–97. DOI: 10.1016/j.vaccine.2019.10.071
188. Philip RN, Bell JA, Davis DJ, et al. Epidemiologic studies on influenza in familial and general population groups, 1951-1956. V. Effectiveness of adjuvant vaccines. Am. J. Epidemiol. 1969; 90:471–83
189. Pierse N, Kelly H, Thompson MG, et al. Influenza vaccine effectiveness for hospital and community patients using control groups with and without non-influenza respiratory viruses detected, Auckland, New Zealand 2014. Vaccine 2016; 34:503–9. DOI: 10.1016/j.vaccine.2015.11.073
190. Poehling KA, Caspard H, Peters TR, et al. 2015-2016 Vaccine effectiveness of live attenuated and inactivated influenza vaccines in children in the United States. Clin. Infect. Dis. 2018; 66:665–72. DOI: 10.1093/cid/cix869
191. Puig-Barbera J, Arnedo-Pena A, Pardo-Serrano F, et al. Effectiveness of seasonal 2008-2009, 2009-2010 and pandemic vaccines, to prevent influenza hospitalizations during the autumn 2009 influenza pandemic wave in Castellon, Spain. A test-negative, hospital-based, case-control study. Vaccine 2010; 28:7460–7. DOI: 10.1016/j.vaccine.2010.09.042

192. Puig-Barbera J, Burtseva E, Yu H, Cowling BJ, Badur S, Kyncl J, and Sominina A. Influenza epidemiology and influenza vaccine effectiveness during the 2014-2015 season: annual report from the Global Influenza Hospital Surveillance Network. BMC Public Health 2016; 16 Suppl 1:757. DOI: 10.1186/s12889-016-3378-1
193. Radin JM, Hawksworth AW, Myers CA, Ricketts MN, Hansen EA, and Brice GT. Influenza vaccine effectiveness: maintained protection throughout the duration of influenza seasons 2010-2011 through 2013-2014. Vaccine 2016; 34:3907–12. DOI: 10.1016/j.vaccine.2016.05.034
194. Ray GT, Lewis N, Klein NP, Daley MF, Wang SV, Kulldorff M, and Fireman B. Intraseason waning of influenza vaccine effectiveness. Clin. Infect. Dis. 2019; 68:1623–30. DOI: 10.1093/cid/ciy770
195. Redlberger-Fritz M, Kundi M, and Popow-Kraupp T. Detailed report on 2014/15 influenza virus characteristics, and estimates on influenza virus vaccine effectiveness from Austria's sentinel physician surveillance network. PLoS One 2016; 11:e0149916. DOI: 10.1371/journal.pone.0149916
196. Redlberger-Fritz M, Kundi M, and Popow-Kraupp T. Heterogeneity of circulating influenza viruses and their impact on influenza virus vaccine effectiveness during the influenza seasons 2016/17 to 2018/19 in Austria. Front. Immunol. 2020; 11:434. DOI: 10.3389/fimmu.2020.00434
197. Regan AK, Gibbs R, Bloomfield L, and Effler PV. Estimating influenza vaccine effectiveness using data routinely available in electronic primary care records. Vaccine 2019; 37:755–62. DOI: 10.1016/j.vaccine.2018.12.006
198. Regan AK, Fielding JE, Chilver MB, et al. Intraseason decline in influenza vaccine effectiveness during the 2016 southern hemisphere influenza season: a test-negative design study and phylogenetic assessment. Vaccine 2019; 37:2634–41. DOI: 10.1016/j.vaccine.2019.02.027
199. Rizzo C, Bella A, Alfonsi V, et al. Influenza vaccine effectiveness in Italy: age, subtype-specific and vaccine type estimates 2014/15 season. Vaccine 2016; 34:3102–8. DOI: 10.1016/j.vaccine.2016.04.072
200. Rizzo C, Gesualdo F, Loconsole D, et al. Moderate vaccine effectiveness against severe acute respiratory infection caused by a(H1N1)pdm09 influenza virus and no effectiveness against a(H3N2)) influenza virus in the 2018/2019 season in Italy. Vaccines 2020; 8:1–10. DOI: 10.3390/vaccines8030427
201. Rose AM, Lucaccioni H, Marsh K, et al. Interim 2024/25 influenza vaccine effectiveness: eight European studies, September 2024 to January 2025. Euro. Surveill. 2025; 30. DOI: 10.2807/1560-7917.ES.2025.30.7.2500102
202. Saito N, Komori K, Suzuki M, Morimoto K, Kishikawa T, Yasaka T, and Ariyoshi K. Negative impact of prior influenza vaccination on current influenza vaccination among people infected and not infected in prior season: A test-negative case-control study in Japan. Vaccine 2017; 35:687–93. DOI: 10.1016/j.vaccine.2016.11.024
203. Salleras L, Dominguez A, Pumarola T, et al. Effectiveness of virosomal subunit influenza vaccine in preventing influenza-related illnesses and its social and economic consequences in children aged 3-14 years: a prospective cohort study. Vaccine 2006; 24:6638–42
204. Sanada Y, Yakushijin K, Nomura T, et al. A prospective study on the efficacy of two-dose influenza vaccinations in cancer patients receiving chemotherapy. Jpn J. Clin. Oncol. 2016; 46:448–52. DOI: 10.1093/jjco/hyw020
205. Savulescu C, Jimenez-Jorge S, Mateo S de, Ledesma J, Pozo F, Casas I, and Larrauri A. Effectiveness of the 2010/11 seasonal trivalent influenza vaccine in Spain: preliminary results of a case-control study. Euro. Surveill. 2011; 16
206. Savulescu C, Jimenez-Jorge S, Mateo S de, et al. Using surveillance data to estimate pandemic vaccine effectiveness against laboratory confirmed influenza A(H1N1)2009 infection: two case-control studies, Spain, season 2009-2010. BMC Public Health 2011; 11:899. DOI: 10.1186/1471-2458-11-899
207. Savulescu C, Jimenez-Jorge S, Delgado-Sanz C, Mateo S de, Pozo F, Casas I, and Larrauri A. Higher vaccine effectiveness in seasons with predominant circulation of seasonal influenza A(H1N1) than in A(H3N2) seasons: test-negative case-control studies using surveillance data, Spain, 2003-2011. Vaccine 2014; 32:4404–11. DOI: 10.1016/j.vaccine.2014.06.063
208. Schuurmans MM, Tini GM, Dalar L, Fretz G, Benden C, and Boehler A. Pandemic 2009 H1N1 influenza virus vaccination in lung transplant recipients: coverage, safety and clinical effectiveness in the Zurich cohort. J. Heart Lung Transplant 2011; 30:685–90. DOI: 10.1016/j.healun.2011.01.707
209. Separovic L, Zhan Y, Kaweski SE, et al. Interim estimates of vaccine effectiveness against influenza A(H1N1)pdm09 and A(H3N2) during a delayed influenza season, Canada, 2024/25. Euro. Surveill. 2025; 30:pii=2500059. DOI: 10.2807/1560-7917.ES.2025.30.4.2500059

210. Shang M, Chung JR, Jackson ML, et al. Influenza vaccine effectiveness among patients with high-risk medical conditions in the United States, 2012-2016. Vaccine 2018; 36:8047–53. DOI: 10.1016/j.vaccine.2018.10.093
211. Shinjoh M, Sugaya N, Yamaguchi Y, et al. Effectiveness of trivalent inactivated influenza vaccine in children estimated by a test-negative case-control design study based on influenza rapid diagnostic test results. PLoS One 2015; 10:e0136539. DOI: 10.1371/journal.pone.0136539
212. Shinjoh M, Sugaya N, Yamaguchi Y, et al. Inactivated influenza vaccine effectiveness and an analysis of repeated vaccination for children during the 2016/17 season. Vaccine 2018; 36:5510–8. DOI: 10.1016/j.vaccine.2018.07.065
213. Simpson CR, Ritchie LD, Robertson C, Sheikh A, and McMenamin J. Vaccine effectiveness in pandemic influenza - primary care reporting (VIPER): an observational study to assess the effectiveness of the pandemic influenza A (H1N1)v vaccine. Health technology assessment (Winchester, England) 2010; 14:313–46. DOI: 10.3310/hta14340-05
214. Simpson CR, Lone NI, Kavanagh K, Ritchie LD, Robertson C, Sheikh A, and McMenamin J. Trivalent inactivated seasonal influenza vaccine effectiveness for the prevention of laboratory-confirmed influenza in a Scottish population 2000 to 2009. Euro. Surveill. 2015; 20
215. Anonymous. Effectiveness of vaccine against medical consultation due to laboratory-confirmed influenza: results from a sentinel physician pilot project in British Columbia, 2004-2005. Can. Commun. Dis. Rep. 2005; 31:181–91
216. Skowronski DM, Masaro C, Kwindt TL, et al. Estimating vaccine effectiveness against laboratory-confirmed influenza using a sentinel physician network: results from the 2005-2006 season of dual A and B vaccine mismatch in Canada. Vaccine 2007; 25:2842–51
217. Skowronski DM, Janjua NZ, De Serres G, et al. A sentinel platform to evaluate influenza vaccine effectiveness and new variant circulation, Canada 2010-2011 season. Clin. Infect. Dis. 2012; 55:332–42. DOI: 10.1093/cid/cis431
218. Skowronski DM, Janjua NZ, Sabaiduc S, et al. Influenza A/subtype and B/lineage effectiveness estimates for the 2011-2012 trivalent vaccine: cross-season and cross-lineage protection with unchanged vaccine. J. Infect. Dis 2014; 210:126–37
219. Skowronski DM, Janjua NZ, De Serres G, et al. Low 2012-13 influenza vaccine effectiveness associated with mutation in the egg-adapted H3N2 vaccine strain not antigenic drift in circulating viruses. PLoS One 2014; 9:e92153. DOI: 10.1371/journal.pone.0092153
220. Skowronski DM, Chambers C, Sabaiduc S, et al. Integrated sentinel surveillance linking genetic, antigenic, and epidemiologic monitoring of influenza vaccine-virus relatedness and effectiveness during the 2013-2014 influenza season. J. Infect. Dis 2015; 212:726–39. DOI: 10.1093/infdis/jiv177
221. Skowronski DM, Chambers C, Sabaiduc S, et al. A perfect storm: impact of genomic variation and serial vaccination on low influenza vaccine effectiveness during the 2014-2015 season. Clin. Infect. Dis. 2016; 63:21–32. DOI: 10.1093/cid/ciw176
222. Skowronski DM, Chambers C, Sabaiduc S, et al. Beyond antigenic match: possible agent-host and immuno-epidemiological influences on influenza vaccine effectiveness during the 2015-2016 season in Canada. J. Infect. Dis 2017; 216:1487–500. DOI: 10.1093/infdis/jix526
223. Skowronski DM, Sabaiduc S, Leir S, et al. Paradoxical clade- and age-specific vaccine effectiveness during the 2018/19 influenza A(H3N2) epidemic in Canada: potential imprint-regulated effect of vaccine (I-REV). Euro. Surveill. 2019; 24. DOI: 10.2807/1560-7917.ES.2019.24.46.1900585
224. Skowronski DM, Chambers C, De Serres G, et al. Vaccine effectiveness against lineage-matched and -mismatched influenza B Viruses across 8 seasons in Canada, 2010-2011 to 2017-2018. Clin. Infect. Dis. 2019; 68:1754–7. DOI: 10.1093/cid/ciy876
225. Skowronski DM, Chuang ESY, Sabaiduc S, et al. Vaccine effectiveness estimates from an early-season influenza A(H3N2) epidemic, including unique genetic diversity with reassortment, Canada, 2022/23. Euro. Surveill. 2023; 28. DOI: 10.2807/1560-7917.ES.2023.28.5.2300043
226. Slepushkin AN, Obrosova-Serova NP, Burtseva EI, et al. Comparison of live attenuated and inactivated influenza vaccines in schoolchildren in Russia: I. Safety and efficacy in two Moscow schools, 1987/88. Vaccine 1993; 11:323–8
227. Slobodniuk AV, Romanenko VV, Utnitskaia OS, Motus TM, and Pereverzev AV. [Influence of multiplicity of immunizations of children with inactivated influenza vaccine on immune response and the effectiveness of protection]. Zh. Mikrobiol. Epidemiol. Immunobiol. 2002 :36–9
228. Smith ER, Fry AM, Hicks LA, et al. Reducing antibiotic use in ambulatory care through influenza vaccination. Clin. Infect. Dis. 2020; 71:E726–E734. DOI: 10.1093/cid/ciaa464

229. Smithgall M, Vargas CY, Reed C, et al. Influenza vaccine effectiveness in a low-income, urban community cohort. Clin. Infect. Dis. 2016; 62:358–60. DOI: 10.1093/cid/civ867
230. Sohn YJ, Choi JH, Choi YY, et al. Effectiveness of trivalent inactivated influenza vaccines in children during 2017-2018 season in Korea: comparison of test-negative analysis by rapid and RT-PCR influenza tests. Int. J. Infect. Dis. 2020; 99:199–203. DOI: 10.1016/j.ijid.2020.07.032
231. Soldevila N, Basile L, Martinez A, et al. Surveillance of influenza B severe hospitalized cases during 10 seasons in Catalonia: Does the lineage make a difference?. J. Med. Virol. 2022; 94:4417–24. DOI: 10.1002/jmv.27876
232. Steens A, Wijnans EG, Dieleman JP, Sturkenboom MC, Sande MA van der, and Hoek W van der. Effectiveness of a MF-59 TM-adjuvanted pandemic influenza vaccine to prevent 2009 A/H1N1 influenza-related hospitalisation; a matched case-control study. BMC Infect. Dis. 2011; 11:196. DOI: 10.1186/1471-2334-11-196
233. Stuurman AL, Bollaerts K, Alexandridou M, et al. Vaccine effectiveness against laboratory-confirmed influenza in Europe - results from the DRIVE network during season 2018/19. Vaccine 2020; 38:6455–63. DOI: 10.1016/j.vaccine.2020.07.063
234. Su Y, Guo Z, Gu X, Sun S, Wang K, Xie S, and Zhao S. Influenza vaccine effectiveness against influenza A during the delayed 2022/23 epidemic in Shihezi, China. Vaccine 2023; 41:5683EP–5686. DOI: 10.1016/j.vaccine.2023.08.039
235. Sullivan SG, Komadina N, Grant K, Jelley L, Papadakis G, and Kelly H. Influenza vaccine effectiveness during the 2012 influenza season in Victoria, Australia: influences of waning immunity and vaccine match. J. Med. Virol. 2014; 86:1017–25. DOI: 10.1002/jmv.23847
236. Sullivan SG, Chilver MBN, Higgins G, Cheng AC, and Stocks NP. Influenza vaccine effectiveness in Australia: results from the Australian Sentinel Practices Research Network. Med. J. Aust. 2014; 201:109–11
237. Sullivan SG, Carville KS, Chilver M, et al. Pooled influenza vaccine effectiveness estimates for Australia, 2012-2014. Epidemiol. Infect. 2016; 144:2317–28. DOI: 10.1017/S0950268816000819
238. Suzuki M, Minh LN, Yoshimine H, Inoue K, Yoshida LM, Morimoto K, and Ariyoshi K. Vaccine effectiveness against medically attended laboratory-confirmed influenza in Japan, 2011-2012 Season. PLoS One 2014; 9:e88813. DOI: 10.1371/journal.pone.0088813
239. Sykes A, Gerhardt E, Tang L, and Adderson EE. The effectiveness of trivalent inactivated influenza vaccine in children with acute leukemia. J. Pediatr. 2017; 191:218–24. DOI: 10.1016/j.jpeds.2017.08.071
240. Tam YH, Ng TWY, Chu DKW, Fang VJ, Cowling BJ, Malik Peiris JS, and Ip DKM. The effectiveness of influenza vaccination against medically-attended illnesses in Hong Kong across three years with different degrees of vaccine match, 2014-17. Vaccine 2018; 36:6117–23. DOI: 10.1016/j.vaccine.2018.08.075
241. Thomas HL, Andrews N, Green HK, et al. Estimating vaccine effectiveness against severe influenza in England and Scotland 2011/2012: applying the screening method to data from intensive care surveillance systems. Epidemiol. Infect. 2014; 142:126–33. DOI: 10.1017/S0950268813000824
242. Thompson MG, Sokolow LZ, Almendares O, et al. Effectiveness of nonadjuvanted monovalent influenza A(H1N1)pdm09 vaccines for preventing reverse transcription polymerase chain reaction-confirmed pandemic influenza hospitalizations: case-control study of children and adults at 10 US influenza surveillance Network Sites. Clin. Infect. Dis. 2013; 57:1587–92. DOI: 10.1093/cid/cit551
243. Thors V, Vias RD, Bjornsdottir K, Palsdottir EB, Gufinnsdottir GK, and Haraldsson A. Influenza vaccine effectiveness in Iceland 2014-2022: a test-negative design. Vaccine 2025; 55:126981. DOI: 10.1016/j.vaccine.2025.126981
244. Torner N, Martinez A, Basile L, et al. Influenza vaccine effectiveness assessment through sentinel virological data in three post-pandemic seasons. Hum. Vaccin. Immunother. 2015; 11:225–30. DOI: 10.4161/hv.36155
245. Turner N, Pierse N, Bissielo A, et al. Effectiveness of seasonal trivalent inactivated influenza vaccine in preventing influenza hospitalisations and primary care visits in Auckland, New Zealand, in 2013. Euro. Surveill. 2014; 19
246. Uphoff H, Heiden M An der, Schweiger B, et al. Effectiveness of the AS03-adjuvanted vaccine against pandemic influenza virus A/(H1N1) 2009–a comparison of two methods; Germany, 2009/10. PLoS One 2011; 6:e19932. DOI: 10.1371/journal.pone.0019932

247. Uzicanin A, Thompson M, Smith P, et al. Effectiveness of 1 dose of influenza A (H1N1) 2009 monovalent vaccines in preventing reverse-transcription polymerase chain reaction-confirmed H1N1 infection among school-aged children in maine. J. Infect. Dis 2012; 206:1059–68
248. Vaikutyte R, Kuliese M, Mickiene A, Jancoriene L, Zablockiene B, and Gefenaite G. Influenza vaccine effectiveness in patients hospitalized with severe acute respiratory infection in Lithuania during the 2019-2020 influenza season: a test negative case - control study. Virol. J. 2023; 20:67. DOI: 10.1186/s12985-023-02015-0
249. Valenciano M, Kissling E, Cohen JM, et al. Estimates of pandemic influenza vaccine effectiveness in Europe, 2009-2010: results of Influenza Monitoring Vaccine Effectiveness in Europe (I-MOVE) multicentre case-control study. PLoS Med. 2011; 8:e1000388. DOI: 10.1371/journal.pmed.1000388
250. Valenciano M, Kissling E, Reuss A, et al. The European I-MOVE multicentre 2013-2014 case-control study. Homogeneous moderate influenza vaccine effectiveness against A(H1N1)pdm09 and heterogenous results by country against A(H3N2). Vaccine 2015; 33:2813–22. DOI: 10.1016/j.vaccine.2015.04.012
251. Valenciano M, Kissling E, Reuss A, et al. Vaccine effectiveness in preventing laboratory-confirmed influenza in primary care patients in a season of co-circulation of influenza A(H1N1)pdm09, B and drifted A(H3N2), I-MOVE Multicentre case-control study, Europe 2014/15. Euro. Surveill. 2016; 21:pii. DOI: 10.2807/1560-7917.ES.2016.21.7.30139
252. Valenciano M, Kissling E, Larrauri A, et al. Exploring the effect of previous inactivated influenza vaccination on seasonal influenza vaccine effectiveness against medically attended influenza: results of the European I-MOVE multicentre test-negative case-control study, 2011/2012-2016/2017. Influenza Other Respir. Viruses 2018; 12:567–81. DOI: 10.1111/irv.12562
253. Vasileiou E, Sheikh A, Butler CC, et al. Seasonal influenza vaccine effectiveness in people with asthma: a national test-negative design case-control study. Clin. Infect. Dis. 2020; 71:e94–e104. DOI: 10.1093/cid/ciz1086
254. Vasilyeva RI, Lyamtseva GA, and Ryazantseva TG. Evaluation of the protective properties and safety of inactivated influenza vaccine and the effectiveness of the immunization of school children in spring. Zh. Mikrobiol. Epidemiol. Immunobiol. 1986; 63:10–14
255. Vilcu AM, Souty C, Enouf V, et al. Estimation of seasonal influenza vaccine effectiveness using data collected in primary care in France: comparison of the test-negative design and the screening method. Clin. Microbiol. Infect. 2018; 24:5–431. DOI: 10.1016/j.cmi.2017.09.003
256. Wang B, Russell ML, Moss L, et al. Effect of influenza vaccination of children on infection rate in Hutterite communities: follow-up study of a randomized trial. PLoS One 2016; 11:e0167281. DOI: 10.1371/journal.pone.0167281
257. Wang S, Zheng Y, Jin X, et al. Efficacy and safety of a live attenuated influenza vaccine in Chinese healthy children aged 3-17 years in one study center of a randomized, double-blind, placebo-controlled phase 3 clinical trial, 2016/17 season. Vaccine 2020; 38:5979–86. DOI: 10.1016/j.vaccine.2020.07.019
258. Whitaker H, Willam N, Cottrell S, et al. End of 2022/23 season influenza vaccine effectiveness in primary care in Great Britain. Influenza Other Respir. Viruses 2024; 18:e13295. DOI: 10.1111/irv.13295
259. Whitaker HJ, Hassell K, Hoschler K, et al. Influenza vaccination during the 2021/22 season: a data-linkage test-negative case-control study of effectiveness against influenza requiring emergency care in England and serological analysis of primary care patients. Vaccine 2024; 42:1656EP–1664. DOI: 10.1016/j.vaccine.2024.02.006
260. Wichmann O, Stocker P, Poggensee G, et al. Pandemic influenza A(H1N1) 2009 breakthrough infections and estimates of vaccine effectiveness in Germany 2009-2010. Euro. Surveill. 2010; 15
261. Yang P, Thompson MG, Ma C, Shi W, Wu S, Zhang D, and Wang Q. Influenza vaccine effectiveness against medically-attended influenza illness during the 2012-2013 season in Beijing, China. Vaccine 2014; 32:5285–9. DOI: 10.1016/j.vaccine.2014.07.083
262. Yaron-Yakoby H, Sefty H, Pando R, et al. Effectiveness of influenza vaccine in preventing medically-attended influenza virus infection in primary care, Israel, influenza seasons 2014/15 and 2015/16. Euro. Surveill. 2018; 23. DOI: 10.2807/1560-7917.ES.2018.23.7.17-00026
263. Yoon Y, Choi JS, Park M, et al. Influenza vaccine effectiveness in children at the emergency department during the 2018-2019 season: the first season school-aged children were included in the Korean Influenza National Immunization Program. J. Korean Med. Sci. 2021; 36:e71. DOI: 10.3346/jkms.2021.36.e71

264. Zaplatnikov AL. [Specific prophylaxis of influenza in organized groups of children with the vaccine Vaxigrip]. Zh. Mikrobiol. Epidemiol. Immunobiol. 2001 :36–40
265. Zhang L, Yang P, Thompson MG, et al. Influenza vaccine effectiveness in preventing influenza illness among children during school-based outbreaks in the 2014-2015 season in Beijing, China. Pediatr. Infect. Dis. J. 2017; 36:e69–e75. DOI: 10.1097/INF.0000000000001434
266. Zhang L, Pan Y, Hackert V, et al. The 2015-2016 influenza epidemic in Beijing, China: unlike elsewhere, circulation of influenza A(H3N2) with moderate vaccine effectiveness. Vaccine 2018; 36:4993–5001. DOI: 10.1016/j.vaccine.2018.07.017
267. Zhang L, Hoek W van der, Krafft T, et al. Influenza vaccine effectiveness estimates against influenza A(H3N2) and A(H1N1) pdm09 among children during school-based outbreaks in the 2016-2017 season in Beijing, China. Hum. Vaccin. Immunother. 2020; 16:816–22. DOI: 10.1080/21645515.2019.1677438
268. Zhang L, Lu G, Ma C, et al. influenza vaccine effectiveness against influenza A-associated outpatient and emergency-department-attended influenza-like illness during the delayed 2022-2023 season in Beijing, China. Vaccines 2024; 12:1124. DOI: 10.3390/vaccines12101124
269. Zhu S, Quint J, Leon TM, et al. Interim influenza vaccine effectiveness against ;aboratory-confirmed influenza - California, October 2023-January 2024. MMWR Morb. Mortal Wkly. Rep. 2024; 73:175EP–179. DOI: 10.15585/mmwr.mm7308a4
270. Lusignan S de, Hoang U, Liyanage H, et al. Using point of care testing to estimate influenza vaccine effectiveness in the English primary care sentinel surveillance network. PLoS One 2021; 16:e0248123. DOI: 10.1371/journal.pone.0248123
271. Doorn E van, Darvishian M, Dijkstra F, Donker GA, Overduin P, Meijer A, and Hak E. Influenza vaccine effectiveness estimates in the Dutch population from 2003 to 2014: The test-negative design case-control study with different control groups. Vaccine 2017; 35:2831–9. DOI: 10.1016/j.vaccine.2017.04.012
272. Ortqvist A, Berggren I, Insulander M, Jong B de, and Svenungsson B. Effectiveness of an adjuvanted monovalent vaccine against the 2009 pandemic strain of influenza A(H1N1)v, in Stockholm County, Sweden. Clin. Infect. Dis. 2011; 52:1203–11. DOI: 10.1093/cid/cir182
273. Ortqvist A, Bennet R, Rinder MR, Lindblad H, and Eriksson M. Effectiveness of the monovalent AS03-adjuvanted influenza A(H1N1)pdm09 vaccine against hospitalization in children because of influenza. Vaccine 2012; 30:5699–702. DOI: 10.1016/j.vaccine.2012.07.009
274. Adams K, Weber ZA, D.-H. Y, et al. Vaccine effectiveness against pediatric influenza-A-associated urgent care, emergency department, and hospital encounters during the 2022-2023 season: VISION Network. Clin. Infect. Dis. 2024; 78:746EP–755. DOI: 10.1093/cid/ciad704
275. Anderson KB, Simasathien S, Watanaveeradej V, et al. Clinical and laboratory predictors of influenza infection among individuals with influenza-like illness presenting to an urban Thai hospital over a five-year period. PLoS One 2018; 13:e0193050. DOI: 10.1371/journal.pone.0193050
276. Ando S. Effectiveness of quadrivalent influenza vaccine based on the test-negative control study in children during the 2016-2017 season. J. Infect. Chemother. 2018; 24:782–8. DOI: 10.1016/j.jiac.2018.05.012
277. Ando S. Effectiveness of current and repeated vaccination with quadrivalent influenza vaccine during the 2017/18 season in Japan. J. Antibiot. (Tokyo) 2019; 72:143–55
278. Ando S. Estimation of the effectiveness of quadrivalent influenza vaccines by distinguishing between influenza A(H1N1)pdm09 and influenza A(H3N2) Using rapid influenza diagnostic tests during the 2018-2019 season. Intern. Med. 2020; 59:933–40. DOI: 10.2169/internalmedicine.3616-19
279. Ando S. Effectiveness of the 2019-2020 influenza vaccine and the effect of prior influenza infection and vaccination in children during the first influenza season overlapping with the COVID-19 epidemic. J. Nippon Med. Sch. 2021; 88:524–32. DOI: 10.1272/jnms.JNMS.2022{_}89-102
280. Andrews N, McMenamin J, Durnall H, et al. Effectiveness of trivalent seasonal influenza vaccine in preventing laboratory-confirmed influenza in primary care in the United Kingdom: 2012/13 end of season results. Euro. Surveill. 2014; 19:5–13
281. Belongia EA, Kieke BA, Donahue JG, et al. Influenza vaccine effectiveness in Wisconsin during the 2007-08 season: comparison of interim and final results. Vaccine 2011; 29:6558–63. DOI: 10.1016/j.vaccine.2011.07.002
282. Bi Q, Dickerman BA, Nguyen HQ, et al. Reduced effectiveness of repeat influenza vaccination: distinguishing among within-season waning, recent clinical infection, and subclinical infection. J. Infect. Dis 2024; 230:1309–18. DOI: 10.1093/infdis/jiae220

283. Blyth CC, Jacoby P, Effler PV, et al. Influenza vaccine effectiveness and uptake in children at risk of severe disease. Pediatr. Infect. Dis. J. 2016; 35:309–15. DOI: 10.1097/INF.0000000000000999
284. Blyth CC, Macartney KK, Hewagama S, et al. Influenza epidemiology, vaccine coverage and vaccine effectiveness in children admitted to sentinel Australian hospitals in 2014: the Influenza Complications Alert Network (FluCAN). Euro. Surveill. 2016; 21. DOI: 10.2807/1560-7917.ES.2016.21.30.30301
285. Blyth CC, Macartney KK, McRae J, et al. Influenza epidemiology, vaccine coverage and vaccine effectiveness in children admitted to sentinel Australian hospitals in 2017: results from the PAEDS-FluCAN collaboration. Clin. Infect. Dis. 2019; 68:940–8. DOI: 10.1093/cid/ciy597
286. Blyth CC, Cheng AC, Crawford NW, et al. The impact of new universal child influenza programs in Australia: Vaccine coverage, effectiveness and disease epidemiology in hospitalised children in 2018. Vaccine 2020; 38:2779–87. DOI: 10.1016/j.vaccine.2020.02.031
287. Boddington NL, Mangtani P, Zhao H, Verlander NQ, Ellis J, Andrews N, and Pebody RG. Live-attenuated influenza vaccine effectiveness against hospitalization in children aged 2-6 years, the first three seasons of the childhood influenza vaccination program in England, 2013/14-2015/16. Influenza Other Respir. Viruses 2022; 16:897–905. DOI: 10.1111/irv.12990
288. Buchan SA, Booth S, Scott AN, et al. Effectiveness of live attenuated vs inactivated influenza vaccines in children during the 2012-2013 through 2015-2016 influenza seasons in Alberta, Canada: a Canadian Immunization Research Network (CIRN) Study. JAMA Pediatr. 2018; 172:e181514. DOI: 10.1001/jamapediatrics.2018.1514
289. Campbell AP, Ogokeh CE, Weinberg GA, et al. Vaccine effectiveness against influenza-associated hospitalizations and emergency department (ED) visits among children in The United States in the 2019-2020 season. Open Forum Infect. Dis. 2020; 7:S217–S218. DOI: 10.1093/ofid/ofaa417.488
290. Campbell AP, Ogokeh C, Lively JY, et al. Vaccine effectiveness against pediatric influenza hospitalizations and emergency visits. Pediatrics 2020; 146. DOI: 10.1542/peds.2020-1368
291. Campbell AP, Ogokeh C, Weinberg GA, et al. Effect of vaccination on preventing influenza-associated hospitalizations among children during a severe season associated with B/Victoria viruses, 2019-2020. Clin. Infect. Dis. 2021; 73:e947–e954. DOI: 10.1093/cid/ciab060
292. Caspard H, Gaglani M, Clipper L, et al. Effectiveness of live attenuated influenza vaccine and inactivated influenza vaccine in children 2-17 years of age in 2013-2014 in the United States. Vaccine 2016; 34:77–82. DOI: 10.1016/j.vaccine.2015.11.010
293. Castillejos M, Cabello-Gutierrez C, Alberto Choreno-Parra J, et al. High performance of rapid influenza diagnostic test and variable effectiveness of influenza vaccines in Mexico. Int. J. Infect. Dis. 2019; 89:87–95. DOI: 10.1016/j.ijid.2019.08.029
294. Chard AN, Nogareda F, Regan AK, et al. End-of-season influenza vaccine effectiveness during the southern hemisphere 2022 influenza season - Chile, Paraguay, and Uruguay. Int. J. Infect. Dis. 2023; 134:39–44. DOI: 10.1016/j.ijid.2023.05.015
295. Chiu SS, Feng S, Chan KH, et al. Hospital-based vaccine effectiveness against influenza B lineages, Hong Kong, 2009-14. Vaccine 2016; 34:2164–9. DOI: 10.1016/j.vaccine.2016.03.032
296. Chiu SS, Kwan MYW, Feng S, et al. Influenza vaccine effectiveness against influenza A(H3N2) hospitalizations in children in Hong Kong in a prolonged season, 2016/2017. J. Infect. Dis 2018; 217:1365–71. DOI: 10.1093/infdis/jiy027
297. Chiu SS, Chua H, Kwan MYW, Chan ELY, Wong JSC, Peiris JSM, and Cowling BJ. Influenza vaccination effectiveness in preventing influenza hospitalization in children, Hong Kong, winter 2019/20. Vaccine 2020; 38:8078–81. DOI: 10.1016/j.vaccine.2020.10.081
298. Chon I, Saito R, Hibino A, et al. Effectiveness of the quadrivalent inactivated influenza vaccine in Japan during the 2015-2016 season: A test-negative case-control study comparing the results by real time PCR, virus isolation. Vaccine: X 2019; 1:100011. DOI: 10.1016/j.jvacx.2019.100011
299. Chua H, Kwan MYW, Chan ELY, Wong JSC, Peiris JSM, Cowling BJ, and Chiu SS. Influenza vaccine effectiveness against influenza-associated hospitalization in children in Hong Kong, 2010-2020. Vaccine 2021; 39:4842–8. DOI: 10.1016/j.vaccine.2021.07.014
300. Clover RD, Crawford S, Glezen WP, Taber LH, Matson CC, and Couch RB. Comparison of heterotypic protection against influenza A/Taiwan/86 (H1N1) by attenuated and inactivated vaccines to A/Chile/83-like viruses. J. Infect. Dis 1991; 163:300–4
301. Colucci ME, Affanni P, Cantarelli A, et al. Influenza vaccine effectiveness in children: a retrospective study on eight post-pandemic seasons with trivalent inactivated vaccine. Acta Biomed. 2020; 91:63–70. DOI: 10.23750/abm.v91i3-S.9424

302. Cowling BJ, Feng S, Finelli L, Steffens A, and Fowlkes A. Assessment of influenza vaccine effectiveness in a sentinel surveillance network 2010-13, United States. Vaccine 2016; 34:61–6. DOI: 10.1016/j.vaccine.2015.11.016
303. Cowling BJ, Kwan MYW, Murphy C, et al. Influenza vaccine effectiveness against influenza-associated hospitalization in Hong Kong children aged 9 months to 17 years, March-June 2023. Pediatr. Infect. Dis. J. 2023; 12:586EP–589. DOI: 10.1093/jpids/piad083
304. Diallo A, Diop OM, Diop D, et al. Effectiveness of seasonal influenza vaccination in children in Senegal during a year of vaccine mismatch: a cluster-randomized trial. Clin. Infect. Dis. 2019; 69:1780–8. DOI: 10.1093/cid/ciz066
305. Dixon GA, Moore HC, Kelly H, et al. Lessons from the first year of the WAIVE study investigating the protective effect of influenza vaccine against laboratory-confirmed influenza in hospitalised children aged 6-59 months. Influenza Other Respir. Viruses 2010; 4:231–4. DOI: 10.1111/j.1750-2659.2010.00141.x
306. Drori Y, Pando R, Sefty H, et al. Influenza vaccine effectiveness against laboratory-confirmed influenza in a vaccine-mismatched influenza B-dominant season. Vaccine 2020; 38:8387–95. DOI: 10.1016/j.vaccine.2020.10.074
307. El Omeiri N, Azziz-Baumgartner E, Clara W, et al. Pilot to evaluate the feasibility of measuring seasonal influenza vaccine effectiveness using surveillance platforms in Central-America, 2012. BMC Public Health 2015; 15:673. DOI: 10.1186/s12889-015-2001-1
308. El Omeiri N, Azziz-Baumgartner E, Thompson MG, et al. Seasonal influenza vaccine effectiveness against laboratory-confirmed influenza hospitalizations - Latin America, 2013. Vaccine 2018; 36:3555–66. DOI: 10.1016/j.vaccine.2017.06.036
309. Feldstein LR, Ogokeh C, Rha B, et al. Vaccine effectiveness against influenza hospitalization among children in the United States, 2015-2016. Pediatr. Infect. Dis. J. 2021; 10:75–82. DOI: 10.1093/jpids/piaa017
310. Feng S, Chiu SS, Chan ELY, et al. Effectiveness of influenza vaccination on influenza-associated hospitalisations over time among children in Hong Kong: a test-negative case-control study. Lancet Respir. Med. 2018; 6:925–34. DOI: 10.1016/S2213-2600(18)30419-3
311. Flannery B, Zimmerman RK, Gubareva LV, et al. Enhanced genetic characterization of influenza A(H3N2) viruses and vaccine effectiveness by genetic group, 2014-2015. J. Infect. Dis 2016; 214:1010–9. DOI: 10.1093/infdis/jiw181
312. Flannery B, Reynolds SB, Blanton L, et al. Influenza vaccine effectiveness against pediatric deaths: 2010-2014. Pediatrics 2017; 139. DOI: 10.1542/peds.2016-4244
313. Flannery B, Chung JR, Monto AS, et al. Influenza vaccine effectiveness in the United States during the 2016-2017 season. Clin. Infect. Dis. 2019; 68:1798–806. DOI: 10.1093/cid/ciy775
314. Flannery B, Kondor RJG, Chung JR, et al. Spread of antigenically drifted influenza A(H3N2) viruses and vaccine effectiveness in the United States during the 2018-2019 season. J. Infect. Dis 2020; 221:8–15. DOI: 10.1093/infdis/jiz543
315. Fu C, He Q, Li Z, et al. Seasonal influenza vaccine effectiveness among children, 2010-2012. Influenza Other Respir. Viruses 2013; 7:1168–74. DOI: 10.1111/irv.12157
316. Fu C, Xu J, He Q, Li Z, and Liu F. Seasonal influenza vaccine effectiveness in children during 2010-2011 season: a case-cohort study. Hum. Vaccin. Immunother. 2013; 9:987–8. DOI: 10.4161/hv.23457
317. Fu C, Xu J, Lin J, Wang M, Li K, Ge J, and Thompson MG. Concurrent and cross-season protection of inactivated influenza vaccine against A(H1N1)pdm09 illness among young children: 2012-2013 case-control evaluation of influenza vaccine effectiveness. Vaccine 2015; 33:2917–21. DOI: 10.1016/j.vaccine.2015.04.063
318. Fu C, Greene CM, He Q, et al. Dose effect of influenza vaccine on protection against laboratory-confirmed influenza illness among children aged 6 months to 8 years of age in southern China, 2013/14-2015/16 seasons: a matched case-control study. Hum. Vaccin. Immunother. 2020; 16:595–601. DOI: 10.1080/21645515.2019.1662267
319. Gao X, Sun Y, Shen P, et al. Population-based influenza vaccine effectiveness against laboratory-confirmed influenza infection in southern China, 2023-2024 season. Open Forum Infect. Dis. 2024; 11:ofae456. DOI: 10.1093/ofid/ofae456
320. Glatman-Freedman A, Pando R, Sefty H, et al. Predominance of a drifted influenza A (H3N2) clade and its association with age-specific influenza vaccine effectiveness variations, influenza season 2018-2019. Vaccines 2020; 8. DOI: 10.3390/vaccines8010078

321. Grijalva CG, Zhu Y, Williams DJ, et al. Association between hospitalization with community-acquired laboratory-confirmed influenza pneumonia and prior receipt of influenza accination. JAMA 2015; 314:1488–97. DOI: 10.1001/jama.2015.12160
322. Halloran ME, Longini IMJ, Gaglani MJ, Piedra PA, Chu H, Herschler GB, and Glezen WP. Estimating efficacy of trivalent, cold-adapted, influenza virus vaccine (CAIV-T) against influenza A (H1N1) and B using surveillance cultures. Am. J. Epidemiol. 2003; 158:305–11
323. Halloran ME, Piedra PA, Longini IMJ, et al. Efficacy of trivalent, cold-adapted, influenza virus vaccine against influenza A (Fujian), a drift variant, during 2003-2004. Vaccine 2007; 25:4038–45
324. Hardelid P, Fleming DM, Andrews N, Barley M, Durnall H, Mangtani P, and Pebody R. Effectiveness of trivalent and pandemic influenza vaccines in England and Wales 2008-2010: results from a cohort study in general practice. Vaccine 2012; 30:1371–8. DOI: 10.1016/j.vaccine.2011.12.038
325. Helmeke C, Grafe L, Irmscher HM, Gottschalk C, Karagiannis I, and Oppermann H. Effectiveness of the 2012/13 trivalent live and inactivated influenza vaccines in children and adolescents in Saxony-Anhalt, Germany: a test-negative case-control study. PLoS One 2015; 10:e0122910. DOI: 10.1371/journal.pone.0122910
326. Hermann N. [Effectiveness of live attenuated influenza vaccines and trivalent inactivated influenza vaccines against confirmed influenza in children and adolescents in Saxony-Anhalt, 2012/13]. Gesundheitswesen 2015; 77:499–501. DOI: 10.1055/s-0035-1554685
327. Hood N, Flannery B, Gaglani M, et al. Influenza vaccine effectiveness among children: 2011-2020. Pediatrics 2023; 151. DOI: 10.1542/peds.2022-059922
328. Hu W, DeMarcus LS, Sjoberg PA, and Robbins AS. Inactivated influenza vaccine effectiveness among department of defense beneficiaries aged 6 months-17 years, 2016-2017 through 2019-2020 influenza seasons. PLoS One 2021; 16:e0256165. DOI: 10.1371/journal.pone.0256165
329. Jain VK, Rivera L, Zaman K, et al. Vaccine for prevention of mild and moderate-to-severe influenza in children. N. Engl. J. Med. 2013; 369:2481–91. DOI: 10.1056/NEJMoa1215817
330. Janjua NZ, Skowronski DM, De Serres G, et al. Estimates of influenza vaccine effectiveness for 2007-2008 from Canada's sentinel surveillance system: cross-protection against major and minor variants. J. Infect. Dis 2012; 205:1858–68. DOI: 10.1093/infdis/jis283
331. Joshi AY, Iyer VN, St Sauver JL, Jacobson RM, and Boyce TG. Effectiveness of inactivated influenza vaccine in children less than 5 years of age over multiple influenza seasons: a case-control study. Vaccine 2009; 27:4457–61. DOI: 10.1016/j.vaccine.2009.05.038
332. Kafatos G, Pebody R, Andrews N, Durnall H, Barley M, and Fleming D. Effectiveness of seasonal influenza vaccine in preventing medically attended influenza infection in England and Wales during the 2010/2011 season: a primary care-based cohort study. Influenza Other Respir. Viruses 2013; 7:1175–80. DOI: 10.1111/irv.12163
333. Kang YK, Oh HL, Lim JS, et al. Evaluation of the field-protective effectiveness of seasonal influenza vaccine among Korean children aged < 5 years during the 2014-2015 and 2015-2016 influenza seasons: a cohort study. Hum. Vaccin. Immunother. 2019; 15:481–6. DOI: 10.1080/21645515.2018.1528832
334. Kao CM, Lai K, McAteer JM, et al. Influenza vaccine effectiveness and disease burden in children and adolescents with sickle cell disease: 2012-2017. Pediatr. Blood Cancer. 2020; 67:e28358. DOI: 10.1002/pbc.28358
335. Kelly HA, Grant KA, Fielding JE, Carville KS, Looker CO, Tran T, and Jacoby P. Pandemic influenza H1N1 2009 infection in Victoria, Australia: no evidence for harm or benefit following receipt of seasonal influenza vaccine in 2009. Vaccine 2011; 29:6419–26. DOI: 10.1016/j.vaccine.2011.03.055
336. Kelly H, Jacoby P, Dixon GA, et al. Vaccine effectiveness against laboratory-confirmed influenza in healthy young children: a case-control study. Pediatr. Infect. Dis. J. 2011; 30:107–11. DOI: 10.1097/INF.0b013e318201811c
337. Kim SY, Kim NH, Eun BW, et al. Protective field efficacy study of influenza vaccines for Korean children and adolescent in 2010-2011 season. Korean J Pediatr Infect Dis 2012; 19:149–56. DOI: 10.14776/kjpid.2012.19.3.149
338. Kim SS, Naioti EA, Halasa NB, et al. Vaccine effectiveness against influenza hospitalization and emergency department visits in 2 A(H3N2) dominant influenza seasons among children <18 years old-new Vaccine Surveillance Network 2016-2017 and 2017-2018. J. Infect. Dis 2022; 226:91–6. DOI: 10.1093/infdis/jiab624
339. Kimiya T, Shinjoh M, Anzo M, Takahashi H, Sekiguchi S, Sugaya N, and Takahashi T. Effectiveness of inactivated quadrivalent influenza vaccine in the 2015/2016 season as assessed in both a

test-negative case-control study design and a traditional case-control study design. Eur. J. Pediatr. 2018; 177:1009–17. DOI: 10.1007/s00431-018-3145-7
340. Kittikraisak W, Suntarattiwong P, Ditsungnoen D, et al. Effectiveness of the 2013 and 2014 southern hemisphere influenza vaccines against laboratory-confirmed influenza in young children using a test-negative design, Bangkok, Thailand. Pediatr. Infect. Dis. J. 2016; 35:318–25. DOI: 10.1097/INF.0000000000001280
341. Larrauri A, Savulescu C, Jimenez-Jorge S, et al. Influenza pandemic (H1N1) 2009 activity during summer 2009. Effectiveness of the 2008-9 trivalent vaccine against pandemic influenza in Spain. Gac. sanit. 2011; 25:23–8. DOI: 10.1016/j.gaceta.2010.06.010
342. S.-L. L, Kwan MYW, Murphy C, et al. Influenza vaccine effectiveness against influenza-associated hospitalizations in children, Hong Kong, November 2023 to June 2024. medRxiv 2024. DOI: 10.1101/2024.08.30.24312831
343. Leung TF, Chan RWY, Kwok A, et al. School-based surveillance for influenza vaccine effectiveness during 2014-2015 seasons in Hong Kong. Influenza Other Respir. Viruses 2017; 11:319–27. DOI: 10.1111/irv.12455
344. Leung TF, Chan RWY, Kwok A, et al. School-based surveillance for childhood influenza in Hong Kong, 2014-15. Hong Kong J. Paediatr. 2018; 23:99–100
345. Levy A, Sullivan SG, Tempone SS, et al. Influenza vaccine effectiveness estimates for Western Australia during a period of vaccine and virus strain stability, 2010 to 2012. Vaccine 2014; 32:6312–8. DOI: 10.1016/j.vaccine.2014.08.066
346. Levy JW, Simasathien S, Watanaveeradej V, et al. Influenza vaccine effectiveness in the tropics: Moderate protection in a surveillance population in Bangkok between august 2009 and january 2013. Am. J. Trop. Med. Hyg. 2014; 91:539
347. Levy JW, Simasathien S, Watanaveeradej V, et al. Influenza vaccine effectiveness in the tropics: moderate protection in a case test-negative analysis of a hospital-based surveillance population in Bangkok between August 2009 and January 2013. PLoS One 2015; 10:e0134318. DOI: 10.1371/journal.pone.0134318
348. Li-Kim-Moy JP, Yin JK, Heron L, et al. Influenza vaccine efficacy in young children attending childcare: A randomised controlled trial. J. Paediatr. Child Health. 2017; 53:47–54. DOI: 10.1111/jpc.13313
349. Luo SY, Zhu JL, Lyu MZ, et al. [Evaluation of the influenza vaccine effectiveness among children aged 6 to 72 months based on the test-negative case control study design]. Xian Dai Yu Fang Yi Xue 2019; 53:576–80. DOI: 10.3760/cma.j.issn.0253-9624.2019.06.007
350. Maeda T, Shintani Y, Nakano K, Terashima K, and Yamada Y. Failure of inactivated influenza A vaccine to protect healthy children aged 6-24 months. Pediatr. Int. 2004; 46:122–5
351. Malosh RE, Petrie JG, Callear A, et al. Effectiveness of influenza vaccines in the HIVE household cohort over 8 years: is there evidence of indirect protection?. Clin. Infect. Dis. 2021; 73:1248–56. DOI: 10.1093/cid/ciab395
352. Marron L, McKenna A, O'Donnell J, Joyce M, Bennett C, Connell J, and Domegan L. Influenza vaccine effectiveness against symptomatic influenza in primary care: a test negative case control study over two influenza seasons 2022/2023 and 2023/2024 in Ireland. Influenza Other Respir. Viruses 2024; 18:e70023. DOI: 10.1111/irv.70023
353. Matsumoto K, Fukushima W, Morikawa S, et al. Influence of prior influenza vaccination on current influenza vaccine effectiveness in children aged 1 to 5 years. Vaccines 2021; 9. DOI: 10.3390/vaccines9121447
354. McLean HQ, Thompson MG, Sundaram ME, et al. Influenza vaccine effectiveness in the United States during 2012-2013: variable protection by age and virus type. J. Infect. Dis 2015; 211:1529–40. DOI: 10.1093/infdis/jiu647
355. Mi J, Wang J, Chen L, et al. Real-world effectiveness of influenza vaccine against medical-attended influenza infection during 2023/24 season in Ili Kazakh Autonomous Prefecture, China: A test-negative, case-control study. Hum. Vaccines Immunother. 2024; 20:2394255. DOI: 10.1080/21645515.2024.2394255
356. Mohl A, Grafe L, Helmeke C, Ziehm D, Monazahian M, Irmscher HM, and Dreesman J. Estimating vaccine effectiveness against laboratory-confirmed influenza among children and adolescents in Lower Saxony and Saxony-Anhalt, 2012-2016. Epidemiol. Infect. 2018; 146:78–88. DOI: 10.1017/S0950268817002709
357. Mori M, Hasegawa J, Showa S, Matsushima A, Ohnishi H, Yoto Y, and Tsutsumi H. Effectiveness of influenza vaccine in children in day-care centers of Sapporo. Pediatr. Int. 2014; 56:53–6. DOI: 10.1111/ped.12221

358. Murphy C, Kwan MYW, Chan ELY, et al. Influenza vaccine effectiveness against hospitalizations associated with influenza A(H3N2) in Hong Kong children aged 9 months to 17 years, June-November 2023. Vaccine 2024; 42:1878EP–1882. DOI: 10.1016/j.vaccine.2024.02.056
359. Nakamura K and Okuno Y. Field trials of live influenza vaccine carried out in november. ii. prophylactic effect of live influenza vaccine (Japanese). Jpn J. Bacteriol. 1966; 21:78–84
360. Ng S, Ip DKM, Fang VJ, et al. The effect of age and recent influenza vaccination history on the immunogenicity and efficacy of 2009-10 seasonal trivalent inactivated influenza vaccination in children. PLoS One 2013; 8:e59077. DOI: 10.1371/journal.pone.0059077
361. Niang MN, Sugimoto JD, Diallo A, et al. Estimates of inactivated influenza vaccine effectiveness among children in Senegal: results from 2 consecutive cluster-randomized controlled trials in 2010 and 2011. Clin. Infect. Dis. 2021; 72:e959–e969. DOI: 10.1093/cid/ciaa1689
362. Ohmit SE, Petrie JG, Malosh RE, Cowling BJ, Thompson MG, Shay DK, and Monto AS. Influenza vaccine effectiveness in the community and the household. Clin. Infect. Dis. 2013; 56:1363–9. DOI: 10.1093/cid/cit060
363. Ohmit SE, Thompson MG, Petrie JG, et al. Influenza vaccine effectiveness in the 2011-2012 season: protection against each circulating virus and the effect of prior vaccination on estimates. Clin. Infect. Dis. 2014; 58:319–27. DOI: 10.1093/cid/cit736
364. Ohmit SE, Petrie JG, Malosh RE, Fry AM, Thompson MG, and Monto AS. Influenza vaccine effectiveness in households with children during the 2012-2013 season: assessments of prior vaccination and serologic susceptibility. J. Infect. Dis 2015; 211:1519–28. DOI: 10.1093/infdis/jiu650
365. Ohmit SE, Petrie JG, Malosh RE, et al. Substantial influenza vaccine effectiveness in households with children during the 2013-2014 influenza season, when 2009 pandemic influenza A(H1N1) virus predominated. J. Infect. Dis 2016; 213:1229–36. DOI: 10.1093/infdis/jiv563
366. Olson SM, Newhams MM, Halasa NB, et al. Vaccine effectiveness against life-threatening influenza illness in US children. Clin. Infect. Dis. 2022; 75:230–8. DOI: 10.1093/cid/ciab931
367. Omer I, Rosenberg A, Sefty H, et al. Lineage-matched versus mismatched influenza B vaccine effectiveness following seasons of marginal influenza B circulation. Vaccine 2022; 40:880–5. DOI: 10.1016/j.vaccine.2021.12.056
368. Orellano PW, Reynoso JI, Carlino O, and Uez O. Protection of trivalent inactivated influenza vaccine against hospitalizations among pandemic influenza A (H1N1) cases in Argentina. Vaccine 2010; 28:5288–91. DOI: 10.1016/j.vaccine.2010.05.051
369. Pebody RG, Andrews N, Fleming DM, et al. Age-specific vaccine effectiveness of seasonal 2010/2011 and pandemic influenza A(H1N1) 2009 vaccines in preventing influenza in the United Kingdom. Epidemiol. Infect. 2013; 141:620–30. DOI: 10.1017/S0950268812001148
370. Pebody RG, Andrews N, McMenamin J, et al. Vaccine effectiveness of 2011/12 trivalent seasonal influenza vaccine in preventing laboratory-confirmed influenza in primary care in the United Kingdom: evidence of waning intra-seasonal protection. Euro. Surveill. 2013; 18
371. Pebody R, Warburton F, Andrews N, et al. Effectiveness of seasonal influenza vaccine in preventing laboratory-confirmed influenza in primary care in the United Kingdom: 2014/15 end of season results. Euro. Surveill. 2015; 20. DOI: 10.2807/1560-7917.ES.2015.20.36.30013
372. Pebody R, Sile B, Warburton F, et al. Live attenuated influenza vaccine effectiveness against hospitalisation due to laboratory-confirmed influenza in children two to six years of age in England in the 2015/16 season. Euro. Surveill. 2017; 22. DOI: 10.2807/1560-7917.ES.2017.22.4.30450
373. Pebody RG, Zhao H, Whitaker HJ, Ellis J, Donati M, Zambon M, and Andrews N. Effectiveness of influenza vaccine in children in preventing influenza associated hospitalisation, 2018/19, England. Vaccine 2020; 38:158–64. DOI: 10.1016/j.vaccine.2019.10.035
374. Perez-Gimeno G, Mazagatos C, Lorusso N, et al. Effectiveness of influenza vaccines in children aged 6 to 59months: a test-negative case-control study at primary care and hospital level, Spain 2023/24. Euro. Surveill. 2024; 29. DOI: 10.2807/1560-7917.ES.2024.29.40.2400618
375. Petrie JG, Malosh RE, Cheng CK, et al. The household influenza vaccine effectiveness study: lack of antibody response and protection following receipt of 2014-2015 influenza vaccine. Clin. Infect. Dis. 2017; 65:1644–51. DOI: 10.1093/cid/cix608
376. Powell L and Begue RE. Estimate of the effectiveness of influenza vaccine among children for the 2017-2018 season. J. Investig. Med. 2019; 67:598. DOI: 10.1136/jim-2018-000974.623
377. Powell LN and Begue RE. Influenza vaccine effectiveness among children for the 2017-2018 season. Pediatr. Infect. Dis. J. 2020; 9:468–73. DOI: 10.1093/jpids/piz077

378. Price AM, Flannery B, Talbot HK, et al. Influenza vaccine effectiveness against influenza A(H3N2)-related illness in the United States during the 2021-2022 influenza season. Clin. Infect. Dis. 2023; 76:1358–63. DOI: 10.1093/cid/ciac941
379. Puig-Barbera J, Mira-Iglesias A, Burtseva E, et al. Influenza epidemiology and influenza vaccine effectiveness during the 2015-2016 season: results from the Global Influenza Hospital Surveillance Network. BMC Infect. Dis. 2019; 19:415. DOI: 10.1186/s12879-019-4017-0
380. Qin Y, Zhang Y, Wu P, et al. Influenza vaccine effectiveness in preventing hospitalization among Beijing residents in China, 2013-15. Vaccine 2016; 34:2329–33. DOI: 10.1016/j.vaccine.2016.03.068
381. Rolfes MA, Flannery B, Chung JR, et al. Effects of influenza vaccination in the United States during the 2017-2018 onfluenza season. Clin. Infect. Dis. 2019; 69:1845–53. DOI: 10.1093/cid/ciz075
382. Sahni LC, Naioti EA, Olson SM, et al. Sustained Within-season Vaccine Effectiveness Against Influenza-associated Hospitalization in Children: Evidence From the New Vaccine Surveillance Network, 2015-2016 Through 2019-2020. Clin. Infect. Dis. 2023; 76:e1031–e1039. DOI: 10.1093/cid/ciac577
383. Segaloff HE, Leventer-Roberts M, Riesel D, et al. Influenza vaccine effectiveness against hospitalization in fully and partially vaccinated children in Israel: 2015-2016, 2016-2017, and 2017-2018. Clin. Infect. Dis. 2019; 69:2153–61. DOI: 10.1093/cid/ciz125
384. Shinjoh M, Furuichi M, Kobayashi H, et al. Trends in effectiveness of inactivated influenza vaccine in children by age groups in seven seasons immediately before the COVID-19 era. Vaccine 2022; 40:3018–26. DOI: 10.1016/j.vaccine.2022.04.033
385. Shinjoh M, Furuichi M, Tsuzuki S, et al. Effectiveness of inactivated influenza and COVID-19 vaccines in hospitalized children in 2022/23 season in Japan - the first season of co-circulation of influenza and COVID-19. Vaccine 2023; 41:4777–81. DOI: 10.1016/j.vaccine.2023.06.082
386. Shinjoh M, Yaginuma M, Yamaguchi Y, et al. Effectiveness of inactivated influenza vaccine in children during the 2023/24 season: the first season after relaxation of intensive COVID-19 measures. Vaccine 2024; 42:126241. DOI: 10.1016/j.vaccine.2024.126241
387. Simpson CR, Lone NI, Kavanagh K, et al. Vaccine effectiveness of live attenuated and trivalent inactivated influenza vaccination in 2010/11 to 2015/16: the SIVE II record linkage study. Health technology assessment (Winchester, England) 2020; 24:1–66. DOI: 10.3310/hta24670
388. Smolarchuk C, Ickert C, Zelyas N, Kwong JC, and Buchan SA. Early influenza vaccine effectiveness estimates using routinely collected data, Alberta, Canada, 2023/24 season. Euro. Surveill. 2024; 29. DOI: 10.2807/1560-7917.ES.2024.29.2.2300709
389. Sominina A, Danilenko D, Komissarov A, et al. Age-specific etiology of severe acute respiratory infections and influenza vaccine effectivity in prevention of hospitalization in Russia, 2018-2019 season. J Epidemiol Glob. Hea. 2021; 11:413–25. DOI: 10.1007/s44197-021-00009-1
390. Stein Y, Mandelboim M, Sefty H, Pando R, Mendelson E, Shohat T, and Glatman-Freedman A. Seasonal influenza vaccine effectiveness in preventing laboratory-confirmed influenza in primary care in Israel, 2016-2017 season: insights into novel age-specific analysis. Clin. Infect. Dis. 2018; 66:1383–91. DOI: 10.1093/cid/cix1013
391. Stuurman AL, Biccler J, Carmona A, et al. Brand-specific influenza vaccine effectiveness estimates during 2019/20 season in Europe - results from the DRIVE EU study platform. Vaccine 2021; 39:3964–73. DOI: 10.1016/j.vaccine.2021.05.059
392. Stuurman AL, Carmona A, Biccler J, et al. Brand-specific estimates of influenza vaccine effectiveness for the 2021-2022 season in Europe: results from the DRIVE multi-stakeholder study platform. Public Health Front. 2023; 11:1195409. DOI: 10.3389/fpubh.2023.1195409
393. Su WJ, Chan TC, Chuang PH, Liu YL, Lee PI, Liu MT, and Chuang JH. Estimating influenza vaccine effectiveness using routine surveillance data among children aged 6-59 months for five consecutive influenza seasons. Int. J. Infect. Dis. 2015; 30:115–21. DOI: 10.1016/j.ijid.2014.11.011
394. Sugaya N, Nerome K, Ishida M, Matsumoto M, Mitamura K, and Nirasawa M. Efficacy of inactivated vaccine in preventing antigenically drifted influenza type A and well-matched type B. JAMA 1994; 272:1122–6
395. Sugaya N, Shinjoh M, Kawakami C, et al. Trivalent inactivated influenza vaccine effective against influenza A(H3N2) variant viruses in children during the 2014/15 season, Japan. Euro. Surveill. 2016; 21. DOI: 10.2807/1560-7917.ES.2016.21.42.30377
396. Sugaya N, Shinjoh M, Nakata Y, et al. Three-season effectiveness of inactivated influenza vaccine in preventing influenza illness and hospitalization in children in Japan, 2013-2016. Vaccine 2018; 36:1063–71. DOI: 10.1016/j.vaccine.2018.01.024

397. Sullender WM, Fowler KB, Gupta V, et al. Efficacy of inactivated trivalent influenza vaccine in rural India: a 3-year cluster-randomised controlled trial. Lancet Glob. Health 2019; 7:e940–e950. DOI: 10.1016/S2214-109X(19)30079-8
398. Sumner KM, Sahni LC, Boom JA, et al. Estimated vaccine effectiveness for pediatric patients With severe influenza, 2015-2020. JAMA Netw. Open 2024; 7:e2452512. DOI: 10.1001/jamanetworkopen.2024.52512
399. Sun Y, Shi W, Zhang D, et al. Early vaccine effectiveness estimates against medically attended laboratory-confirmed influenza based on influenza surveillance, Beijing, China, 2024/25 season. Euro. Surveill. 2025; 30. DOI: 10.2807/1560-7917.ES.2025.30.7.2500084
400. Suzuki T, Ono Y, Maeda H, et al. Effectiveness of trivalent influenza vaccine among children in two consecutive seasons in a community in Japan. Tohoku J. Exp. Med. 2014; 232:97–104
401. Switzer C, Verschoor CP, Pullenayegum E, Singh P, and Loeb M. Relative Antibody Mediated Protection against Influenza in Vaccinated Children. Available at SSRN 4173967 2022
402. Tenforde MW, Kondor RJG, Chung JR, et al. Effect of antigenic drift on influenza vaccine effectiveness in the United States-2019-2020. Clin. Infect. Dis. 2021; 73:e4244–e4250. DOI: 10.1093/cid/ciaa1884
403. Tenforde MW, Reeves EL, Weber ZA, et al. Influenza vaccine effectiveness against hospitalizations and emergency department or urgent care encounters for children, adolescents, and adults during the 2023-2024 season, United States. Clin. Infect. Dis. 2024. DOI: 10.1093/cid/ciae597
404. Teros-Jaakkola T, Toivonen L, Schuez-Havupalo L, Karppinen S, Julkunen I, Waris M, and Peltola V. Influenza virus infections from 0 to 2 years of age: A birth cohort study. J. Microbiol. Immunol. Infect. 2019; 52:526–33. DOI: 10.1016/j.jmii.2017.10.007
405. Turner N, Pierse N, Bissielo A, Huang QS, Baker MG, Widdowson MA, and Kelly H. The effectiveness of seasonal trivalent inactivated influenza vaccine in preventing laboratory confirmed influenza hospitalisations in Auckland, New Zealand in 2012. Vaccine 2014; 32:3687–93. DOI: 10.1016/j.vaccine.2014.04.013
406. Valdin HL and Begue RE. Influenza vaccines effectiveness 2013-14 through 2015-16, a test-negative study in children. Vaccine 2017; 35:4088–93. DOI: 10.1016/j.vaccine.2017.06.050
407. Wang Y, Chen L, Cheng Y, et al. Potential impact of B lineage mismatch on trivalent influenza vaccine effectiveness during the 2015-2016 influenza season among nursery school children in Suzhou, China. Hum. Vaccin. Immunother. 2018; 14:630–6. DOI: 10.1080/21645515.2017.1397868
408. Wang Y, Chen L, Yu J, Pang Y, Zhang J, Zhang T, and Zhao G. The effectiveness of influenza vaccination among nursery school children in China during the 2016/17 influenza season. Vaccine 2018; 36:2456–61. DOI: 10.1016/j.vaccine.2018.03.039
409. Wang CY, Chang YH, Huang LM, et al. Effects of influenza vaccine and sun exposure time against laboratory-confirmed influenza hospitalizations among young children during the 2012-13 to 2015-16 influenza seasons. J. Microbiol. Immunol. Infect. 2019; 52:880–7. DOI: 10.1016/j.jmii.2019.09.010
410. Widgren K, Magnusson M, Hagstam P, et al. Prevailing effectiveness of the 2009 influenza A(H1N1)pdm09 vaccine during the 2010/11 season in Sweden. Euro. Surveill. 2013; 18:20447
411. Wu S, Pan Y, Zhang X, et al. Influenza vaccine effectiveness in preventing laboratory-confirmed influenza in outpatient settings: a test-negative case-control study in Beijing, China, 2016/17 season. Vaccine 2018; 36:5774–80. DOI: 10.1016/j.vaccine.2018.07.077
412. Yamaguchi S, Ohfuji S, and Hirota Y. Influenza vaccine effectiveness in primary school children in Japan: a prospective cohort study using rapid diagnostic test results. J. Infect. Chemother. 2010; 16:407–13. DOI: 10.1007/s10156-010-0070-8
413. Yang S, Li T, Long J, et al. Effectiveness of influenza vaccine among the population in Chongqing, China, 2018-2022: a test negative design-based evaluation. Hum. Vaccines Immunother. 2024; 20:2376821. DOI: 10.1080/21645515.2024.2376821
414. Zeno EE, Nogareda F, Regan A, et al. Interim effectiveness estimates of 2024 southern hemisphere influenza vaccines in preventing influenza-associated hospitalization - REVELAC-i Network, five South American countries, March-July 2024. MMWR Morb. Mortal Wkly. Rep. 2024; 73:861EP–868. DOI: 10.15585/mmwr.mm7339a1
415. Zhang Y, Wu P, Feng L, et al. Influenza vaccine effectiveness against influenza-associated hospitalization in 2015/16 season, Beijing, China. Vaccine 2017; 35:3129–34. DOI: 10.1016/j.vaccine.2017.03.084
416. Zimmerman RK, Nowalk MP, Chung J, et al. 2014-2015 influenza vaccine effectiveness in the United States by vaccine type. Clin. Infect. Dis. 2016; 63:1564–73

417. Abraham C, Chen Q, Fan W, and Stockwell MS. Association of seasonal severity and vaccine effectiveness with influenza vaccination rates in children. JAMA Pediatr. 2020; 174:86–8. DOI: 10.1001/jamapediatrics.2019.4221
418. Ahmed M, Dayman NA, Claydon A, Gaillard E, and Tang JW. Influenza vaccine effectiveness in children with cystic fibrosis. Pediatr. Pulmonol. 2019; 54:273–4. DOI: 10.1002/ppul.22495
419. Ambrose CS, Wu X, and Belshe RB. The efficacy of live attenuated and inactivated influenza vaccines in children as a function of time postvaccination. Pediatr. Infect. Dis. J. 2010; 29:806–11. DOI: 10.1097/INF.0b013e3181e2872f
420. Amer A, Fischer H, Li X, and Asmar B. Possible impact of yearly childhood vaccination with trivalent inactivated influenza vaccine (TIV) on the immune response to the pandemic strain H1n1. Cogent Med. 2017; 4. DOI: 10.1080/2331205X.2017.1408251
421. Ashkenazi S, Vertruyen A, Aristegui J, et al. Superior relative efficacy of live attenuated influenza vaccine compared with inactivated influenza vaccine in young children with recurrent respiratory tract infections. Pediatr. Infect. Dis. J. 2006; 25:870–9
422. Bakkaloglu SA, Ozdemir Y, Melek E, et al. Influenza and pneumococcus vaccination rates in pediatric dialysis patients in Europe. Pediatr. Nephrol. 2016; 31:1932. DOI: 10.1007/s00467-016-3467-5
423. Bandell A, Mallory R, and Ambrose CS. Real-world effectiveness of inactivated and live attenuated influenza vaccines in children during three recent seasons: 2016-2019. Open Forum Infect. Dis. 2020; 7:S709. DOI: 10.1093/ofid/ofaa439.1583
424. Baum U, Auranen K, Kulathinal S, Syrjanen R, Nohynek H, and Jokinen J. Cohort study design for estimating the effectiveness of seasonal influenza vaccines in real time based on register data: the Finnish example. Scand. J. Public Health 2020; 48:316–22. DOI: 10.1177/1403494818808635
425. Bekkat-Berkani R, Ray R, Jain VK, Chandrasekaran V, and Innis BL. Evidence update: GlaxoSmithKline's inactivated quadrivalent influenza vaccines. Expert Rev. Vaccines 2016; 15:201–14. DOI: 10.1586/14760584.2016.1113878
426. Belongia EA, Sundaram ME, McClure DL, Meece JK, Ferdinands J, and VanWormer JJ. Waning vaccine protection against influenza A (H3N2) illness in children and older adults during a single season. Vaccine 2015; 33:246–51. DOI: 10.1016/j.vaccine.2014.06.052
427. Belshe RB, Gruber WC, Mendelman PM, et al. Correlates of immune protection induced by live, attenuated, cold-adapted, trivalent, intranasal influenza virus vaccine. J. Infect. Dis 2000; 181:1133–7
428. Belshe RB, Edwards KM, Vesikari T, et al. Live attenuated versus inactivated influenza vaccine in infants and young children. N. Engl. J. Med. 2007; 356:685–96
429. Belshe RB, Ambrose CS, and Yi T. Safety and efficacy of live attenuated influenza vaccine in children 2-7 years of age. Vaccine 2008; 26 Suppl 4:10–6. DOI: 10.1016/j.vaccine.2008.06.083
430. Belshe RB. The potential of live, attenuated influenza vaccine for the prevention of influenza in children. Clin. Infect. Dis. 2019; 69:795–6. DOI: 10.1093/cid/ciy1007
431. Campbell AP, Ogokeh CE, McGowan C, et al. Influenza vaccine effectiveness against laboratory-confirmed influenza in children hospitalized with respiratory illness in the United States, 2016-2017 and 2017-2018 seasons. Open Forum Infect. Dis. 2019; 6:S26–S27. DOI: 10.1093/ofid/ofz359.058
432. Cantarutti A, Barbieri E, Didone F, Corrao G, and Giaquinto C. Effectiveness of influenza vaccination in healthy children: a population-based investigation. Pharmacoepidemiol. Drug Saf. 2021; 30:89. DOI: 10.1002/pds.5305
433. Carvalho LM, Paula FE, Silvestre RVD, Roberti LR, Mello WA, Arruda E, and Ferriani VPL. Acute respiratory infections, influenza-like illness and JIA: Impact on disease activity and response to the influenza vaccine. Pediatr. Rheumatol. 2012; 10
434. Chung JR, Flannery B, Ambrose CS, et al. Live attenuated and inactivated influenza vaccine effectiveness. Pediatrics 2019; 143. DOI: 10.1542/peds.2018-2094
435. Collignon P, Doshi P, Del Mar C, and Jefferson T. Safety and efficacy of inactivated influenza vaccines in children. Clin. Infect. Dis. 2015; 60:489. DOI: 10.1093/cid/ciu835
436. Cowling BJ and Chiu SS. Efficacy of inactivated influenza vaccines in young children. Lancet Child Adolesc. Health. 2018; 2:307–8. DOI: 10.1016/S2352-4642(18)30074-9
437. Cowling BJ, Lim WW, Perera RAPM, Fang VJ, Leung GM, Peiris JSM, and Tchetgen Tchetgen EJ. Influenza hemagglutination-inhibition antibody titer as a mediator of vaccine-induced protection for influenza B. Clin. Infect. Dis. 2019; 68:1713–7. DOI: 10.1093/cid/ciy759
438. Da Cas R, Traversa G, Santuccio C, Trotta F, and Menniti-Ippolito F. Influenza vaccine effectiveness against severe cases in children: Results from two influenza seasons. Drug Safety 2013; 36:891. DOI: 10.1007/s40264-013-0087-x

439. Dierig A, Heron LG, Lambert SB, et al. Epidemiology of respiratory viral infections in children enrolled in a study of influenza vaccine effectiveness. Influenza Other Respir. Viruses 2014; 8:293–301. DOI: 10.1111/irv.12229
440. Divino V, Postma M, Pelton SI, Mould-Quevedo JF, Anupindi R, DeKoven M, and Levin MJ. Relative vaccine effectiveness against influenza-related and any respiratory-related hospital encounter during the 2019/20 high influenza activity period: a comprehensive real-world analysis to compare quadrivalent cell-based and egg-based influenza vaccines. Open Forum Infect. Dis. 2021; 8:S61. DOI: 10.1093/ofid/ofab466.096
441. Domorazkova E, Zavadova H, and Bergmannova V. Immunization against influenza in children by the Czechoslovak sub-unit vaccine. Ces.-Slov. Pediatrie 1981; 36:100–2
442. Fowlkes A, Friedlander H, Stefens A, et al. Vaccine effectiveness against influenza-associated hospitalization among children aged < 13 years using a hospital-based surveillance system in minnesota, 2013-2016. Open Forum Infect. Dis. 2017; 4:S60. DOI: 10.1093/ofid/ofx162.142
443. Fukushima W, Morikawa S, Fujioka M, et al. Influenza vaccine effectiveness in young Japanese children over five seasons. Int. J. Epidemiol. 2021; 50:i76. DOI: 10.1093/ije/dyab168.209
444. Hamrin J, Bennet R, Eriksson M, Lind G, and Ortqvist E. Effectiveness of a mono-valent adjuvanted vaccine (Pandemrix) for the prevention of hospitalisation for influenza a (H1N1)V infection in children. Acta Paediatr. 2010; 99:85. DOI: 10.1111/{\%}28ISSN{\%}291651-2227
445. Herron A. Dettleff G. Hixon B. et al. Influenza vaccination in patients with rheumatic diseases. Safety and efficacy. JAMA 1979; 242:53–6. DOI: 10.1001/jama.242.1.53
446. Homaira N, Fathima P, Lim FJ, et al. Influenza vaccine uptake and its effectiveness in preventing hospitalisations among Australian children with chronic lung diseases. Int. J. Infect. Dis. 2020; 101:465–6. DOI: 10.1016/j.ijid.2020.09.1220
447. Isaacs D. Inactivated influenza vaccine highly effective in young and immunocompromised children. J. Paediatr. Child Health 2016; 52:784. DOI: 10.1111/jpc.13259
448. Jennings LC, MacDiarmid RD, and Miles JA. A study of acute respiratory disease in the community of Port Chalmers. III. Efficacy of influenza virus subunit vaccines in 1973, 1974 and 1975. N. Z. Med. J. 1980; 92:230–3
449. Jing-Xia G, Yu-Liang Z, Jin-Feng L, Shu-Zhen L, Guo-Yang L, and Qi L. Safety and effectiveness assessment of 2011-2012 seasonal influenza vaccine produced in China: a randomized trial. Postgrad. Med. 2017; 129:907–14. DOI: 10.1080/00325481.2017.1369133
450. Karras NA, Weeres M, Sessions W, et al. A randomized trial of one versus two doses of influenza vaccine after allogeneic transplantation. Biol. Blood Marrow Transplant 2013; 19:109–16. DOI: 10.1016/j.bbmt.2012.08.015
451. Keitel WA. Repeated immunization of children with inactivated and live attenuated influenza virus vaccines: safety, immunogenicity, and protective efficacy. Semin. Pediatr. Infect. Dis. 2002; 13:112–9
452. Kittikraisak W, Suntarattiwong P, Levy J, Fernandez S, Dawood FS, Olsen SJ, and Chotpitayasunondh T. Influenza vaccination coverage and effectiveness in young children in Thailand, 2011-2013. Influenza Other Respir. Viruses 2015; 9:85–93. DOI: 10.1111/irv.12302
453. Kolber MR, Lau D, Eurich D, and Korownyk C. Effectiveness of the trivalent influenza vaccine. Can. Fam. Physician 2014; 60:50
454. Ladva CN, Belongia E, Monto A, et al. Lack of influence of early exposure to influenza a(H3N2) viruses on vaccine effectiveness against a(H3N2)-associated illness in us children <18 years, 2016-2018. Open Forum Infect. Dis. 2019; 6:S962. DOI: 10.1093/ofid/ofz360.2412
455. Loeb M, Russell M, Moss L, et al. Efficacy of influenza vaccine for preventing symptomatic illness given infection: results from a cluster randomized trial in Hutterite colonies. Can. J. Infect. Dis. Med. Microbiol. 2010; 21:180
456. Longini IM, Halloran ME, Nizam A, Wolff M, Mendelman PM, Fast PE, and Belshe RB. Estimation of the efficacy of live, attenuated influenza vaccine from a two-year, multi-center vaccine trial: implications for influenza epidemic control. Vaccine 2000; 18:1902–9
457. Luna B. Confounding factors and effectiveness of influenza vaccine among young children. Int. J. Pediatr. 2009; 24:169–70
458. Maeda T, Shintani Y, Miyamoto H, Kawagoe H, Nakano K, Nishiyama A, and Yamada Y. Prophylactic effect of inactivated influenza vaccine on young children. Pediatr. Int. 2002; 44:43–6
459. Mahmud SM, Bozat-Emre S, Thompson LH, Elliott LJ, and Caeseele P. Effectiveness of the pandemic H1N1 influenza vaccines in preventing hospitalization for influenza and pneumonia: a population-based case-control study. Pharmacoepidemiol. Drug Saf. 2013; 22:438. DOI: 10.1002/pds.3512

460. McLean HQ, Caspard H, Griffin MR, et al. Impact of prior vaccination history on risk of vaccine failure with live attenuated and inactivated influenza vaccines in children, 2013-14 through 2015-16. Open Forum Infect. Dis. 2017; 4:S60–S61. DOI: 10.1093/ofid/ofx162.143
461. Mendelman PM, Cordova J, and Cho I. Safety, efficacy and effectiveness of the influenza virus vaccine, trivalent, types A and B, live, cold-adapted (CAIV-T) in healthy children and healthy adults. Vaccine 2001; 19:2221–6
462. Motaghi S, Pullenayegum E, Morgan RL, and Loeb M. The role of influenza hemagglutination-inhibition antibody as a vaccine mediator in children. Vaccine 2024; 42:126122. DOI: 10.1016/j.vaccine.2024.07.023
463. I NT, Zilisteanu E, and Alexandrescu V. Efficacy of an adsorbed trivalent split influenza vaccine administered by intradermal route. Arch. Roum. Pathol. Exp. Microbiol. 1981; 40:67–70
464. Nolan T, Roy-Ghanta S, Montellano M, et al. Relative efficacy of AS03-adjuvanted pandemic influenza A(H1N1) vaccine in children: results of a controlled, randomized efficacy trial. J. Infect. Dis 2014; 210:545–57. DOI: 10.1093/infdis/jiu173
465. Ochiai H, Shibata M, Kamimura K, and Niwayama S. Evaluation of the efficacy of split-product trivalent A(H1N1), A(H3N2), and B influenza vaccines: protective efficacy. Microbiol. Immunol. 1986; 30:1151–65
466. Pelton SI, Postma M, Divino V, Mould-Quevedo JF, Anupindi R, DeKoven M, and Levin MJ. Relative vaccine effectiveness against influenza-related hospitalizations and respiratory events during the 2019/20 influenza season in U.S. children and adults. a real-world evidence comparison between quadrivalent cell-based and egg-based Influenza vaccines. Open Forum Infect. Dis. 2021; 8:S758. DOI: 10.1093/ofid/ofab466.1533
467. Petrie JG, Cheng CK, Malosh RE, Martin ET, Fry AM, and Monto AS. No evidence of influenza vaccine effectiveness against antigenically drifted influenza a (H3N2) viruses in a household cohort during the 2014-2015 influenza season. Open Forum Infect. Dis. 2016; 3. DOI: 10.1093/ofid/ofw172.662
468. Rao S, Lamb M, Moss A, and Asturias EJ. Does last season's influenza vaccination affect current season's vaccine effectiveness in young children? Open Forum Infect. Dis. 2019; 6:S961. DOI: 10.1093/ofid/ofz360.2409
469. Regan AK, Arriola CS, Couto P, et al. Severity of influenza illness by seasonal influenza vaccination status among hospitalised patients in four South American countries, 2013-19: a surveillance-based cohort study. Lancet Infect. Dis. 2023; 23:222–32. DOI: 10.1016/S1473-3099(22)00493-5
470. Rose VL. Data show effectiveness of intranasal influenza vaccine in children. Am. Fam. Physician 1999; 59:2856
471. Schaad UB, Buhlmann U, Burger R, et al. Comparison of immunogenicity and safety of a virosome influenza vaccine with those of a subunit influenza vaccine in pediatric patients with cystic fibrosis. Antimicrob. Agents Chemother. 2000; 44:1163–7
472. Seeborg F, Li D, Chiang K, Thompson B, and Paul ME. The clinical effectiveness of influenza vaccination in HIV-infected children. J. Allergy Clin. Immunol. 2009; 123:S17
473. Shibata N and Urushihara H. Effectiveness of influenza vaccination for children in Japan. Pharmacoepidemiol. Drug Saf. 2015; 24:177–8. DOI: 10.1002/pds.3838
474. Singh H, Gupta G, and Tiwari P. Clinical effectiveness of the seasonal influenza vaccine in healthy Indian children. Value Health 2010; 13:A547–A548
475. Skowronski DM, G dS, Crowcroft NS, et al. Association between the 2008-09 seasonal influenza vaccine and pandemic H1N1 illness during spring-summer 2009: four observational studies from Canada. PLoS Med. 2010; 7. DOI: 10.1371/journal.pmed.1000258
476. Skowronski DM and Serres GD. Evidence in a cluster randomized controlled trial of increased 2009 pandemic risk associated with 2008–2009 seasonal influenza vaccine receipt. 2019. DOI: 10.1093/cid/ciz351
477. Tkaczyszyn K, Bazanow B, and Szenborn L. Effectiveness of vaccination against influenza in HIV-infected pediatric patients in the 2016-2017 epidemic season. Prz. Epidemiol. 2020; 74:667–75. DOI: 10.32394/pe.74.58
478. Tsai TF. MF59 adjuvanted seasonal and pandemic influenza vaccines. Yakugaku Zasshi 2011; 131:1733–41. DOI: http://dx.doi.org/10.1248/yakushi.131.1733
479. Uphoff H, Hauri AM, Schweiger B, Heckler R, Haas W, Gruber A, and Buchholz U. [Estimation of influenza vaccine effectiveness using routine surveillance data]. Bundesgesundheitsblatt Gesundheitsforschung Gesundheitsschutz 2006; 49:287–95
480. Vesikari T, Kirstein J, Devota Go G, et al. Efficacy, immunogenicity, and safety evaluation of an MF59-adjuvanted quadrivalent influenza virus vaccine compared with non-adjuvanted influenza

vaccine in children: a multicentre, randomised controlled, observer-blinded, phase 3 trial. Lancet Respir. Med. 2018; 6:345–56. DOI: 10.1016/S2213-2600(18)30108-5
481. Wagner AL, Gresh L, Sanchez N, et al. Influenza illness and partial vaccination in the first two years of life. Vaccines 2021; 9. DOI: 10.3390/vaccines9060676
482. Wat D, Gelder C, Hibbitts S, Bowler I, Pierrepoint M, Evans R, and Doull I. Is there a role for influenza vaccination in cystic fibrosis?. J. Cyst. Fibros. 2008; 7:85–8
483. Wijnans L, Dieleman J, Voordouw B, and Sturkenboom M. Effectiveness of pandemic influenza vaccination in a dutch population. Pharmacoepidemiol. Drug Saf. 2010; 19:S330. DOI: 10.1002/pds.2019
484. Paiva TM de, Ishida MA, Hanashiro KA, et al. Outbreak of influenza type A (H1N1) in Iporanga, Sao Paulo State, Brazil. Rev. Inst. Med. Trop. Sao Paulo 2001; 43:311–5
