# Supplementary material for: Comparison of 2 Doses vs 1 Dose in the First Season Children Are Vaccinated Against Influenza: A Systematic Review and Meta-Analysis
Source: JAMA Netw Open. 2025 Oct 3;8(10):e2535250. doi: 10.1001/jamanetworkopen.2025.35250 (PMC12495502; doi:10.1001/jamanetworkopen.2025.35250)
Supplement: Supplement 2. — Data Sharing Statement [file jamanetwopen-e2535250-s002.pdf]

Data Sharing Statement

Goldsmith. Comparison of 2 Doses vs 1 Dose in the First Season Children Are Vaccinated Against Influenza. *JAMA Netw Open*. Published October 03, 2025.
doi:10.1001/jamanetworkopen.2025.35250

Data
Data available: Yes
Data types: Data (not involving human participants)
How to access data: Data extracted from included studies and data used for the meta-analysis are available on reasonable request to the corresponding author (jggol@unimelb.edu.au).
When available: With publication

Supporting Documents
Document types: Statistical/analytic code
How to access documents: Associated statistical/analytic code are available on reasonable request to the corresponding author (jggol@unimelb.edu.au).
When available: With publication

Additional Information
Who can access the data: Anyone requesting the data.
Types of analyses: Validation and subgroup analyses.
Mechanisms of data availability: With investigator support.
Any additional restrictions: None
